# A porcine model of phenylketonuria generated by CRISPR/Cas9 genome editing

**DOI:** 10.1172/jci.insight.141523

**Published:** 2020-10-15

**Authors:** Erik A. Koppes, Bethany K. Redel, Marie A. Johnson, Kristen J. Skvorak, Lina Ghaloul-Gonzalez, Megan E. Yates, Dale W. Lewis, Susanne M. Gollin, Yijen L. Wu, Shawn E. Christ, Martine Yerle, Angela Leshinski, Lee D. Spate, Joshua A. Benne, Stephanie L. Murphy, Melissa S. Samuel, Eric M. Walters, Sarah A. Hansen, Kevin D. Wells, Uta Lichter-Konecki, Robert A. Wagner, Joseph T. Newsome, Steven F. Dobrowolski, Jerry Vockley, Randall S. Prather, Robert D. Nicholls

**Affiliations:** 1 Division of Medical Genetics, Department of Pediatrics, University of Pittsburgh School of Medicine, and Universityof Pittsburgh Medical Center (UPMC) Children’s Hospital of Pittsburgh, Pittsburgh, Pennsylvania, USA.; 2 Division ofAnimal Sciences, College of Agriculture, Food and Natural Resources, University of Missouri, Columbia, Missouri, USA.; 3 National Swine Research and Resource Center (NSRRC), College of Veterinary Medicine, University of Missouri, Columbia, Missouri, USA.; 4 Department of Pathology, University of Pittsburgh School of Medicine, Pittsburgh, Pennsylvania, USA.; 5 Department of Human Genetics, University of Pittsburgh Graduate School of Public Health, Pittsburgh, Pennsylvania, USA.; 6 Department of Developmental Biology, University of Pittsburgh, and UPMC Children’s Hospital of Pittsburgh, Pittsburgh, Pennsylvania, USA.; 7 Department of Psychological Sciences, University of Missouri, Columbia, Missouri, USA.; 8 GenPhySE, Université de Toulouse, INRAE, ENVT, 31326, Castanet-Tolosan, France.; 9 Department of Veterinary Pathobiology, College of Veterinary Medicine, University of Missouri, Columbia, Missouri, USA.; 10 Division of Laboratory Animal Resources, Office of Research, Health Sciences, University of Pittsburgh, Pittsburgh, Pennsylvania, USA.

**Keywords:** Genetics, Metabolism, Amino acid metabolism, Genetic diseases, Mouse models

## Abstract

Phenylalanine hydroxylase–deficient (PAH-deficient) phenylketonuria (PKU) results in systemic hyperphenylalaninemia, leading to neurotoxicity with severe developmental disabilities. Dietary phenylalanine (Phe) restriction prevents the most deleterious effects of hyperphenylalaninemia, but adherence to diet is poor in adult and adolescent patients, resulting in characteristic neurobehavioral phenotypes. Thus, an urgent need exists for new treatments. Additionally, rodent models of PKU do not adequately reflect neurocognitive phenotypes, and thus there is a need for improved animal models. To this end, we have developed *PAH*-null pigs. After selection of optimal CRISPR/Cas9 genome-editing reagents by using an in vitro cell model, zygote injection of 2 sgRNAs and Cas9 mRNA demonstrated deletions in preimplantation embryos, with embryo transfer to a surrogate leading to 2 founder animals. One pig was heterozygous for a *PAH* exon 6 deletion allele, while the other was compound heterozygous for deletions of exon 6 and of exons 6–7. The affected pig exhibited hyperphenylalaninemia (2000–5000 μM) that was treatable by dietary Phe restriction, consistent with classical PKU, along with juvenile growth retardation, hypopigmentation, ventriculomegaly, and decreased brain gray matter volume. In conclusion, we have established a large-animal preclinical model of PKU to investigate pathophysiology and to assess new therapeutic interventions.

## Abstract 2

A phenylalanine hydroxylase–deficient pig model of phenylketonuria is established and characterized.

## Graphical abstract

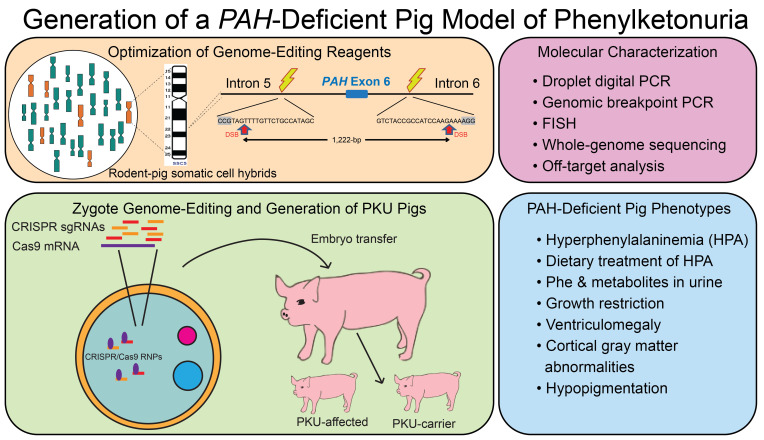

## Introduction

Phenylalanine hydroxylase–deficient (PAH-deficient) phenylketonuria (PKU) is the paradigm for treatable inborn errors of metabolism, motivating development of the first prospective newborn screening programs (1, 2). The natural history of PKU and the spectrum of mutations in the *PAH* gene are well documented (1–5). In untreated patients with complete PAH deficiency, there is neurotoxic accumulation of the essential amino acid phenylalanine (Phe) in blood, termed hyperphenylalaninemia (HPA), during early childhood development, which results in irreversible severe neurocognitive disability, neurological manifestations including seizures and spasticity, behavioral issues, eczema, and hypopigmentation (1, 2). This debilitating clinical presentation has largely been eliminated through newborn screening and early dietary Phe restriction (6, 7). However, despite over 50 years of early detection and treatment, PKU remains an unsolved problem. Adherence to dietary Phe restriction is poor in adolescents and adults, and neuropsychological testing has revealed persistent deficits in executive functioning, psychiatric symptoms, and attention deficit hyperactivity disorder in adults (8–11). In addition to psychiatric symptoms, adults with unrestricted Phe intake exhibit cognitive decline and white matter changes (8, 12). The maternal PKU syndrome is another manifestation of PKU, defined as an embryofetopathy precipitated by in utero Phe exposure occurring in offspring of PKU-affected women not practicing adequate Phe control during pregnancy. It is characterized by intellectual disabilities, microcephaly, and structural heart defects (13–15). Consequently, the development of novel therapies is critical to improve treatment of PKU-related disorders.

Interest in new therapies is evidenced by the recent approval of new medications for treatment of PKU and more than 100 clinical trials currently in progress, including gene therapy (ClinicalTrials.gov) (16, 17). Preclinical drug development typically entails an appropriate animal model; however, mouse models with Phe levels ranging from benign or mild HPA to classical PKU have serious limitations as the neurological phenotypes observed in humans are not seen even in the *Pah*^enu2^ classical PKU mouse model (18–20). Similarly, *Pah*^enu2^ mice are only modestly impaired in behavioral tasks, while mice with lower Phe homeostasis show no neurobehavioral deficits (18–21). Developmental and anatomical differences between the human and mouse brain likely account for this difference. Further, the FDA has demanded demonstration of neurocognitive benefit and not just Phe reduction in recent clinical trials for new PKU treatments. Thus, there is a critical need for a PKU animal model that faithfully recapitulates the human disease to move the therapeutic field forward.

We hypothesized that a large-animal PKU model, in which physiology is more similar to that of humans, may provide a superior preclinical PAH-deficient PKU model. Minipigs have a body and brain size, anatomy, physiology, metabolism, and genome sequence that correlate more closely with humans (22–25). Most notably, brain growth, development, and anatomy are similar for humans and pigs, as both are gyrencephalic in contrast to the lissencephalic brains of rodents (26–28). Minipigs are widely used for pharmaceutical and preclinical toxicity studies (29), and for behavioral and MRI studies (26, 27, 30–33), and provide valuable models of human disease that recapitulate clinical phenotypes and pathophysiological mechanisms (34–39). CRISPR/Cas9 is a versatile genome-editing system that streamlines and simplifies means to create models of human disease (25, 40–44). By using CRISPR/Cas9 gene editing, we have created a pig model of classical PAH-deficient PKU that recapitulates the biochemical and brain findings seen in untreated human patients with PKU, identifying it as a compelling model for future clinical studies.

## Results

### Design and optimization of Sus scrofa PAH CRISPR/Cas9 genome-editing reagents in an in vitro cell model.

In the last decade, the pig (*Sus scrofa*) genome sequence has been assembled for a reference breed (Duroc), thereby facilitating the use of molecular genetic tools, including those for genome editing (45). Through phylogenetic analyses of the genome sequences of Duroc as well as a diverse representation of swine breeds compared with the human *PAH* gene, we established that *S*. *scrofa* *PAH* comprises 13 exons and encompasses 75.1 kb (Supplemental Table 1 and Supplemental Figure 1A; supplemental material available online with this article; https://doi.org/10.1172/jci.insight.141523DS1) and encodes a 452–amino acid polypeptide that is phylogenetically highly conserved, with its sequence sharing 91.4% identity and 95.8% similarity to the human ortholog (Supplemental Figure 1B). As in humans, *PAH* is expressed predominantly in the liver, and to a lesser extent in kidneys in swine, with surprisingly high levels in the heart and detectable levels in other tested tissues by reverse transcription PCR (RT-PCR). No expression was detected in skeletal muscle (Supplemental Figure 1C). We then used PCR genotyping of a subpanel of 5 hamster-pig somatic cell hybrid (SCH) lines (46) to map the chromosomal location of *PAH*, with the presence in the SCH18 and SCH19 lines or absence in 3 other lines establishing a map position on the long arm of *S*. *scrofa* chromosome 5 (SSC5q) (Supplemental Figure 1D), consistent with the reference *S*. *scrofa* 11.1 genome build.

**Figure 1:**
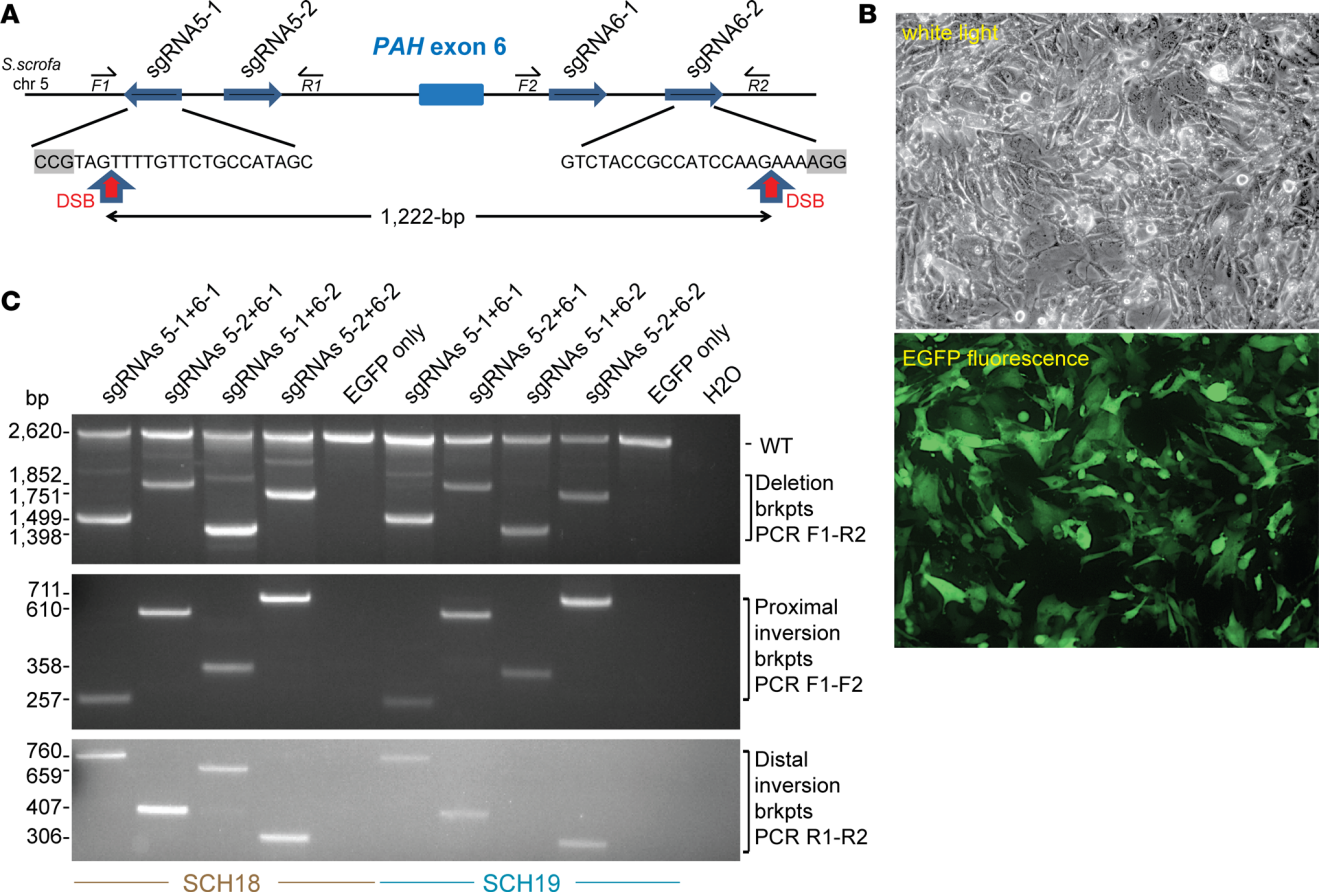
Strategy and optimization in vitro for CRISPR/Cas9 gene editing of the pig *PAH* gene. (**A**) Strategy showing paired CRISPR sgRNA targeting to generate deletion or inversion of *PAH* exon 6. Key: thick directional blue arrows, sgRNAs targeting the sense or complementary strand of intron 5 or intron 6; small directional arrows, forward (F) or reverse (R) deletion and inversion genotyping PCR primers; red vertical arrows, DSB sites in optimal sgRNA5-1 and sgRNA6-2 at a position 3 nt 5′ of the PAM motif (gray boxes). (**B**) Representative images (white light and EGFP epifluorescence, original magnification, ×10) for transfection of the SCH18 line with plasmids encoding Cas9, CRISPR sgRNA5-1 + sgRNA6-2, and EGFP. (**C**) Deletion and inversion PCR assays to detect exon 6 genome editing in SCH lines. Top: Deletion PCR assay with genotyping primers F1 and R2, for SCH18 and SCH19 cells transfected with pairs of sgRNA vectors plus EGFP or EGFP only as a control. Deletion breakpoints (brkpts) are only amplified in cells transfected with Cas9/sgRNA vectors whereas the WT band is detected in all samples. Middle and bottom: Inversion PCR assays for proximal and distal inversion breakpoints using primer pairs F1 and F2 or R1 and R2, respectively. Inversion breakpoints are detected in cells transfected with pairs of Cas9/sgRNA vectors indicating robust genome editing and NHEJ repair using the inverted exon 6 segment to bridge the DSB sites.

The use of a pair of CRISPR sgRNAs to target Cas9-induced double-stranded DNA breaks (DSBs) at 2 sequences in *cis* generates deletions or inversions of the intervening sequence (47). We chose to target pig *PAH* exon 6 because deletion (or inversion) of this exon leads to a frameshift after histidine 170 that creates a UGA nonsense mutation 44 codons later, resulting in early translational termination (Supplemental Figure 2, A and B) and predicted nonsense-mediated mRNA decay. This mutation was expected to generate a classical PKU disease (null) allele because deletions of exon 6 in humans result in classical PKU (48, 49). By using a CRISPR design tool (41) and optimal nucleotide (nt) selection for a 24 nt sequence spanning the 20 nt sgRNA and 3 nt protospacer adjacent motif (PAM) (5′-NGG-3′) (50), we selected 2 prospective sgRNA sites in *PAH* intron 5 and intron 6 (Figure 1A). To test the efficiency of CRISPR/Cas9 genome editing with the selected sgRNA pairs, we transfected (Figure 1B) CRISPR/Cas9 plasmid reagents (41) into the SCH18 and SCH19 lines (46), then performed deletion PCR assays on genomic DNA; only in cells transfected with the vectors expressing each CRISPR sgRNA pair and Cas9 was a PCR product of the expected size seen (Figure 1C, top). The intensity of the deletion breakpoint band with each of the 4 permutations of sgRNA pairs gave an estimate of the relative efficiency of editing, suggesting that the optimal guides were the combination of sgRNA5-1 and sgRNA6-2 (Figure 1C). Sanger sequencing of the deletion breakpoint PCR products confirmed the presence of locus-specific deletions for each sgRNA pair, clearly generated by DNA repair of 2 DSBs by a nonhomologous end-joining (NHEJ) mechanism with minor heterogeneity, including small insertion-deletions (indels) at the Cas9 DSB sites 3 nt upstream of the PAM (Supplemental Figure 3, A–D). In addition, inversion PCR assays (Figure 1C, middle and bottom) demonstrated the successful detection of specific bands for the proximal and distal inversion-breakpoints in each cell line transfected with pairs of CRISPR sgRNA/Cas9 vectors. Sequencing demonstrated that the recombinant breakpoints were repaired through NHEJ of the inverted targeted exon 6 region with minimal variation at the Cas9-induced DSB sites (Supplemental Figure 3, E–L). In conclusion, our strategy efficiently induced deletions and inversions at high frequency to generate null alleles of the pig *PAH* locus.

### CRISPR/Cas9 in vivo zygotic genome editing.

**Figure 2:**
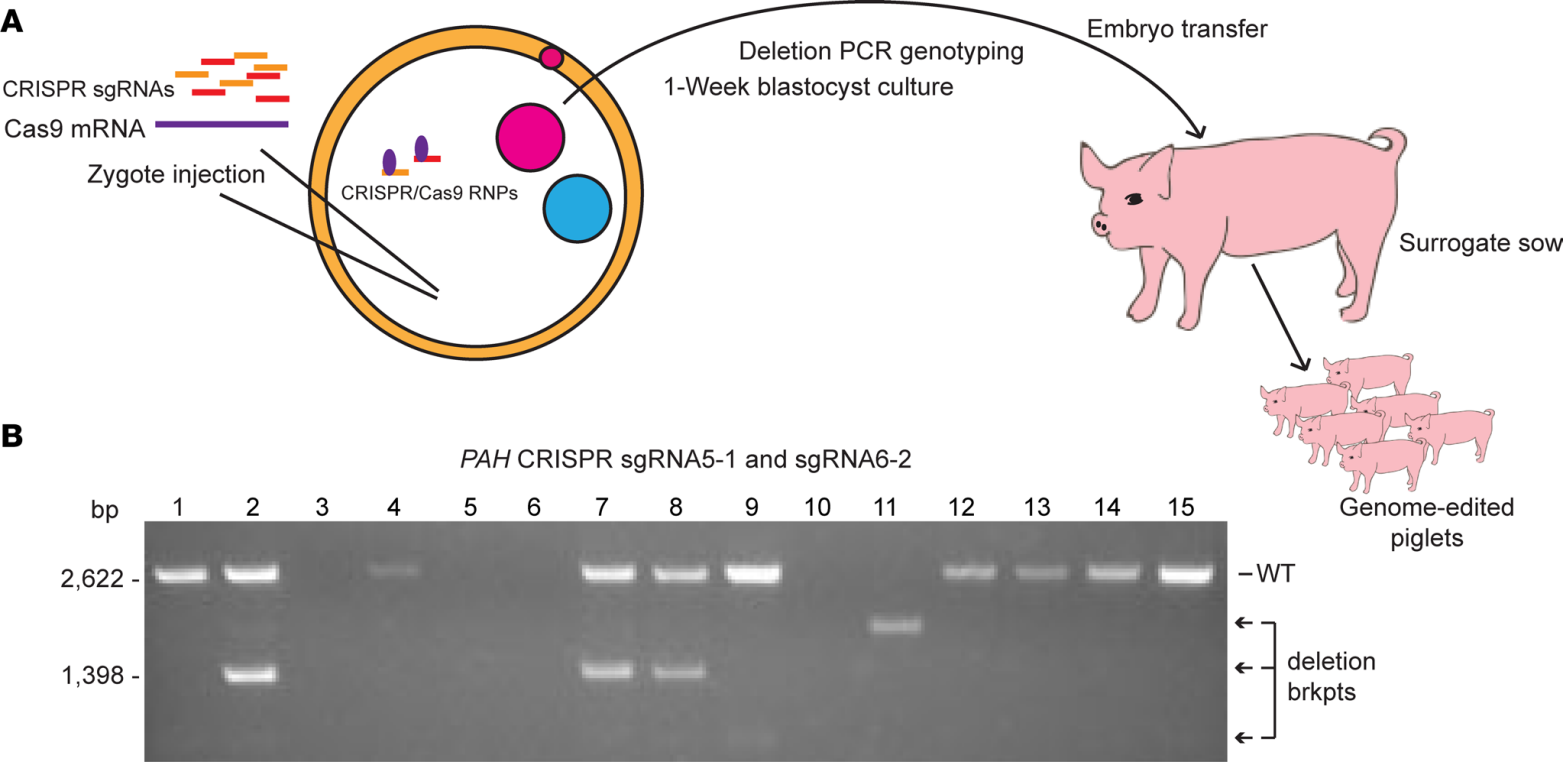
Zygote RNA injection, blastocyst screening, and embryo transfer to derive genome-edited pigs. (**A**) Strategy for in vivo genome editing of pig zygotes with injection of CRISPR sgRNAs and Cas9 mRNA into fertilized zygotes, and growth for 5–7 days in vitro, followed by DNA analysis of blastocysts or embryo transfer to a surrogate. RNP,. ribonucleoprotein. (**B**) Deletion PCR confirmation of gene editing of *PAH* exon 6 in a representative set of 15 pig blastocysts. PCR genotyping (as for Figure 1C) detects the WT band in 10 of these individual blastocysts while the expected deletion breakpoint (brkpt) band (1398 bp) is also detected in blastocysts 2, 7, and 8. Variant bands representing different deletion sizes are detected in blastocysts 9 and 11. Of a total of 57 blastocysts analyzed from 2 embryo transfers, 40 produced a PCR band(s), and of these 19 showed deletion alleles.

We next used CRISPR/Cas9 RNA reagents in preimplantation embryos (Figure 2A). Hybrid porcine zygotes of domestic and Yucatan minipig background were microinjected with Cas9 mRNA and the 5-1 and 6-2 sgRNAs in 2 experiments. Following 5 days of in vitro growth, blastocysts were either genotyped for the presence of genome-editing deletions or used for embryo transfer to a surrogate sow (Figure 2). Of 26 blastocysts screened by genomic deletion PCR from the first microinjection, at least 1 band was detected in 18; of these, 10 had either the expected deletion breakpoint fragment or an aberrant sized fragment. Similarly, we screened 31 blastocysts from the second round of microinjection, with at least one band detected in 22 and a deletion breakpoint fragment of the expected or aberrant size detected in 9 blastocysts (Figure 2B). Not all blastocysts yielded sufficient DNA for amplification of a PCR product; alternatively, absence of a band could represent larger deletions or other mutations (inversions or duplications) not detectable by this assay. These data imply a minimum frequency of approximately 48% blastocysts with a detectable *PAH* exon 6 deletion and indicate a high degree of in vivo genome editing by using a dual sgRNA CRISPR/Cas9 microinjection strategy. Two embryo transfer attempts were then made using separate batches of microinjected embryos, from which a single founding litter of PKU swine was successfully generated.

### Phenotypic characterization and dietary treatment of PKU swine.

Two genome-edited piglets, 116-1 and 116-2, were born from the second set of embryos transferred. Profiles of normal human and control Yucatan minipig plasma amino acid concentrations are similar (Supplemental Table 2), and therefore, we expected the PKU biochemical phenotype to be similarly pronounced. At 5 days of age, pig 116-1 had a blood Phe level in excess of 2000 μM and a Phe/Tyr ratio of 10.5, consistent with classical PKU (Table 1). Urinary Phe excretion was also high (Table 1). In contrast, littermate 116-2 had normal plasma amino acid concentrations (Table 1). In addition, urine organic acid assessments demonstrated that 116-1 excreted high levels of the Phe catabolite, phenylacetate, and Phe conjugates (*N*-acetylphenylalanine and phenylacetylglutamine), whereas 116-2 excreted very little of these products (Table 2).

**Figure 3:**
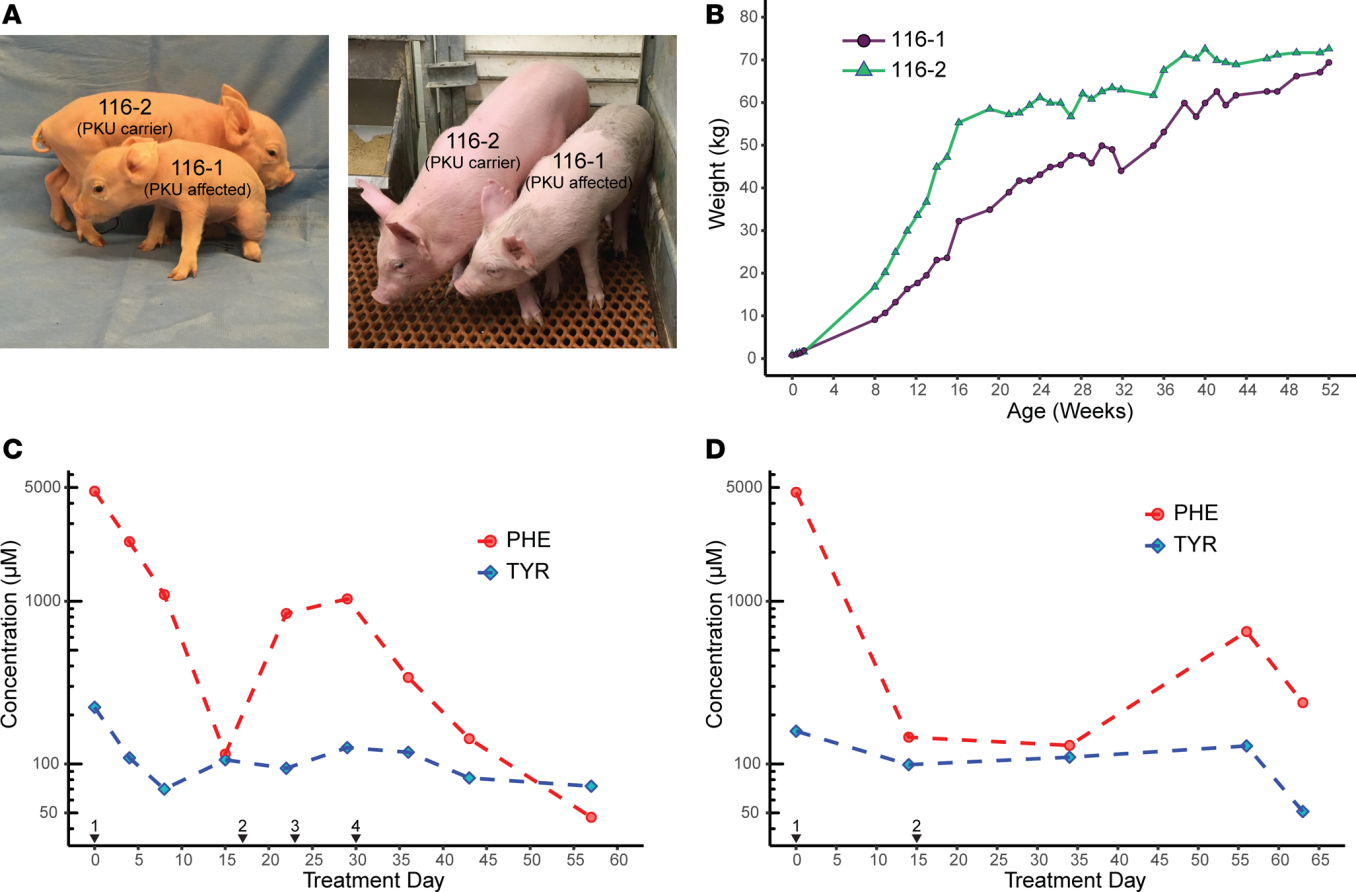
Phenotypes of affected and heterozygous PAH-deficient PKU pigs. (**A**) The PKU animal 116-1 had juvenile growth retardation and hypopigmentation compared with her heterozygous littermate 116-2. (**B**) Growth chart of affected pig 116-1 and heterozygous pig 116-2 pigs, demonstrating growth retardation in the former followed by catch-up growth toward 1 year of age. (**C**) Dietary treatment (trial 1) of the affected PKU pig results in reduction of blood Phe levels into therapeutic range and normalization of the Phe/Tyr ratio. Black arrowheads indicate days on which diet was changed to (1) initiation of Phe washout with 100% Phe-free chow, followed by adjustment of diet to (2) 50-50 normal to Phe-free chow; (3) 75-25 Phe-free to normal chow; and (4) 85-15 Phe-free to normal chow. Red circles, Phe levels; blue diamonds, Tyr levels. Amino acid concentration (μM, shown on a log_10_ scale) was assayed from blood plasma before and during dietary treatment as determined by HPLC. (**D**) Two-step dietary treatment (trial 2) of the affected PKU pig results in normalization of blood Phe levels and of the Phe/Tyr ratio. Details are as for **C** with (1) initiation of Phe washout with 100% Phe-free chow, followed by (2) adjustment of diet to 85-15 Phe-free to normal chow.

Two clear phenotypes in 116-1 (PKU) were hypopigmentation (Figure 3A) and growth retardation (Figure 3, A and B). Hypopigmentation is common in untreated PKU patients and is observed in *Pah*^enu2^ mice, which is thought to result from HPA impairing melanin production (51–53). Pig 116-1 had hypopigmentation on a large swath of her dorsal region. Neonatal growth retardation was observed in comparison of 116-1 and 116-2 at birth (0.76 kg compared with 1.02 kg) and continued postnatally through the first year of life (Figure 3B). However, her final adult weight was similar to her unaffected sibling. Postnatal growth retardation in 116-1 (Figure 3, A and B) could arise from 1 of 3 potential mechanisms (a) a large deletion including the flanking *IGF1* gene or intergenic enhancers (Supplemental Figure 4A), (b) segregating Yucatan alleles in F_1_ hybrid animals (because RNA-injected zygotes were generated by in vitro fertilization using sperm from a Yucatan minipig boar with oocytes from domestic pigs), or (c) as a consequence of neuronal toxicity of high Phe. To assess the first mechanism, we performed FISH by using BAC probes from a *PAH*-*IGF1* contig (Supplemental Figure 4A) and demonstrated that each of 2 tested BACs spanning this interval were intact on both chromosome homologs in 116-1 and 116-2 fibroblasts, without any decrease in FISH signal intensity (Supplemental Figure 4, B and C). In addition, the 116-1 PKU pig was growing at the same rate (Figure 3B) as Yucatan minipigs, which would imply a single major growth locus (or a very small number of loci) if the second mechanism explained body size in 116-1. However, this mechanism is unlikely because a major gene for body size has not been identified in Yucatan minipigs (54). Additionally, in multiple F_1_ animals derived from similar genome-editing experiments by using hybrid domestic × Yucatan minipig zygotes, differences of more than approximately 5 kg in body weights of offspring have not been observed (EMW, University of Missouri, unpublished data), consistent with the growth retardation of 116-1 not likely being the result of minipig genes. Therefore, we can conclude that body size in 116-1 was predominantly due to the third mechanism, chronic HPA toxicity during pre- and postnatal development.

To test whether HPA in 116-1 could be reduced by dietary Phe restriction, we performed 2 Phe washout and dietary restriction trials (Figure 3, C and D). In an initial trial, plasma Phe measurement taken before dietary treatment confirmed HPA in 116-1 (4746 μM). A dietary Phe washout was initiated with Phe-free diet supplemented with low Phe enrichment foods (Supplemental Table 3). Phe levels were reduced approximately by half (2324 μM) after 4 days, were reduced further at day 7 (1099 μM), and subsequently fell well into the therapeutic range (115 μM) by day 14 of complete Phe restriction. When Phe was added back into the diet with a 50-50 mix of Phe-free and complete chow, we observed an increase of Phe levels to outside the therapeutic range (Figure 3C). The diet was adjusted to account for the measured changes to a 75-25 and then an 85-15 mixture. After 5 days on the 85% Phe-free chow mixture, dietary Phe levels were back within the therapeutic range (<360 μM) and continued to decrease over the next 3 weeks into the normal range (Figure 3C). Following a lengthy period without dietary treatment, a subsequent second trial was performed with a 2-week Phe washout followed directly by switch to an 85-15 mixture of Phe-free and normal chow, which successfully maintained Phe levels within the therapeutic range (Figure 3D). As is observed with PKU patients on the therapeutic diet, there was some variability in plasma Phe concentration for 116-1 (Figure 3D), with higher Phe concentrations likely reflective of postprandial metabolism. After an initial fall in blood Tyr concentration during the Phe washout phases, Tyr levels subsequently stabilized during the final 85-15 dietary treatment regimen (Figure 3, C and D). Based on the amount of Phe consumed daily in the 75-25 (2380 mg) and 85-15 (1420 mg) diets, we calculated a Phe tolerance for 116-1 between 12 and 20 mg/kg/d, which is similar to many individuals with PKU (55). These results validate the pig as a preclinical model for PKU therapies with endpoints designed to lower Phe levels.

An in vivo brain MRI performed at 7 months of age showed that the PKU pig brain displayed largely normal gross anatomy. There were neither focal lesions nor punctate white matter changes observed in the PKU brain on T_2_-weighted and T_1_-weighted MRI (Supplemental Figure 5). Volumetric analysis revealed that compared with the control, the PKU pig brain had reduced volumes in the cortical gray matter and the white matter regions, subcortical regions, and cerebellum (Supplemental Table 4). While the 2 pigs were matched for age, sex, and body weight, they were not the same genetic background. Consequently, we normalized cortical volumes to the total cerebral volume and other volumes to total brain volume for each pig. Even with normalization, cortical gray matter volume in the PKU pig brain was reduced compared with the control pig brain, with no change in the normalized white matter volumes between the 2 pig brains (Supplemental Table 4), consistent with both pigs being of the same developmental age. Additionally, the PKU pig showed a reduction in normalized cerebellar volume compared with the control, while displaying a doubling in normalized ventricular volume (Supplemental Table 4), consistent with loss of periventricular brain tissue.

To examine neural microstructure of the PKU and control pig brains, diffusion tensor imaging (DTI) was performed; in particular, DTI is independent of brain size differences in the pigs. Only minor differences between the PKU and control pigs for cortical gray matter or white matter were detected (Supplemental Table 5) for axial diffusivity (AD; captures water diffusion along the neuronal axons), radial diffusivity (RD; demonstrates diffusivity perpendicular to the axons), apparent diffusion coefficient (ADC; reflects the physiological and pathological states of the tissue), and fractional anisotropy (FA; indicates axonal structural integrity).

Despite extraordinarily high levels of Phe and minor changes in brain composition, the affected PKU pig had no obvious neurological impairment. Neurological assessment showed that 116-1 responded to external visual, auditory and tactile stimuli; was fully ambulatory; and did not experience seizures. Cognitively, the affected pig was on par with her carrier littermate, being able to complete simple cognitive tasks, such as using a treat dispenser and slider puzzles. There was no discernable difference in these behaviors during Phe-restricted dietary treatment. Video recording of the affected animal shows that she successfully took nourishment, foraged in her pen with typical rooting behavior, interacted with enrichment objects, had normal diurnal sleeping patterns, and was socially aware of her surroundings.

### Molecular characterization of PAH deletion alleles in founding PKU animals.

**Figure 4:**
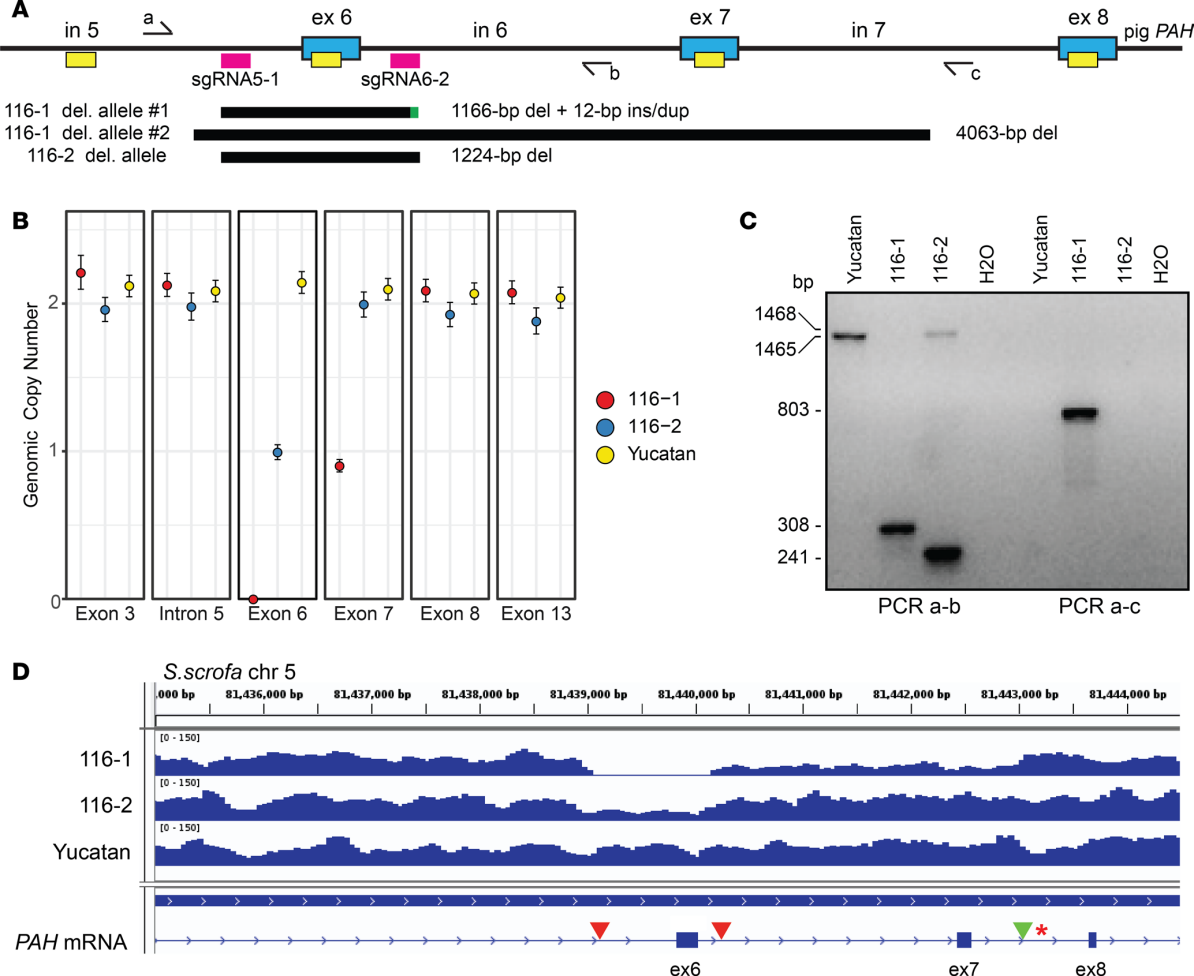
Molecular characterization of *PAH* deletion alleles in the pig PKU founder animals. (**A**) Schematic of CRISPR/Cas9 targeting of the *PAH* locus and detection of genome-edited alleles by ddPCR and long-range genomic PCR. Key: blue boxes, *PAH* exons (ex) with intervening introns (in); pink boxes, CRISPR sgRNA sites; yellow boxes, ddPCR amplicons; half arrows, genotyping primers a-c; black bars, regions deleted; green bar, insertion/duplication. (**B**) Genomic copy number across the *PAH* locus as determined by normalization of copies/μL to the single copy gene *GAPDH* (all amplitude plots are shown in Supplemental Figure 6), indicating heterozygosity at *PAH* exon 6 in 116-2 and homozygous exon 6 deletion along with heterozygous deletion of exon 7 in 116-1. Error bars were calculated based on the Poisson distribution. (**C**) PCR genotyping using primer pairs a-b to amplify a WT (1465 bp) fragment in Yucatan, a scarred allele (1468 bp) in 116-2, and exon 6 deletions in 116-1 and 116-2 (308 and 241 bp, respectively) and primer pair a-c to amplify the larger exon 6–7 deletion allele from 116-1 (803 bp). (**D**) Whole-genome sequencing (WGS) read depth coverage across the *PAH* exon 6–8 region in the founding PKU pigs and a Yucatan control. Note the absence of reads within exon 6 and reduced reads extending to intron 7 in the compound heterozygous deletion animal 116-1 and the reduced number of reads in exon 6 for the heterozygous animal 116-2. Red arrowheads mark the CRISPR sgRNA target sites, and the green arrowhead indicates the exon 6–7 distal breakpoint present on the larger 116-1 *PAH* deletion allele. The red asterisk represents an SNP linked to breakpoint reads used to infer the parental origin of the larger 116-1 deletion allele.

Deletion of *PAH* exon 6 was confirmed in the genome-edited offspring through a combination of copy number assessment by using droplet digital PCR (ddPCR) and deletion breakpoint PCR (Figure 4). We designed a ddPCR assay with 6 short PCR amplicons along the length of the *PAH* gene (Figure 4A and Supplemental Figure 6A). Amplification of ddPCR amplicon regions from genomic DNA input was detected by absorbance of intercalating EvaGreen dye with clear separation of positive droplets containing the genomic template and negative droplets that did not (Supplemental Figure 6, B–I). Notably, in 116-1 there was no amplification of exon 6, indicating apparent homozygous deletion in the affected animal (Supplemental Figure 6E). Additionally, there was diminished amplification of exon 6 in 116-2 and, surprisingly, of exon 7 in 116-1, suggesting heterozygosity at these locations (Supplemental Figure 6, E and F). Normalization of the concentration of positive droplets to a single copy locus, *GAPDH*, allowed determination of genomic copy number for all tested amplicons across the *PAH* gene in a control Yucatan minipig as well as in 116-1 and 116-2. All *PAH* amplicons were diploid (2n), with the exceptions of exon 6, which was nulliploid (0n) in 116-1 and haploid (1n) in 116-2, and exon 7, which was haploid in 116-1 (Figure 4B).

The use of 2 deletion breakpoint PCR assays both validated the ddPCR findings and fine mapped the breakpoint locations in 116-1 and 116-2. A primer set in intron 5 and intron 6 (PCR a-b) amplified the expected WT-sized fragment in the Yucatan control and from the intact chromosome in 116-2, but not 116-1, while different sized smaller deletion bands were detected in the 2 founder pigs (Figure 4C, left). This result was consistent with the expected genome-edited exon 6 deletion event but indicated molecular heterogeneity at the DNA sequence level. To detect the larger deletion allele in 116-1, the intron 5 PCR primer was used with one in intron 7 (PCRa-c), with specific detection of an exon 6–7 deletion breakpoint fragment (Figure 4C, right). No fragment was detected by PCRa-c in the control or 116-2 because of the extended length of the expected fragments. Combined, the ddPCR and deletion PCR results indicated that 116-1 was compound heterozygous for a *PAH* exon 6 and an exon 6–7 deletion and confirmed that 116-2 was heterozygous for an exon 6 deletion (Figure 4A).

### Genome-edited mutations in founding PKU animals.

**Figure 5:**
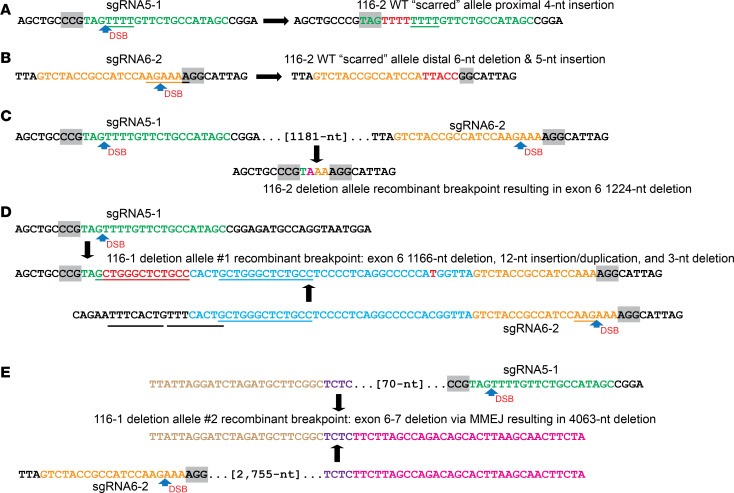
DNA repair mechanisms for deletion and scarred gene-edited *PAH* alleles. (**A** and **B**) The intact (WT-size) allele for 116-2 (heterozygote) demonstrates gene editing (“scarring”) at each sgRNA target site. Inaccurate NHEJ mechanisms lead to (**A**) the insertion of 4 T nucleotides (red) on DNA repair of a DSB at the sgRNA5-1 position, while (**B**) DNA repair of a DSB at the sgRNA6-2 position leads to both deletion of 6 nt (AGAAAA) including the first nucleotide of the PAM sequence and insertion of 5 nt of novel sequence (red; TTACC), respectively. (**C**) The deletion allele for 116-2 (heterozygote) arose by DNA repair of DSBs at each sgRNA position with loss of the intervening *PAH* exon 6 segment. Two extra nucleotides were deleted as part of the mechanism either due to noncanonical positioning of each DSB by CRISPR/Cas9, or, perhaps most likely, the inaccurate NHEJ mechanism of DNA repair. (**D**) A complex NHEJ mechanism accounts for the exon 6 recombinant deletion allele 1 in 116-1 (affected PKU pig). CRISPR/Cas9-induced DSBs at the canonical positions 3 nt 5′ of the PAM sequences for the sgRNA5-1 and sgRNA6-2 sgRNAs lead to a series of potential events that can be described by 2 alternative DNA repair mechanisms (see main text). Descriptively, the recombinant chromosome is characterized by a 1166 bp deletion starting at the sgRNA5-1 canonical position and with insertion at the deletion breakpoint of a novel 12 nt sequence apparently derived from an adjacent sequence to generate a 13 nt duplication (this and an adjacent 8 nt tandem repeat in the distal parental sequence are underlined). The distal deletion breakpoint is localized between the 13 nt duplication at a position 56 nt upstream of the canonical position for a DSB at sgRNA6-2, with a 3 nt deletion (AAG) at the latter DSB. A single SNP (C→T) arising in the mutant allele is also detected. (**E**) A complex MMEJ mechanism accounts for the larger exon 6–7 deletion allele 2 in 116-1 (PKU). DNA repair of the paired DSBs at sgRNA5-1 and sgRNA6-2 led to a recombinant breakpoint at a 4 nt (TCTC, purple) sequence of patchy homology located 70 nt 5′ and 2755 nt 3′ of the sgRNA target sites, respectively, resulting in a 4063 nt deletion.

We sequenced the deletion breakpoint PCR products for 116-1 and 116-2 (Figure 4C) as well as potential “scarred” alleles (56) at the 2 sgRNA target sites on the intact *PAH* allele of 116-2 to elucidate the mutations at genome-edited sites (Figure 5 and Supplemental Figure 7). For the latter, sequencing across each of the sgRNA5-1 and sgRNA6-2 target sites identified small indel mutations at each, indicative of DSB repair by NHEJ without rearrangement of the intervening fragment (Figure 5, A and B). At the proximal CRISPR sgRNA targeting site within intron 5, there was a 4 nt TTTT insertion at the predicted sgRNA5-1 DSB between the genomic sequence complementary to sgRNA nt positions 17 and 18 (i.e., 3 nt upstream of the PAM) (Figure 5A and Supplemental Figure 7A). At the distal intron 6 targeting site, there was a 6 nt deletion of the genomic sequence complementary to sgRNA6-2 nt 16 through the first nucleotide of the PAM and replacement by a 5 nt insertion of novel nucleotides (TTACC) (Figure 5B and Supplemental Figure 7B). These results show that the intact allele of the heterozygous carrier pig was scarred at each sgRNA target site during zygotic CRISPR/Cas9 genome editing.

The deletion allele present in 116-2 was generated through 1 of 2 near-canonical CRISPR/Cas9 genome-editing mechanisms (Figure 5C). In one possibility, both DSBs occurred between sgRNA positions 18 and 19 (i.e., 2 nt upstream of the PAM), and the intervening 1181 nt sequence including exon 6 was deleted. Alternatively, 1 DSB occurred between the complementary sequence to sgRNA5-1 positions 19 and 20 (i.e., 1 nt upstream of the PAM), and an expected canonical DSB occurred at sgRNA6-2 3 nt 5′ of the PAM, with the same sized intervening deletion. Due to the presence of an A at both the sgRNA5-1 position 18 and the sgRNA6-2 position 17, it was not possible to distinguish between these 2 similar events (Figure 5C and Supplemental Figure 7C).

Genome-editing events leading to the compound heterozygous deletion mutations in 116-1 (affected PKU pig) were more complicated and involved an insertion-duplication event in the exon 6 deletion allele 1 and a microhomology-mediated end-joining (MMEJ) event for the exon 6–7 deletion allele 2 (Figure 5, D and E). Two alternative mechanisms could account for the exon 6 deletion recombinant junction in 116-1 allele 1 (Figure 5D and Supplemental Figure 7D). A “sequential linear” explanation of events would suggest that following canonical DSBs at the 5-1 and 6-2 sgRNA sites, the distal 6-2 DSB was repaired immediately by NHEJ incurring a 3 nt deletion (i.e., AAG trinucleotide at sgRNA positions 15–17), but the proximal site sgRNA5-1 breakpoint was joined by NHEJ to a genomic sequence 56 nt upstream of sgRNA6-2 within intron 6, with insertion of a duplicated 12 nt sequence derived from the end of the retained distal sequence, resulting in a 1166 nt deletion. An alternative “DNA replication” mechanism for NHEJ invokes CRISPR/Cas9-induced DSBs at the canonical positions 3 nt 5′ of the PAM sequences for sgRNA5-1 and sgRNA6-2, leading to an expected 1223 bp deletion concluding with the 3 nt AAG sequence shown in underline in the parental sequence (Figure 5D), but DNA repair at the sgRNA6-2 position potentially by using an Okazaki DNA replication fragment (57) to link the broken chromosome ends led to a 65 nt insertion that was derived from 53 nt of intron 6 sequence plus de novo synthesis of 12 nt to generate a 13 nt direct repeat adjacent to the proximal breakpoint. Indeed, a DSB at the latter site may have arisen from “slipped mispairing” during DNA replication of an 8 nt tandem repeat (Figure 5D). In either mechanism, a transition mutation of a C to T at position –6 with respect to sgRNA6-2 was also detected (Figure 5D).

The second of the compound heterozygous alleles resulted in an unexpected deletion of exons 6 and 7 mediated through a 4 nt TCTC microhomology present in both intron 5 and intron 7 (Figure 5E and Supplemental Figure 7E). The microhomology sequence was located 70 bp upstream of the sgRNA5-1 PAM and 2755 bp downstream of the sgRNA6-2 PAM, in the middle of intron 7 (444 bp 3′ of the exon 7 splice donor and 639 bp 5′ of the exon 8 splice acceptor). The sequence of the 803 bp deletion PCR fragment (Figure 4C) spanned the *PAH* intron 5 sequence upstream of the microhomology and directly transitioned to intron 7 afterward. This indicates that a clean MMEJ mechanism repaired the 2 DSBs induced by dual targeting of *PAH* and resulted in a larger 4063 bp deletion including exons 6 and 7 (Figure 5E).

### Off-target analyses and whole-genome sequencing in founding PKU animals.

Although CRISPR/Cas9 genome editing is known to have high specificity because of the intolerance of mismatches in the sgRNA with complementary targeting sequence, there remains a possibility of off-target editing at other highly similar sequences. Based on an early sgRNA selection software by using repeat-masked genome sequence, we PCR amplified and directly sequenced the 8 and 10 top scoring potential off-target sites for sgRNA5-1 (Supplemental Figure 8) and sgRNA6-2 (Supplemental Figure 9), respectively. While several SNPs were detected, there was no evidence for genome editing across predicted off-target sites.

To provide a definitive analysis and confirmation of genome-editing events in the 2 founder genome-edited animals, we performed whole-genome sequencing (WGS) at 30× coverage along with a control Yucatan minipig. Structural variation analysis validated our findings of on-target *PAH* genome editing, detecting the previously identified exon 6 and exon 6–7 deletions (Figure 4D and Supplemental Table 6). Similarly, single nucleotide variation analysis validated the indels present in both the genome-edited exon 6 deletion alleles as well as the 2 scarred mutations in the intact allele of the heterozygote (Supplemental Table 7). Furthermore, an exhaustive search of all regions identified as potential off-target sites genome wide by the CRISPOR program (58) for sgRNA5-1 and sgRNA6-2 (129 and 63 potential off-target sites), we found no induced variants present (Supplemental Table 8). In summary, these WGS results indicated that our targeting of *PAH* exon 6 in swine was both efficient and accurate.

Intriguingly, based on WGS, a single nucleotide variant (SNV) was identified in intron 7 distal to the exon 6 and exon 6–7 deletion breakpoints (asterisk in Figure 4D) that differentiates between these alleles and informs the parental origin of the larger genome-edited deletion. The SNV is a length difference in a 10A (reference) or 11A (alternate) homopolymer tract, with 116-2 and Yucatan being homozygous for 11A and 116-1 (affected PKU pig) heterozygous for 10A/11A alleles (Supplemental Table 9). These data indicate that each founder PKU animal received a different maternal SNV allele present in the domestic oocyte donor pool. Furthermore, paired-end sequence reads in 116-1 established that 11A was linked to the intron 5 to intron 7 larger deletion breakpoint on allele 2 (Supplemental Figure 10A) while the 10A allele was linked only to the intron 7 DNA sequence (Supplemental Figure 10B). Combined, these data indicate for 116-1 that the 11A alternate variant marks the exon 6–7 deletion and represents the paternally derived Yucatan minipig allele, while the 10A reference variant represents the smaller exon 6 deletion residing on the maternally derived domestic allele.

## Discussion

Several genetic diseases have successfully been modeled in pigs after mouse models had proven unsuitable, including cystic fibrosis and Duchenne muscular dystrophy (38, 39, 59–61). By using genome editing, we have now established a pig model of PAH-deficient PKU. The index animals are biochemically consistent with and show physiological aspects of homozygous PKU and heterozygous carriers, respectively. Biochemically, our pig models classical PKU with extraordinarily high blood and urine Phe concentrations, along with secondary urinary markers of HPA. Intriguingly, Phe homeostasis in the affected pig was consistently greater than 4000 μM, which is greater than the *Pah*^enu2^ mouse (~2000 μM for males or ~2300 μM for females) and well in excess of untreated patients with classical PKU. Phenotypically, the affected pig had hypopigmentation and juvenile growth retardation but did not exhibit the devastating neurocognitive and neurological clinical characteristics of untreated PKU despite apparent brain MRI abnormalities. The reason for this discrepancy is unclear.

In recent years, MRI neuropathology studies have described pronounced white matter changes in early-treated PKU individuals with subsequent poor dietary control; however, there is growing recognition that gray matter may also be affected significantly (62, 63). Intriguingly, preliminary analysis of our pig model showed that the founder PKU pig had reduced gray matter volume, indicative of reduced neurogenesis, but no changes in the white matter. Ventriculomegaly in the PKU pig was consistent with this finding. Before the MRI, the PKU pig was untreated and displayed extreme HPA at all ages from birth. A finding of fewer neurons and less connectivity is consistent with untreated PKU in humans. Indeed, in the brains of untreated patients with PKU, arrested development of the cytoarchitecture of the cortical plate, a paucity of dendritic arborization and less synaptic spines in cortical neurons, and disturbed neuronal maturation have been observed, suggesting that the neuron was a primary target of the untreated disease (64). Planned postmortem analysis of the cytoarchitecture of the founder PKU pig cortex compared with her heterozygous littermate could validate and expand on our current MRI neuropathology studies. Furthermore, we plan longitudinal MRI, neurological, behavioral, cognitive, and neuropathological studies on early-treated and untreated experimental (PKU) and control (heterozygous and WT) cohorts from future generations of the PKU pigs. These studies will be critical to comparatively assess the neuroanatomical and functional correlates of PKU in the pig as a preclinical model and whether differences in treatment lead to neurologically distinct outcomes.

Neurological, behavioral, and imaging studies are preliminary, as only 1 affected animal was obtained, and hence not ideal to reveal milder physiological alterations. Basic neurological assessment revealed that the affected PKU pig 116-1 did not have any overt deficits in ambulatory motion or sensory awareness as compared with the carrier animal 116-2. Likewise, neurocognitive and neurobehavioral abnormalities were not observed in the affected animal, although no formal testing paradigms have yet been assessed. The lack of an overt neurological phenotype was an unexpected finding because the prolonged exposure of extreme HPA associated with classical PKU results in debilitating neurological and neurobehavioral clinical phenotypes. Our PKU pig has a hybrid Yucatan/domestic background, and it is possible that the phenotype is strain dependent. Because of animal size, our goal is to breed to a Yucatan minipig background, and this may provide additional insight into strain-dependent phenotypes. Alternatively, there may be sufficient differences in neuronal pathways not obvious by gross structure between porcine and human brains to preclude the effects of HPA seen in humans. We have not yet analyzed cerebrospinal fluid from the affected animal to measure Phe, Tyr, and neurotransmitter levels, which may provide further insight. Finally, there may be other genetic factors affecting CNS Phe metabolism or transport that are protective in the pig. Intriguingly, there are late-diagnosed and untreated PAH-deficient individuals who are refractory to HPA neurotoxicity and are not intellectually disabled (65, 66). Comparative genomic analysis between pigs and humans could be used to assess candidate genes controlling this putative protective effect.

The affected pig (pig 116-1) had both one expected size deletion and a second that was larger than expected, reflecting the robust propensity of NHEJ and MMEJ mechanisms of DNA repair of dual CRISPR/Cas9-induced DSBs to generate unexpected mutations at target sites in vivo (67, 68). Indeed, 2 genotyped blastocysts had an aberrantly sized deletion fragment on 1 allele but no WT band, suggesting the second allele had larger deletions than were detectable, indicating that aberrant DNA repair events are not uncommon. It is critical in disease modeling to fully characterize all genome-edited alleles to ensure that only the targeted gene is edited. We have demonstrated that larger-than-expected deletion alleles generated during dual sgRNA CRISPR/Cas9 deletion targeting can be efficiently screened for by quantitatively scanning copy number across a genomic locus with ddPCR, and as needed, supplemented by molecular cytogenetics. In our study each deletion was intragenic to *PAH*, and the gene product of each *PAH* allele in the founding generation was expected to be functionally null. Deletion of *PAH* exon 6 and presumptive splicing of exons 5 to 7 would result in a frameshift mutation with a truncated polypeptide lacking the majority of the enzymatic catalytic domain. The gene product of 116-1 *PAH* deletion allele 2 with ablation of exons 6 and 7 would generate an in-frame product with splicing of exons 5 to 8 (see Supplemental Figure 2C); however, the encoded 341–amino acid product would also lack a major portion of the catalytic domain encoded by exons 4–12 (69).

Although the profound HPA and other clinical phenotypes observed in the affected PKU pig were almost certainly due to the *PAH* mutations and unlikely to involve other genes through off-target mutation, we performed an exhaustive search through genomic PCR and WGS analyses to identify off-target hits. None of the top predicted targets were mutated in the genome-edited pigs, indicating the high fidelity of zygotic gene editing in targeting *PAH* with CRISPR/Cas9. These results were consistent with other studies showing the high specificity of in vivo CRISPR/Cas9 genome editing in swine and other mammalian species (70–72). While multiple high-throughput methods for off-target analysis have been developed, by genome sequencing at 30× coverage, we were able to detect both large structural variations (i.e., kilobase-sized deletions) and indels (i.e., scarred alleles and SNPs), that confirmed efficient *PAH* genome editing without off-target editing.

An important advantage of our novel PAH-deficient pig model is that new therapeutics can be assessed in a large-animal model, providing a greater measure of safety before human trials. Additionally, the pig PKU model may allow investigation of the pathophysiology of maternal PKU syndrome (13–15) with studies of neurological, cognitive, heart, and other phenotypes of offspring from untreated PKU-affected sows, topics not adequately addressed in either humans or mice. Further studies will determine the utility of the pig model for addressing teratogenic questions. Validation of additional clinical endpoints in affected animals, especially those focused on neurocognitive outcome, is needed for the real-world situation of imperfectly treated patients with relaxed treatment in adolescence and adulthood. This will open the door for development and evaluation in a pig preclinical model of next-generation therapeutics for PKU, including enzyme replacement/substitution, dietary supplements, hepatocyte/stem cell transplants, small-molecule and other pharmaceutical therapies, and genetic therapies.

## Methods

### PAH gene annotation and CRISPR/Cas9 targeting design.

Initial analyses of the pig *PAH* gene, by using the reference *S*. *scrofa* 10.2 genome build (45), detected 3 segments, not all in the correct order and orientation, and with exon 1 not annotated. Subsequently, we used BLAST (http://www.ncbi.nlm.nih.gov/blast/) comparisons to human *PAH* cDNA sequence to order and annotate 13 conserved exons, including the promoter exon 1 region. While ambiguities remain for *PAH* in the updated reference *S*. *scrofa* 11.1 genome build, because of single nt errors in 2 exons, these were corrected based on BLAST analysis detecting Duroc BAC sequences (CH242 library) and genome sequences from different breeds, including Jinhua, Wuzhishan, and others. Genomic PCR and RT-PCR primers (Supplemental Table 10) for *PAH*, for *GAPDH*, and across the *PAH*-*IGF1* intergenic region, as well as sgRNA selection, were designed from repeat-masked (http://www.repeatmasker.org/cgi-bin/WEBRepeatMasker) DNA sequences.

The original MIT CRISPR sgRNA design tool (see https://zlab.bio/guide-design-resources) (41), based on repeat-masked *S*. *scrofa* genome build 10.2, was used to identify potential sgRNA targets in *PAH* intron 5 and intron 6 sequences. The target sequences included the 20 nt sgRNA followed by the 3 nt PAM (5′-NGG-3′) for SpCas9 and were further ranked by both an unbiased nucleotide composition and by ensuring matches to preferred nucleotides at each position encompassing the optimal 24 nt sequences across targets (50). Oligonucleotides encoding the 20 nt target sgRNAs and *Bbs*I complementary overhangs (Supplemental Table 10) to generate cloning adapters were designed as described (41).

### Cell culture and in vitro genome editing.

SCH lines PLJ = 7, PLW1 = 8, PFA = 16, PFC = 18 (referred to as SCH18 here), and PFD = 19 (SCH19) were grown in RPMI 1640 medium with 10% FBS (46). Each hamster-pig SCH line retains an average of 4–7 pig chromosomes, with only pig SSC5 shared uniquely by SCH18 and SCH19, but not the other 3 SCH lines (PLJ = 7 carries SSC5p but not SSC5q). The CRISPR pX330 vector (Addgene 42230) encoding humanized *S*. *pyogenes* Cas9 (41) was used to clone oligonucleotides (Supplemental Table 10) specifying each CRISPR sgRNA into the *Bbs*I site, with confirmation by Sanger sequencing. Cell lines SCH18 and SCH19 plated in 6-well dishes were cotransfected with pairs of CRISPR sgRNA/Cas9 vectors and a pEGFP-C1 expression vector (500 ng per plasmid) using Lipofectamine 3000 in 1 mL OptiMEM (Thermo Fisher Scientific) and incubated overnight at 30°C. The medium was changed the following day, and GFP expression was checked at 24 hours to confirm transfection efficiency. To improve genome-editing yield, the cells were grown at 30°C (43, 73) for 4 days before harvesting. DNA was extracted from pelleted, transfected cells using the phenol/chloroform standard DNA extraction method. PCR for sequencing using primers that amplify the deleted or inverted region of DNA was done using Expand Long Template PCR System (Roche) following the protocol instructions.

### In vivo genome editing.

The generation of CRISPR/Cas9 genome-edited *PAH*-null swine was performed at the NSRRC, University of Missouri, by injecting reagents into zygotes generated from domestic breed oocytes fertilized by Yucatan minipig (RRID NSRRC:0012) sperm (44, 74). Trimethylated guanine-capped Cas9 mRNA (TriLink Biotechnologies) and *PAH* exon 6 targeting sgRNA5-1 and sgRNA6-2 were injected by using glass microinjection needles. Initially, genome-edited zygotes were grown in vitro to the blastocyst stage and genomic DNA was harvested. After confirming the presence of *PAH* exon 6 deletion PCR bands in a majority of injected blastocysts, an additional microinjection of zygotes was performed and cultured, and blastocysts were transferred to a surrogate domestic sow. A litter of 2 piglets, 116-1 and 116-2, were raised at the NSRRC until 1 year of age.

### Animal procedures.

While at the NSRRC, ear snips for fibroblast genomic DNA and cell cultures were taken from the neonatal pigs. Blood and urine collection at 5 days, 1 month, and 1 year were taken for amino acid metabolic profiling. An MRI of the affected PKU pig was performed at 7 months of age. The founding genome-edited PKU swine were transferred to the University of Pittsburgh at approximately 1 year of age. On arrival, and at least annually, animals were given a full physical examination. Animals were housed socially, were fed twice daily, had unlimited access to water feeders, and were given supplemental interactive enrichment. Blood collection from ear veins was performed with local anesthesia. Pig behavior within the pens over multiple days was monitored with a digital auditory-visual recording system (Noldus).

### Primary fibroblast isolation.

Primary dermal fibroblasts were isolated from the ear skin of newborn piglets and cultured by using established methods (75). Fibroblast cultures were grown in DMEM/F12 supplemented with 15% fetal bovine serum and 25 ng/mL basic FGF. A Yucatan minipig fibroblast line derived from a GFP-transgenic line (RRID NSRRC:0020) was grown as a control (75).

### Fluorescence in situ hybridization.

Cultured fibroblasts were processed for metaphase FISH by standard methods (76). FISH probes were prepared by labeling BAC (BACPAC Genomics) DNA using Nick Translation (Abbott Molecular Inc.), including CH242-225D12 with SpectrumOrange and CH242-131N2 with SpectrumGreen. Probe preparation, hybridization, and DAPI staining were by standard cytogenetics methods (76). All FISH analyses were performed on an Olympus BX61 epifluorescence microscope (Olympus Microscopes), with image capture and analysis using the Genus software platform on the Cytovision System (Leica Microsystems).

### Amino acid analysis.

Assessment of amino acids (urine, plasma) was performed in the UPMC Children’s Hospital of Pittsburgh Clinical Biochemical Genetics Laboratory using standard clinical means. Briefly, blood was drawn into sodium heparin tubes, and centrifugation was used to separate plasma. Random urine was collected for assessment. Both plasma and urine samples were frozen at –20°C before assessment. Lithium gradient ion exchange chromatography with Ninhydrin detection was used for amino acid assessment. A PerkinElmer A-10 system and a Pickering Ninhydrin reaction system were employed. Peak integration and quantification used standard clinical internal controls and clinical means.

### Urine organic acids.

Random urine samples were collected from the index animals for assessment of PKU-related analytes. Creatine quantification was performed by standard clinical means in the UPMC Children’s Hospital of Pittsburgh Clinical Chemistry Laboratory. A urine volume providing 1 mmol of creatinine was used in organic acid assessment. Internal standard (2-phenylbutyrate) was added to the urine sample that was subsequently saturated with sodium chloride. Sequential ethylacetate and diethyl ether extracts were performed; extracts were combined and dried under nitrogen. Trimethylsilane derivatization was performed. Analysis employed Agilent 7890A gas chromatogram with in-line Agilent 5979A mass spectrometer. Peaks were characterized by retention time and fragmentation pattern. Peak representation was normalized to the 2-phenylbutyrate internal standard.

### Dietary Phe restriction.

Dietary treatment of the adult affected PKU pig 116-1 began with a 2-week Phe washout in which the animal was fed specially made Phe-free chow (Supplemental Table 3). A blood collection was taken on day 14 to confirm reduction of Phe levels into the therapeutic range, and the diet was then switched to either a 50-50 Phe-free and regular chow (LabDiet Laboratory minipig grower diet DSC00021) mix on day 17 (trial 1) or an 85-15 mix on day 15 (trial 2). For the first trial, after a spike in blood Phe levels within days, the diet was subsequently adjusted to 75-25 mix on day 23 and then 85-15 mix on day 30, while after 4 weeks on the 85% Phe-free diet, a final blood collection and amino acid assessment were made; then dietary treatment terminated.

### Brain MRI and analysis.

Two pigs were subjected to brain MRI evaluation, 116-1 (female PKU; 7 months and 16 days of age, weight 44 kg) and a female large white cross as control (7 months and 21 days old, 50.5 kg). For brain imaging, after an overnight fast, sedation was initiated by intramuscular administration of ketamine (18 mg/kg) and midazolam (0.3 mg/kg); anesthesia was induced with 2 mg/kg (control) or 4 mg/kg (PKU) propofol and then maintained with a constant rate of infusion of ketamine (30 mg/kg/h) and midazolam (1.5 mg/kg/h). In vivo brain MRI was carried out on a 3T Siemens Trio scanner equipped with a standard 8-channel head coil. Axial and coronal fluid-attenuated T2-weighted 2D multislice imaging was acquired with a spin echo (SE) sequence with the following parameters: repetition time (TR)/echo time (TE)/inversion time (TI) = 9000/90/2500 ms, flip angle = 130°, field of view (FOV) = 24 cm, acquisition matrix = 256 × 256, slice thickness (SLTH) = 2 mm, and in-plane resolution = 0.9375 mm. 3D isotropic T1-weighted imaging was acquired with an inversion recovery sequence with the following parameters: TR/TE/TI = 2350/3.16/1000 ms, FOV = 25.6 cm × 25.6 cm × 17.6 cm, acquisition matrix = 256 × 256 × 176, SLTH = 1 mm, flip angle = 8°, and voxel size = 1 mm^3^. DTI was performed using 2D echo-planar (EP) diffusion-weighted sequence with the following parameters: TR/TE = 6400/104 ms, FOV = 23 cm, SLTH = 3 mm, acquisition matrix = 128 × 128, flip angle = 90°, with diffusion weighting at b  =  1000 s/mm^2^ in 68 diffusion orientations. Cortical gray matter, subcortical, white matter, ventricular, and cerebellar volumes were quantified by 2 independent observers using the open source segmentation tool ITK-SNAP (http://www.itksnap.org/pmwiki/pmwiki.php), and volumes were averaged. The DTI data were processed using DSI studio software (http://dsi-studio.labsolver.org/). Diffusion tensor matrices and principal eigenvalues were calculated for each voxel to generate multiplanar AD, RD, FA, and ADC maps covering the entire brain, with values reported as mean voxel values from gray matter and white matter (Supplemental Table 5).

### Breakpoint deletion and scarred allele PCR.

Genomic DNA from primary dermal fibroblasts derived from ear snips of a Yucatan control minipig and the 2 PKU pigs was isolated following proteinase K digestion by phenol-chloroform extraction and ethanol precipitation. Genomic DNA quality was assessed by analysis of 500 ng of DNA separated by 0.8% agarose Tris-acetate-EDTA–buffered gel electrophoresis and stained with ethidium bromide (EtBr). Subsequently, genomic DNA was quantified by high-sensitivity dsDNA Qubit fluorometric assay (Thermo Fisher Scientific). For *PAH* deletion and scarred allele PCR, 50 ng of genomic DNA served as a PCR amplification template using the Expand Long Template PCR System (Roche) and deletion or scarred allele-specific primers (Supplemental Table 10). PCR products were separated on a 1.0% agarose gel and stained with EtBr, gel extracted (Zymogen), cloned into the pJET1.2/blunt vector (Thermo Fisher Scientific), and Sanger sequenced on both forward and reverse strands (Genewiz).

### Droplet digital PCR.

Copy number across the *PAH* locus was assessed by EvaGreen-based ddPCR using the QX200 AutoDG Droplet Digital PCR System (Bio-Rad). Each ddPCR reaction for Yucatan minipig (control) and founding PKU littermates was run in duplicate using 5 or 25 ng of genomic DNA template and spiked with 2 U of *Eco*RI (New England Biolabs). Detection of PCR products derived from amplification of *PAH* exon 3, intron 5, exon 6, exon 7, exon 8, and exon 13, and a single copy unique sequence at a *GAPDH* upstream promoter as a reference (PCR primer sequences in Supplemental Table 10) was observed with clear separation of positive and negative droplets with manual cutoffs made in Quantasoft software (Bio-Rad). Genomic copy number across the *PAH* locus was calculated as the ratio of copy number per microliter relative to the reference *GAPDH* locus with 95% confidence intervals calculated based on the Poisson distribution (77).

### Off-target PCR-based analysis.

Potential off-target sites were initially identified at the time of sgRNA design (see above). For the top 10 scoring predicted off-target sites for each of sgRNA5-1 and sgRNA6-2, PCR products (primers in Supplemental Table 10) were amplified centered on the predicted off-target sites from control Yucatan minipig, PKU carrier, and affected genomes using Q5 high-fidelity DNA polymerase (New England Biolabs). Two of the predicted sgRNA5-1 off-targets were removed from the analysis due to genomic assembly errors. PCR products were separated by 2% agarose gel electrophoresis, gel extracted (Zymogen), and then directly Sanger sequenced (Genewiz). Sequence chromatograms were viewed with CLC Workbench (QIAGEN) and examined for possible off-target mutations. For most predicted off-target sites, analysis of direct sequencing results was straightforward. In some cases, a new internal sequencing primer was required to mask the effects of single nucleotide length variants, which caused dual waveforms in the sequence chromatograms.

### Whole-genome sequencing.

Genomic DNA was prepared from primary dermal fibroblast cell lines from 116-1, 116-2, and a Yucatan GFP-transgenic control (75) by phenol-chloroform extraction and ethanol precipitation of RNase cocktail–treated (Ambion) genomic DNA preps and dissolved in low Tris EDTA (10 mM Tris-HCl pH 7.6, 0.1 mM EDTA). Genomic DNA quality was assessed by Broad Range Genomic DNA TapeStation (Agilent) with all DNA integrity number scores greater than 9.0 and quantified by broad-range DNA Qubit (Thermo Fisher Scientific) with concentrations ranging from 150 to 250 ng/μL. Genomic sequencing libraries were prepared from DNA samples by acoustic fragmentation, end repair, adenylation, and ligation to sequencing adapters by using NEBNext Ultra reagents (New England Biolabs). Each pig was sequenced at 30× coverage with paired-end 150 bp Illumina reads (Genewiz). Bioinformatic analysis files, including demultiplexed reads (FASTQ), alignments of reads mapped to the Ensembl 11.1 *S.* *scrofa* reference (domestic Duroc breed) genome (BAM), and variant calls (VCF), were returned (Genewiz). Subsequently, sequencing reads mapped to the *PAH* exon 6 region were viewed in Integrative Genomics Viewer software (http://software.broadinstitute.org/software/igv/), and structural variants corresponding to the deletion region were extracted using high-throughput computational resources from the University of Pittsburgh Center for Research Computing. Additionally, SNVs located at any of 129 sgRNA5-1 and 63 sgRNA6-2 potential off-target sites identified by the CRISPOR program through http://crispor.tefor.net/ (58) in the unmasked Ensembl 11.1 *S*. *scrofa* genomic assembly were tabulated.

### Data availability.

The FASTQ files associated with WGS analysis have been deposited in the National Center for Biotechnology Information’s Short Read Archive under accession number PRJNA655516.

### Study approval.

The use of animals at NSRRC was approved by the University of Missouri Institutional Animal Care and Use Committee. All animal husbandry, care, and procedures while at the University of Pittsburgh followed an Institutional Animal Care and Use Committee–approved protocol, following the *Guide for the Care and Use of Laboratory Animals*, eighth edition (National Academies Press, 2011) (78). All animal work was performed at US Department of Agriculture–registered, NIH Office of Laboratory Animal Welfare–assured, and American Association for Accreditation of Laboratory Animal Care–accredited facilities and programs.

## Author contributions

EAK performed molecular work and designed and performed bioinformatics; BKR performed in vivo genome editing and molecular work in Missouri; MAJ, KJS, LGG, and MEY performed in vitro studies and mutation characterization; YLW and ULK analyzed MRI data; SEC performed MRI scans; DWL and SMG performed and interpreted molecular cytogenetic studies; MY provided SCH lines; AL developed dietary plans; LDS, JAB, SLM, MSS, EMW, SAH, and KDW contributed to pig veterinary and molecular studies in Missouri; JTN and RAW oversaw pig veterinary procedures in Pittsburgh; EAK, MAJ, and SFD contributed to pig procedures in Pittsburgh; SFD oversaw all biochemical studies; SFD and JV provided clinical expertise and support; RSP led all work in Missouri; RDN oversaw all aspects of the project and funding and with JV, RSP, and EAK designed phases of the study; EAK, BKR, SFD, JV, RSP, and RDN wrote the manuscript; and all authors contributed to the final manuscript.

## Supplementary Material

supplemental data

## Figures and Tables

**Table 1:**
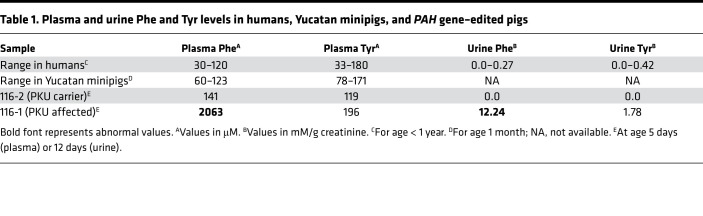
Plasma and urine Phe and Tyr levels in humans, Yucatan minipigs, and *PAH* gene–edited pigs

**Table 2:**
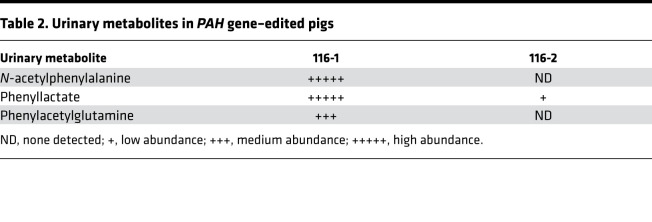
Urinary metabolites in *PAH* gene–edited pigs

## Version 1 10/15/2020

Electronic publication
