# Supplementary material for: A porcine model of phenylketonuria generated by CRISPR/Cas9 genome editing
Source: JCI Insight. 2020 Oct 15;5(20):e141523. doi: 10.1172/jci.insight.141523 (PMC7605535; doi:10.1172/jci.insight.141523)
Supplement: supplemental data [file jciinsight-5-141523-s144.pdf]

Supplemental Material

A porcine model of phenylketonuria generated by CRISPR/Cas9 genome editing

Erik A. Koppes[1], Bethany K. Redel[2], Marie A. Johnson[1], Kristen J. Skvorak[3,#], Lina Ghaloul-Gonzalez[1,4], Megan E. Yates[1], Dale W. Lewis[4], Susanne M. Gollin[4], Yijen L. Wu[5], Shawn E. Christ[6], Martine Yerle[7], Angela Leshinski[1], Lee D. Spate[8], Joshua Benne[8], Stephanie Murphy[8], Melissa S. Samuel[8], Eric M. Walters[2,8,^], Sarah A. Hansen[9], Kevin D. Wells[2,8], Uta Lichter-Konecki[1], Robert A. Wagner[10], Joseph T. Newsome[3,10], Steven F. Dobrowolski[3], Jerry Vockley[1,4], Randall S. Prather[2,8], Robert D. Nicholls[1]

[1] Division of Medical Genetics, Department of Pediatrics, University of Pittsburgh School of Medicine, and UPMC Children's Hospital of Pittsburgh, Pittsburgh, Pennsylvania, USA. [2] Division of Animal Sciences, College of Agriculture and Natural Resources University of Missouri, Columbia, Missouri, USA. [3] Department of Pathology, University of Pittsburgh, Pittsburgh, Pennsylvania, USA. [4] Department of Human Genetics, University of Pittsburgh Graduate School of Public Health, Pittsburgh, Pennsylvania, USA. [5] Department of Developmental Biology, University of Pittsburgh and UPMC Children's Hospital of Pittsburgh, Pittsburgh, Pennsylvania, USA. [6] Department of Psychological Sciences, University of Missouri, 210 McAlester Hall, Columbia, Missouri, USA. [7] GenPhySE, Université de Toulouse, INRAE, ENVT, 31326, Castanet-Tolosan, France. [8] National Swine Research and Resource Center (NSRRC) University of Missouri, Columbia, Missouri, USA. [9] Department of Veterinary Pathobiology, University of Missouri, Columbia, Missouri, USA. [10] Division of Laboratory Animal Resources, University of Pittsburgh, Pittsburgh Pennsylvania, USA.

Supplemental Material contains:

Supplemental Tables S1 – S10, pages 2-15

Supplemental Figures S1 – S10, pages 16-40

Supplemental References, page 40

Supplemental Tables

Supplemental Table S1. Exon-intron organization of the *PAH* gene in *Sus scrofa*[a].

Exon No.	Intron-exon boundary	Exon size (bp)	Exon-intron boundary	Intron Size (bp)
1	n/a	227[b]	TGGGGACAG***gt***gagccggg	5763
2	aatcctac***ag***GAAACAAGC	108	TTATTTGAG***gt***gagtgctt	13650
3	tcttttct***ag***GAGAATGGT	184	AAGACACAG***gt***aagagaca	13164
4	tgtgttgc***ag***TGCCCTGGT	89	GATCACCCT***gt***gagtgcac	13990
5	ttcttcct***ag***GGTTTTAAA	68	CTATCGACA***gt***aagtttac	7157
6	tgtatcct***ag***TGGGCAGCC	197	TTCTGCAGT***gt***gagtccac	2395
7	ttgcttcc***ag***CTTGCACCG	136	ACCTGAACC***gt***gagtactg	1087
8	tgtttttc***ag***TGACATCTG	70	TTTTCCCAG***gt***gaggaact	3403
9	ctcattac***ag***GAAATCGGC	57[c]	CTTGCAACA***gt***aagttccc	2433
10	tccatttc***ag***ATTTACTGG	96	GAATTACAG***gt***atgaccct	654
11	gggcctgc***ag***TATTGCTTG	134[d]	GAAAGTGAG***gt***gagctggc	3172
12	ttggtttc***ag***GAACTTTGC	116	CCATCAACG***gt***aagtaact	6161
13	ttctttgt***ag***GTGAAGTTG	622[e]	n/a	n/a

[a] Sscrofa 11.1 (Duroc) is the reference genome assembly. [b] Exon 1 includes 60-nt coding. [c] The Sscrofa11.1 assembly is missing 1-nt at a SNP (C/T), resolved by Duroc BAC sequences [CH242-225D12 (C), CH242-276L23 (T)]. [d] The Sscrofa11.1 assembly is missing 1-nt at a SNP (G/C), resolved by Duroc BAC sequences [CH242-225D12 (G), CH242-276L23 (C)]. [e] Exon 13 includes 44-nt coding (with the stop codon), with a total coding sequence of 1359-nt (including the stop codon). The total exon (mRNA) size is 2104-nt, with the 3'-end defined by BLAST searches of the EST database, for a gene length of 75133-nt in the Duroc breed.

Supplemental Table S2. Plasma amino acid levels in 1-month old Yucatan minipigs compared to human infants.

Amino acid	Range in human[+,#]	Range in Yucatan[+,^]
Ser	86-270	154-298
Ala	148-820	477-678
Gln	315-1060	261-374
Cit	10-70	69-136
**Phe**	**30-120**	**60-123**
**Tyr**	**33-180**	**78-171**
Val	80-330	314-633
Ile	20-100	158-308
Leu	40-180	158-349

[+] values in µM; [#] for age < 1 year; [^] for age 1 month.

Supplemental Table S3A. L-amino acid defined diet for swine without phenylalanine, from Dyets, Inc.

Dyets Inc.

2508 Easton Ave., Bethlehem, Pennsylvania 18017
Within 610 Area Code: 868-7701, FAX -868-5170
Outside 610 Area Code: 800-275-3938

FAX: 800-329-3938
E-Mail Address: Sales@dyets.com

DYET# 590007

L-Amino Acid Defined Diet for Swine without Phenylalanine

L-Alanine			11.9		
L-Arginine, fb			14		
L-Aspartic Acid			22.6		
L-Cystine			3.5		
L-Glutamic Acid			51.6		
Glycine			12.3		
L-Histidine, f b			5.4		
L-Isoleucine			8.2		
L-Leucine			16		
L-Lysine-HCl			11.4		
L-Methionine			3.5		
L-Phenylalanine			0		
L-Proline			14.4		
L-Serine			10.2		
L-Threonine			7.6		
L-Tryptophan			2.2		
L-Tyrosine			6		
L-Valine	kcal/g		9.5		kcal/kg
	4	total L-AA*……….	210.3		841.2
Ingredient				gm/Kg	
Sucrose	4			150	600
Cornstarch	3.6			275	990
Dyetrose	3.8			100	380
Corn Oil	9			150	1350
Cellulose	0			50	0
Salt Mix #290022	0.95			45	42.75
Sodium Bicarbonate	0			7.4	0
Vitamin Mix #390020	3.94			10	39.4
Choline Bitartrate	0			2.3	0
		total……..		1000.00	4243.35

Adapted from the NRC-Nutrient Requirements of Swine, 10th Ed., 1998
Dyets, Inc., December 7, 2017

Supplemental Table S3B. Low Phe enrichment foods.

LOW PHE ENRICHMENT FOODS FOR PKU PIGS		
Item	Amounts	PHE (mg)
Apple Rings	10 rings (~3/4 cup)	17
Apples	1 apple	11
Applesauce	1 cup	13
Blueberries	1/2 cup	18
Carrot	1/2 cup	18
Cranberries (Dried or Fresh/Frozen)	1/2 c	18
Cucumber	1/4 piece	15
Fruit Juice (Apple, Cranberry, Grape, Pineapple, Orange)	1 cup	2-30
Grapes	1/2 cup	15
Honey	2 T. Use sparingly	5
Ice Cream Cones	1 cone	16
Jelly	1/4 cup	17
Marshmallows	30 mini 1x/week	9
Papaya	1/2 c	18
Mushrooms	6	21
Pears	1 pear	20
Pumpkin	1/4 c raw OR 1/8 c canned	10
Radishes	4 small OR 2 lg	6
Romaine	1/2 cup	15
Strawberries	10 berries	14
Watermelon	½ cup	12

Supplemental Table S3C. Low Phe enrichment foods.

Item	Vendor	Stipulations?	Servings (Maximum daily)					
			<50 lbs.	50-100 lbs.	100-200 lbs.	200-400 lbs.	>400 lbs.	PHE (mg)
Routine Use								
Peas, Dried	Jordan		1/4 cup	1/2 cup	3/4 cup	1 1/2 cups	2 cups	218
Apple Rings	Jordan		10 rings (~3/4 cup)	10 rings (~3/4 cup)	10 rings (~3/4 cup)	10 rings (~3/4 cup)	10 rings (~3/4 cup)	17
Corn	Jordan		1/4 cup	1/2 cup	3/4 cup	1 1/2 cups	2 cups	163
Broccoli	Jordan		1 stalk-baseball size	1 stalk-baseball size	1 stalk-baseball size	1 stalk-baseball size	1 stalk-baseball size	177
Kale	Jordan		1 cup	1 cup	1 cup	1 cup	1 cup	96
Romaine	Jordan		1 cup	1 cup	1 cup	1 cup	1 cup	31
Occasional Use (Training, Etc. Treats)								
Fresh Produce								
Apples	Jordan		1/2 apple	1 apple	1 apple	1 apple	1 apple	11
Carrots	Jordan		1 carrot	1 carrot	1 carrot	1 carrot	1 carrot	37
Cucumber	Jordan		1/2 piece	1/2 piece	1/2 piece	1/2 piece	1/2 piece	29
Grapes	Jordan		1 cup/~30	1 cup/~30	1 cup/~30	1 cup/~30	1 cup/~30	29
Pears	Jordan		1/2 pear	1 pear	1 pear	1 pear	1 pear	20
Peppers, Green	Jordan		1 pepper	1 pepper	1 pepper	1 pepper	1 pepper	109
Potatoes	Jordan		1 small-3"	1 lg-5"	1 lg-5"	1 lg-5"	1 lg-5"	299
Potatoes, Sweet	Jordan		1 small-3"	1 lg-5"	1 lg-5"	1 lg-5"	1 lg-5"	116
Pumpkin	Jordan		1/4 small size raw, 1/8c canned	1/2 small size raw, 1/8 c. canned	1/2 small size raw, 1/4 c. canned	1/2 small size raw, 1/4 c. canned	1 small size raw, 1/4 c. canned	21
Radishes	Jordan		2 small/1 lg	4 small/2 lg	4 small/2 lg	4 small/2 lg	4 small/2 lg	6
Raisins	Jordan		1/4 cup	1/2 cup	3/4 cup	1 1/2 cups	2 cups	71
Zucchini	Jordan		1/2 piece	1/2 piece	1/2 piece	1/2 piece	1/2 piece	42
Dried Fruits, etc.								
Banana Chips	Jordan		1/2 cup/~20	1 cup/~40	1 cup/~40	1 cup/~40	1 cup/~40	73
Cranberries, Dried (Craisins)	Jordan		1/4 cup	1/2 cup	3/4 cup	1 1/2 cups	2 cups	18
Grocery								
Applesauce	GFS		1 cup	1 cup	1 cup	1 cup	1 cup	13
Cheerios	GFS		1 cup	1 cup	1 cup	1 cup	1 cup	176
Crackers (Saltine)	GFS	Use low salt versions	5	10	10	10	10	135
Dry pasta	GFS		1/4 cup	1/2 cup	3/4 cup	1 1/2 cups	2 cups	286
Fruit Juice	GFS		1 cup	1 cup	1 cup	1 cup	1 cup	2
Graham Crackers	GFS		1-2.5"x5" piece	2-2.5"x5" pieces	2-2.5"x5" pieces	2-2.5"x5" pieces	2-2.5"x5" pieces	97
Ice Cream Cones	GFS		1 cone	1 cone	1 cone	1 cone	1 cone	16
Honey	GFS		1 Tbsp. (ping pong ball size)	2 Tbsp. (ping pong ball size)	2 Tbsp. (ping pong ball size)	2 Tbsp. (ping pong ball size)	2 Tbsp. (ping pong ball size)	5
Jam/Jelly/Marmalade	GFS		1/4 cup	1/2 cup	1/2 cup	1/2 cup	1/2 cup	34
Jell-o	GFS		1/2 cup prepared; 1/4 pack dry	1 cup prepared; 1/2 pack dry	1 cup prepared; 1/2 pack dry	1 cup prepared; 1/2 pack dry	1 cup prepared; 1/2 pack dry	76
Marshmallows	GFS	Small amounts; use sparingly	15 mini; once/week	30 mini; once/week	30 mini; once/week	30 mini; once/week	30 mini; once/week	9
Oatmeal	GFS		1/4 cup	1/2 cup	3/4 cup	1 1/2 cups	2 cups	488
Popcorn	GFS	Use air popped ONLY, no microwave	1 cup	2 cups	2 cups	2 cups	2 cups	94
Vanilla Wafers	GFS		5	10	10	10	10	105

Supplemental Table S4. Volumetric analysis of the brain from T_1-weighted MRI in the affected PKU pig *vs.* female control pig. Volumes are averages from manual segmentation of two independent observers, with normalized volumes expressed as % of control and calculated by 1) cortical regions normalized to total cerebrum, and 2) cerebellum and ventricles normalized to total brain (normalized values greater than a 10% difference between pigs are in bold).

Brain Region	Brain Volumes (mm^3)		Normalized Volumes (%)
	Control	PKU	PKU
Cortical Gray Matter	36614	24568	**89**
White Matter	33052	25226	101
Subcortical Regions	22555	19912	**117**
Total Cerebrum	92221	69707	-
Ventricles	3053	4818	**209**
Cerebellum	12518	7060	**77**
Total Brain	107792	81585	-

Supplemental Table S5. Diffusion tensor imaging (DTI) parameters.

DTI Parameter	White Matter		Cortical Gray Matter	
	Control	PKU	Control	PKU
Apparent Diffusion Coefficient (ADC, $10^{-3}mm^2/s$)	0.75	0.76	1.09	1.05
Fractional Anisotropy (FA)	0.41	0.39	0.16	0.17
Axial Diffusivity (AD, $10^{-3}mm^2/s$)	1.08	1.08	1.24	1.21
Radial Diffusivity (RD, $10^{-3}mm^2/s$)	0.58	0.60	1.01	0.98

Supplemental Table S6. Structural variants (SVs) detected in the targeted *PAH* exon 6 region. WGS sequencing and SV analysis confirmed the deletion sizes of the two exon 6 deletions (116-1 deletion allele 1, 1166-nt; 116-2 deletion allele, 1224-nt) and for the extended exon 6-7 deletion (116-1 deletion allele 2, 4063-nt). Note that the 12-nt insertion-duplication in 116-1 allele-1, the TCTC microhomology of 116-1 allele-2, and the shared A at the 116-2 deletion breakpoint are also detected by this analysis.

Sample	Chr	Pos	Ref	Alt	End	SVLen	SVIns	SVInsSeq	HomLen	HomSeq
**116-1**	**5**	**81439023**	**G**	**DEL**	**81440189**	**1166**	**12**	**CTGGGCTCTGCC**		
**116-1**	**5**	**81438943**	**C**	**DEL**	**81443006**	**4063**			**4**	**TCTC**
**116-2**	**5**	**81439021**	**T**	**DEL**	**81440245**	**1224**			**1**	**A**

Supplemental Table S7. Single nucleotide variants (SNVs) detected in the targeted *PAH* exon 6 region associated with genome-editing events. Pertinent SNVs extracted from Variant Call Format (VCF) files derived from WGS and SNV analysis. At both the proximal sgRNA5-1 and distal sgRNA6-2 site SNVs associated with deletion and scarred alleles were detected and confirm genomic sequencing of deletion breakpoint and sgRNA site PCRs.

Sample	Chr	Pos	Ref	Alt	Qual	Info	Note
**116-2**	**5**	**81439023**	**G**	**GTTTT**	**89.14**	**AC=2;AF=1.000;**	**Scarred Allele 5-1 ins**
**116-1**	**5**	**81439024**	**T**	**C**	**35.02**	**AC=2;AF=1.000;**	**Aberrant SNP called where ins/dup starts at position PAM -4**
**116-1**	**5**	**81440223**	**C**	**T**	**74.08**	**AC=2;AF=1.000;**	**Induced SNP within retained intron 6 region of allele-1 ins/dup**
**116-1**	**5**	**81440242**	**CAAG**	**C**	**77.15**	**AC=2;AF=1.000;**	**116-1 allele-1 3-nt deletion at 6-2 sgRNA**
**116-2**	**5**	**81440242**	**CAAGAAA**	**C**	**50.01**	**AC=1;AF=0.500;**	**Scarred Allele 6-2 del**
**116-2**	**5**	**81440249**	**A**	**ATTACC**	**50**	**AC=1;AF=0.500;**	**Scarred Allele 6-2 ins**

Supplemental Table S8. Single nucleotide variants (SNV) detected at potential guide RNA off-target sites. (**A**) For sgRNA5-1, SNVs were identified in 14 of 129 potential off-target sites in 116-1, 116-2, or Yucatan. (**B**) For sgRNA6-2, SNVs were identified in 8 of 63 potential off-target sites in 116-1, 116-2, or Yucatan. Off-targets were predicted by CRISPOR.

Supplemental Table S8A. SNVs detected at potential sgRNA5-1 off-target sites.

Sample	CHROM	POS	ID	REF	ALT	QUAL	FILTER	INFO
116-1	1	52370840	.	T	A	48.85	PASS	AC=1;AF=0.500;AN=2;DP=30;FS=3.453;MQ=245.71;MQRankSum=3.837;QD=1.63;ReadPosRankSum=0.197;SOR=1.609;FractionInformativeReads=0.967;R2_5P_bias=7.319
Yuc:GFP	1	52370840	.	T	A	48.97	PASS	AC=1;AF=0.500;AN=2;DP=19;FS=1.820;MQ=250.00;MQRankSum=3.592;QD=2.58;ReadPosRankSum=-0.454;SOR=0.330;FractionInformativeReads=1.000;R2_5P_bias=9.942
116-1	1	52370858	.	T	C	49.03	PASS	AC=1;AF=0.500;AN=2;DP=28;FS=0.000;MQ=250.00;MQRankSum=4.367;QD=1.75;ReadPosRankSum=-0.366;SOR=0.951;FractionInformativeReads=0.964;R2_5P_bias=-4.227
Yuc:GFP	1	52370858	.	T	C	49.38	PASS	AC=1;AF=0.500;AN=2;DP=15;FS=0.000;MQ=250.00;MQRankSum=3.034;QD=3.29;ReadPosRankSum=-1.097;SOR=0.368;FractionInformativeReads=0.933;R2_5P_bias=6.313
116-2	1	164175680	.	T	C	50	PASS	AC=1;AF=0.500;AN=2;DP=45;FS=4.246;MQ=244.72;MQRankSum=4.766;QD=1.11;ReadPosRankSum=4.926;SOR=0.897;FractionInformativeReads=1.000;R2_5P_bias=17.303
Yuc:GFP	1	164175680	.	T	C	50	PASS	AC=1;AF=0.500;AN=2;DP=53;FS=0.000;MQ=246.00;MQRankSum=5.357;QD=0.94;ReadPosRankSum=3.185;SOR=0.739;FractionInformativeReads=1.000;R2_5P_bias=13.242
Yuc:GFP	1	164175681	.	G	A	50	PASS	AC=1;AF=0.500;AN=2;DP=54;FS=0.000;MQ=246.07;MQRankSum=5.423;QD=0.93;ReadPosRankSum=2.886;SOR=0.801;FractionInformativeReads=1.000;R2_5P_bias=14.630
116-1	2	118033142	.	G	A	49.92	PASS	AC=1;AF=0.500;AN=2;DP=27;FS=0.000;MQ=250.00;MQRankSum=4.392;QD=1.85;ReadPosRankSum=2.548;SOR=0.836;FractionInformativeReads=1.000;R2_5P_bias=-0.450
116-2	2	118033142	.	G	A	50	PASS	AC=1;AF=0.500;AN=2;DP=29;FS=0.000;MQ=250.00;MQRankSum=4.433;QD=1.72;ReadPosRankSum=3.969;SOR=0.941;FractionInformativeReads=0.966;R2_5P_bias=0.014
116-1	3	17468660	.	A	AG	127.11	PASS	AC=2;AF=1.000;AN=2;DP=31;FS=0.000;MQ=240.04;QD=4.10;SOR=0.765;FractionInformativeReads=0.871
116-2	3	17468660	.	A	AG	168.71	PASS	AC=2;AF=1.000;AN=2;DP=44;FS=0.000;MQ=236.49;QD=3.83;SOR=1.206;FractionInformativeReads=0.932
Yuc:GFP	3	17468660	.	A	AG	132.21	PASS	AC=2;AF=1.000;AN=2;DP=33;FS=0.000;MQ=249.26;QD=4.01;SOR=1.255;FractionInformativeReads=0.879
116-2	3	22588397	.	C	T	49.33	PASS	AC=1;AF=0.500;AN=2;DP=53;FS=1.035;MQ=250.00;MQRankSum=6.158;QD=0.93;ReadPosRankSum=1.074;SOR=0.859;FractionInformativeReads=0.981;R2_5P_bias=20.356
Yuc:GFP	3	22588397	.	C	T	46.4	PASS	AC=1;AF=0.500;AN=2;DP=48;FS=11.701;MQ=250.00;MQRankSum=5.755;QD=0.97;ReadPosRankSum=2.309;SOR=2.419;FractionInformativeReads=0.979;R2_5P_bias=12.812
116-1	3	123499714	.	A	G	50	PASS	AC=1;AF=0.500;AN=2;DP=53;FS=2.500;MQ=247.93;MQRankSum=4.799;QD=0.94;ReadPosRankSum=3.219;SOR=0.497;FractionInformativeReads=0.981;R2_5P_bias=15.112
116-2	3	123499714	.	A	G	107.03	PASS	AC=2;AF=1.000;AN=2;DP=21;FS=0.000;MQ=250.00;QD=5.10;SOR=1.721;FractionInformativeReads=0.952
Yuc:GFP	3	123499714	.	A	G	124.16	PASS	AC=2;AF=1.000;AN=2;DP=26;FS=0.000;MQ=250.00;QD=4.78;SOR=0.846;FractionInformativeReads=1.000
116-1	3	123926379	.	C	G	157.21	PASS	AC=2;AF=1.000;AN=2;DP=38;FS=0.000;MQ=250.00;QD=4.14;SOR=0.859;FractionInformativeReads=0.974
116-2	3	123926379	.	C	G	150.68	PASS	AC=2;AF=1.000;AN=2;DP=38;FS=0.000;MQ=250.00;MQRankSum=1.641;QD=3.97;ReadPosRankSum=-0.274;SOR=0.293;FractionInformativeReads=1.000;R2_5P_bias=0.000
Yuc:GFP	3	123926379	.	C	G	125.01	PASS	AC=2;AF=1.000;AN=2;DP=28;FS=0.000;MQ=250.00;QD=4.46;SOR=0.846;FractionInformativeReads=0.929
116-1	8	129310255	.	G	A	46.79	PASS	AC=1;AF=0.500;AN=2;DP=36;FS=1.434;MQ=247.77;MQRankSum=4.771;QD=1.30;ReadPosRankSum=-0.461;SOR=1.071;FractionInformativeReads=0.917;R2_5P_bias=15.497
116-2	8	129310255	.	G	A	49.47	PASS	AC=1;AF=0.500;AN=2;DP=17;FS=0.000;MQ=247.20;MQRankSum=3.281;QD=2.91;ReadPosRankSum=0.000;SOR=0.735;FractionInformativeReads=0.941;R2_5P_bias=8.303
116-1	9	11406822	.	C	CACG	32.53	PASS	AC=1;AF=0.500;AN=2;DP=11;FS=3.090;MQ=32.08;MQRankSum=0.678;QD=2.96;ReadPosRankSum=-1.246;SOR=0.078;FractionInformativeReads=1.000;R2_5P_bias=7.270
Yuc:GFP	9	11406822	.	C	CACG	51.63	PASS	AC=2;AF=1.000;AN=2;DP=5;FS=0.000;MQ=39.90;QD=10.33;SOR=1.981;FractionInformativeReads=1.000
116-1	9	11406824	.	AGGGAGG[illegible]	A	30.82	PASS	AC=1;AF=0.500;AN=2;DP=11;FS=7.368;MQ=32.08;MQRankSum=0.546;QD=2.80;ReadPosRankSum=-0.895;SOR=0.847;FractionInformativeReads=0.909;R2_5P_bias=17.501
Yuc:GFP	9	11406824	.	AGGGAGG[illegible]	A	48.93	PASS	AC=2;AF=1.000;AN=2;DP=5;FS=0.000;MQ=39.90;QD=9.79;SOR=3.258;FractionInformativeReads=0.800
116-2	9	11759440	.	C	T	50	PASS	AC=1;AF=0.500;AN=2;DP=44;FS=6.930;MQ=196.86;MQRankSum=2.575;QD=1.14;ReadPosRankSum=1.609;SOR=0.795;FractionInformativeReads=1.000;R2_5P_bias=-3.203
Yuc:GFP	14	20180376	.	T	C	50	PASS	AC=1;AF=0.500;AN=2;DP=24;FS=0.000;MQ=250.00;MQRankSum=4.128;QD=2.08;ReadPosRankSum=2.742;SOR=0.693;FractionInformativeReads=1.000;R2_5P_bias=7.553
116-1	15	29503986	.	A	G	131.1	PASS	AC=2;AF=1.000;AN=2;DP=28;FS=0.000;MQ=250.00;QD=4.68;SOR=0.836;FractionInformativeReads=1.000
116-2	15	29503986	.	A	G	137.1	PASS	AC=2;AF=1.000;AN=2;DP=30;FS=0.000;MQ=249.51;QD=4.57;SOR=1.329;FractionInformativeReads=1.000
Yuc:GFP	15	29503986	.	A	G	122.05	PASS	AC=2;AF=1.000;AN=2;DP=26;FS=0.000;MQ=250.00;QD=4.69;SOR=0.941;FractionInformativeReads=0.962
116-1	15	30672405	.	G	A	50	PASS	AC=1;AF=0.500;AN=2;DP=43;FS=0.000;MQ=250.00;MQRankSum=5.385;QD=1.16;ReadPosRankSum=1.575;SOR=0.627;FractionInformativeReads=0.953;R2_5P_bias=1.978
Yuc:GFP	15	30672405	.	G	A	49.47	PASS	AC=1;AF=0.500;AN=2;DP=40;FS=1.273;MQ=245.93;MQRankSum=5.166;QD=1.24;ReadPosRankSum=-2.210;SOR=0.657;FractionInformativeReads=0.925;R2_5P_bias=17.486
116-1	15	119050524	.	A	G	37.41	PASS	AC=1;AF=0.500;AN=2;DP=44;FS=1.438;MQ=250.00;MQRankSum=4.705;QD=0.85;ReadPosRankSum=2.335;SOR=1.165;FractionInformativeReads=0.818;R2_5P_bias=17.783
Yuc:GFP	15	119050524	.	A	G	50	PASS	AC=1;AF=0.500;AN=2;DP=38;FS=2.798;MQ=250.00;MQRankSum=5.102;QD=1.32;ReadPosRankSum=3.707;SOR=0.553;FractionInformativeReads=0.947;R2_5P_bias=-6.745
116-2	17	2120497	.	C	CA	50	PASS	AC=1;AF=0.500;AN=2;DP=27;FS=3.473;MQ=250.00;MQRankSum=4.367;QD=1.85;ReadPosRankSum=0.366;SOR=0.957;FractionInformativeReads=1.000;R2_5P_bias=-13.336
Yuc:GFP	17	2120497	.	C	CA	50	PASS	AC=1;AF=0.500;AN=2;DP=40;FS=1.302;MQ=249.01;MQRankSum=4.850;QD=1.25;ReadPosRankSum=0.535;SOR=0.502;FractionInformativeReads=0.925;R2_5P_bias=-9.032
116-1	17	2120498	.	A	AG	173.35	PASS	AC=2;AF=1.000;AN=2;DP=43;FS=0.000;MQ=246.00;QD=4.03;SOR=1.005;FractionInformativeReads=0.977
116-2	17	2120498	.	A	AG	49.06	PASS	AC=1;AF=0.500;AN=2;DP=27;FS=3.473;MQ=250.00;MQRankSum=4.367;QD=1.82;ReadPosRankSum=-0.464;SOR=0.819;FractionInformativeReads=1.000;R2_5P_bias=17.618
Yuc:GFP	17	2120498	.	A	AG	40.37	PASS	AC=1;AF=0.500;AN=2;DP=40;FS=1.302;MQ=249.01;MQRankSum=4.850;QD=1.01;ReadPosRankSum=-0.503;SOR=0.929;FractionInformativeReads=0.925;R2_5P_bias=18.493

Supplemental Table S8B. SNVs detected at potential sgRNA6-2 off-target sites.

Sample	CHROM	POS	ID	REF	ALT	QUAL	FILTER	INFO
116-1	2	17821848	.	C	T	49.03	PASS	AC=1;AF=0.500;AN=2;DP=36;FS=1.341;MQ=242.06;MQRankSum=3.168;QD=1.36;ReadPosRankSum=1.767;SOR=0.412;FractionInformativeReads=1.000;R2_5P_bias=10.199
116-2	2	17821848	.	C	T	44.49	PASS	AC=1;AF=0.500;AN=2;DP=59;FS=5.322;MQ=250.00;MQRankSum=6.390;QD=0.75;ReadPosRankSum=0.731;SOR=1.089;FractionInformativeReads=0.983;R2_5P_bias=-8.189
Yuc:GFP	5	30700216	.	C	T	49.65	PASS	AC=1;AF=0.500;AN=2;DP=21;FS=0.000;MQ=250.00;MQRankSum=3.723;QD=2.36;ReadPosRankSum=1.064;SOR=0.722;FractionInformativeReads=0.952;R2_5P_bias=-2.527
Yuc:GFP	5	30700221	.	T	C	49.58	PASS	AC=1;AF=0.500;AN=2;DP=21;FS=0.000;MQ=250.00;MQRankSum=3.723;QD=2.36;ReadPosRankSum=1.216;SOR=0.446;FractionInformativeReads=0.952;R2_5P_bias=4.477
116-1	6	66055371	.	T	C	50	PASS	AC=1;AF=0.500;AN=2;DP=38;FS=1.273;MQ=247.63;MQRankSum=5.166;QD=1.32;ReadPosRankSum=3.642;SOR=0.811;FractionInformativeReads=0.974;R2_5P_bias=-5.805
116-2	6	66055371	.	T	C	167.23	PASS	AC=2;AF=1.000;AN=2;DP=41;FS=0.000;MQ=250.00;QD=4.08;SOR=0.793;FractionInformativeReads=0.976
Yuc:GFP	6	66055371	.	T	C	212.01	PASS	AC=2;AF=1.000;AN=2;DP=56;FS=0.000;MQ=249.89;QD=3.79;SOR=0.729;FractionInformativeReads=0.982
116-1	8	115803643	.	A	G	140.17	PASS	AC=2;AF=1.000;AN=2;DP=32;FS=0.000;MQ=250.00;QD=4.38;SOR=0.756;FractionInformativeReads=0.969
116-2	8	115803643	.	A	G	50	PASS	AC=1;AF=0.500;AN=2;DP=43;FS=0.000;MQ=250.00;MQRankSum=5.509;QD=1.16;ReadPosRankSum=1.112;SOR=0.648;FractionInformativeReads=0.977;R2_5P_bias=-7.415
Yuc:GFP	9	7171091	.	T	G	50	PASS	AC=1;AF=0.500;AN=2;DP=50;FS=2.509;MQ=249.18;MQRankSum=5.881;QD=1.00;ReadPosRankSum=-0.291;SOR=0.745;FractionInformativeReads=0.960;R2_5P_bias=8.256
116-1	9	7171107	.	A	G	45.89	PASS	AC=1;AF=0.500;AN=2;DP=25;FS=0.000;MQ=250.00;MQRankSum=4.048;QD=1.84;ReadPosRankSum=0.991;SOR=0.653;FractionInformativeReads=1.000;R2_5P_bias=1.122
116-2	9	7171107	.	A	G	48.52	PASS	AC=1;AF=0.500;AN=2;DP=64;FS=0.000;MQ=250.00;MQRankSum=6.658;QD=0.76;ReadPosRankSum=0.893;SOR=0.794;FractionInformativeReads=0.953;R2_5P_bias=5.081
Yuc:GFP	9	7171107	.	A	G	193.69	PASS	AC=2;AF=1.000;AN=2;DP=51;FS=0.000;MQ=249.20;QD=3.80;SOR=0.818;FractionInformativeReads=0.961
116-1	9	16703942	.	T	A	46.13	PASS	AC=1;AF=0.500;AN=2;DP=38;FS=7.666;MQ=250.00;MQRankSum=4.981;QD=1.21;ReadPosRankSum=0.373;SOR=1.144;FractionInformativeReads=0.947;R2_5P_bias=16.448
116-2	9	16703942	.	T	A	49.71	PASS	AC=1;AF=0.500;AN=2;DP=31;FS=3.155;MQ=247.61;MQRankSum=4.635;QD=1.60;ReadPosRankSum=-1.226;SOR=1.329;FractionInformativeReads=0.968;R2_5P_bias=18.184
116-1	14	86614839	.	C	A	46.29	PASS	AC=1;AF=0.500;AN=2;DP=31;FS=0.000;MQ=250.00;MQRankSum=4.603;QD=1.49;ReadPosRankSum=3.832;SOR=0.602;FractionInformativeReads=1.000;R2_5P_bias=18.425
Yuc:GFP	14	86614839	.	C	A	50	PASS	AC=1;AF=0.500;AN=2;DP=25;FS=1.623;MQ=250.00;MQRankSum=4.048;QD=2.00;ReadPosRankSum=3.142;SOR=0.814;FractionInformativeReads=1.000;R2_5P_bias=-8.367
116-1	15	41392819	.	G	A	49.94	PASS	AC=1;AF=0.500;AN=2;DP=39;FS=0.000;MQ=250.00;MQRankSum=5.324;QD=1.28;ReadPosRankSum=1.840;SOR=0.610;FractionInformativeReads=1.000;R2_5P_bias=-4.389
Yuc:GFP	15	41392819	.	G	A	38.97	PASS	AC=1;AF=0.500;AN=2;DP=36;FS=3.329;MQ=250.00;MQRankSum=4.485;QD=1.08;ReadPosRankSum=2.762;SOR=0.947;FractionInformativeReads=0.917;R2_5P_bias=40.583

Supplemental Table S9. Single nucleotide variant (SNV) at SNP RS1108162262 distinguishing 116-1 deletion alleles. This SNP at chromosome 5:81443104 is a T in the reference (REF) genome and precedes an adjacent 10A/11A homopolymer length polymorphism. The latter 11A allele (the ALT SNP is written TA due to the additional A nucleotide adjacent to the T) is homozygous in both Yucatan and 116-2 (as shown by AC=2; AF=1.000 where AF is allele frequency). In contrast this SNP is heterozygous (AC=1; AF=0.500) in 116-1 (affected PKU pig).

Sample	Chr	POS	REF	ALT	QUAL	INFO
**116-1**	**5**	**81443104**	**T**	**TA**	**50**	**AC=1;AF=0.500**
**116-2**	**5**	**81443104**	**T**	**TA**	**126.13**	**AC=2;AF=1.000**
**Yuc:GFP**	**5**	**81443104**	**T**	**TA**	**125.32**	**AC=2;AF=1.000**

Supplemental Table S10. PCR primers and sgRNA oligonucleotides used in this study. (**A**) Off-target PCR primers [top 10 predictions for sgRNA5-1 (*PAH* intron 5) and sgRNA6-2 (*PAH* intron 6) using the original MIT CRISPR program]. (**B**) Pig *PAH* genomic and reverse transcription (RT)-PCR primers. (**C**) Other PCR primers. (**D**) *Bbs*I site oligonucleotide cloning adapters for sgRNAs.

Supplemental Table S10A. Off-target genomic PCR primers.

On-Target	Sequence
sgRNA5-1 (*PAH* intron 5)	CCGTAGTTTTGTTCTGCCATAGC
Reverse complement sgRNA5-1	GCTATGGCAGAACAAAACTACGG
sgRNA6-2 (*PAH* intron 6)	GTCTACCGCCATCCAAGAAAAGG

Off Target PCR Primer	Primer Sequence	Off Target Prediction	MIT score	mismatches	Sscrofa 11.1 location	Overlapping gene
PKU pig sgRNA5-1 off-target 1 F	5'-GACAAAATACTCCGAAACGCTG-3'	GCTATATCAGAACAAAACTAGAG	2.2	2MMs [6:7]	4: +88805601	ATF6
PKU pig sgRNA5-1 off-target 1 R	5'-GAGGCAGCTCTAACGAATGTG-3'					
PKU pig sgRNA5-1 off-target 2 F	5'-CTGTTGACAAGTATGAGTATGGC-3'	AATATGCCAGAACAAAACTATGG	1.7	3MMs [1:2:7]	16: +58034454	intergenic
PKU pig sgRNA5-1 off-target 2 R	5'-GTCAAGTTTCAGTGAGGACAAAC-3'					
PKU pig sgRNA5-1 off-target 3 F	5'-TCAAAATGTACCCAGAAACACAG-3'	TCCTGGGCAGAACAAAACTAAAG	1.3	4MMs [1:3:4:5]	13: +174649733	intergenic
PKU pig sgRNA5-1 off-target3 R	5'-AGAGGTACTGCACAGATGGCA-3'					
PKU pig sgRNA5-1 off-target 4 F	5'-TGACGTAGCTCACTTTCCTGG-3'	AAAGTGGCAGAACAAAACTAGAG	1.3	4MMs [1:2:3:4]	14: +27083917	intergenic
PKU pig sgRNA5-1 off-target4 R	5'-ATTCCTCCAAACGCCTTCCTC-3'					
PKU pig sgRNA5-1 off-target 5 F	5'-GTCATCAACCAAATCCACACCC-3'	GTTTTGCAAGAACAAAACTAAAG	0.9	4MMs [2:4:7:8]	6: -110017647	ZNF521
PKU pig sgRNA5-1 off-target 5 R	5'-GTCTCATTCCAATCCTGACACC-3'					
PKU pig sgRNA5-1 off-target 6 F	5'-ATGCTGATGAACACTGGAAGGA-3'	CGGATGCCAGAACAAAACTACAG	0.9	4MMs [1:2:3:7]	13: -98605057	MFSD1
PKU pig sgRNA5-1 off-target 6 R	5'-GTAGAAGGCTGGAAATTAGAGG-3'					
PKU pig sgRNA5-1 off-target 7 F	5'-AGTTGGTGGGTATTGGTGAGG-3'	GATCTAGAAGAACAAAACTAAGG	0.8	4MMs [2:4:6:8]	6: +105755628	intergenic
PKU pig sgRNA5-1 off-target 7 R	5'-AAACATAGGTATCAGAGGAAGGT-3'					
PKU pig sgRNA5-1 off-target 8 F	5'-TTTGTGTTTCTCCTCTTCCAGC-3'	TCAATGCCAGAAGAAAACTAAAG	0.4	4MMs [1:3:7:13]	8: +109168408	CAMK2D
PKU pig sgRNA5-1 off-target 8 R	5'-TCTCCTAGCCTCACATTCAGTC-3'					
PKU pig sgRNA6-2 off-target 1 F	5'-CCAAACTAGCCCCATGAACAG-3'	AGCTCCCTCCATCCAAGAAAAGG	1.4	4MMs [1:2:5:8]	1: +200728346	intergenic
PKU pig sgRNA6-2 off-target 1 R	5'-CTTTCTGTGCCAACCTCATTGC-3'					
PKU pig sgRNA6-2 off-target 2 F	5'-CATCCTTCCCTCCTCACTGC-3'	GGACACCACCATCCAAGAAACGG	1.3	4MMs [2:3:4:8]	11: -78854769	GAS6
PKU pig sgRNA6-2 off-target 2 R	5'-GTACCCTCGTCCCACATTCC-3'					
PKU pig sgRNA6-2 off-target 3 F	5'-GAGTTTCTGTGTGTCTCGTGGA-3'	GTATCCCCAATCCAAGAAAAGG	1.3	4MMs [3:5:8:10]	9: -7171086	PDE2A
PKU pig sgRNA6-2 off-target 3 R	5'-CCAAGTTTCCTTTCCCATTGCC-3'					
PKU pig sgRNA6-2 off-target 4 F	5'-GAACCTAACCCTCGGACGTGA-3'	GTCTGCTGTCATCCAAGAAAAAG	1	3MMs [5:7:9]	7: +3617295	intergenic
PKU pig sgRNA6-2 off-target 4 R	5'-GGCTCCCCTATGAAAAAGTCAC-3'					
PKU pig sgRNA6-2 off-target 5 F	5'-TAACGATTACTGTCATGCCTTGC-3'	TTCATCTGCCATCCAAGAAACAG	0.9	4MMs [1:4:5:7]	9: +28289522	MAML2
PKU pig sgRNA6-2 off-target 5 R	5'-ATCTGTACACTCCCTAGCATCC-3'					
PKU pig sgRNA6-2 off-target 6 F	5'-ACAGGAGAAGAGGGGACCTTC-3'	GGCCACGCCCATCCAAGAAATAG	0.9	4MMs [2:4:7:8]	7: -22858953	ENSSSCG00000001229
PKU pig sgRNA6-2 off-target 6 R	5'-TCTGTGTTGGGGGAGTAACTGT-3'					
PKU pig sgRNA6-2 off-target 7 F	5'-AGGTTTATTAACTTGGGGGAGTC-3'	GTCTCCCGCAATCCAAGAGAAAG	0.9	3MMs [5:10:19]	4: +85434777	LMX1A
PKU pig sgRNA6-2 off-target 7 R	5'-GTGGTAACTAGAGGAGGAAAGG-3'					
PKU pig sgRNA6-2 off-target 8 F	5'-GCTGGACATGAATGAGTTTGAC-3'	GGCTTCCTCCAACCAAGAAAGAG	0.7	4MMs [2:5:8:12]	2: +61069006	CHERP
PKU pig sgRNA6-2 off-target 8 R	5'-GATCAAGTAGATGAGGTGCAGC-3'					
PKU pig sgRNA6-2 off-target 9 F	5'-CCCATTTTTCTCTGCTTGAGCC-3'	GCCTCCAGGCATCCAAGAAATGG	0.6	4MMs [2:5:7:9]	14: +86614855	GRID1
PKU pig sgRNA6-2 off-target 9 R	5'-CAATCCACATGCTGATTGCTGG-3'					
PKU pig sgRNA6-2 off-target 10 F	5'-AGTATACATGGACATCTACTGCA-3'	GTTTAAATCCATCCAAGAAATAG	0.5	4MMs [3:6:7:8]	1: -47850261	intergenic
PKU pig sgRNA6-2 off-target 10 R	5'-CTGTGGTATAGTCTCATATGGAG-3'					
PKUofftarget6-2_3seq	5'-TGGACCCAGGTCCAGAAC-3'	n/a	n/a	n/a	n/a	
PKUofftarget6-2_10seq	5'-GTAGTTATTTAATTGTGC-3'	n/a	n/a	n/a	n/a	
PKUofftarget6-2_3seq(2)	5'-CCCCAGGAGGCTTAGAGA-3'	n/a	n/a	n/a	n/a	

Supplemental Table S10B. *PAH* genomic and RT-PCR primers.

PAH PCR Primer	Primer Sequence
pig PAH ex 2 RT-PCR F	5'-TCCAGTCAAGATGGTTCCATA-3'
pig PAH ex 3 RT-PCR R	5'-TCTGACAGAGTAGGCATGCTG-3'
pig PAH ex 3 RT-PCR F	5'-CAGCATGCCTACTCTGTCAGA-3'
pig PAH ex 3 RT-PCR R	5'-CCGGGAGAGCTCGTGCACG-3'
pig PAH intron 5 F	5'-CAAAACATGTAGTTTAACAAGCTG-3'
pig PAH intron 5 R	5'-CACCTCACTCAGAGCACCAG-3'
pig PAH intron 6 F	5'-GAAGAAAGTAAATTGAGGCCAAG-3'
pig PAH intron 6 R	5'-GTACTTTTCAGGATCATGGAAATG-3'
pig PAH exon 5 F	5'-CGAACAAGACGGAAGTACTTTG-3'
pig PAH exon 7 R	5'-GAAATCCCGAGACGAAAGCAG-3'
pig PAH inversion R	5'-AAGTAAATTGAGGCCAAGGTGC-3'
pig PAH intron 5 R	5'-CCAGTATGGGAATTCACTTACC-3'
pig PAH intron 5 R	5'-AATGCATGGTAGGTCTAAGATC-3'
pig PAH intron 5 F	5'-CTCTTAGATGGTTTTCAAAAGG-3'
pig PAH int5 LR-PCR F	5'-GAGTAGTTGCAGCACAGGTGG-3'
pig PAH int6 LR-PCR R	5'-GTCTCTTGGCACAAACAGCAC-3'
pig PAH int5 LR-PCR F2	5'-CTATTGAGTCATAGCATCACGG-3'
Pig PAH exon 6 STS F	5'-ACGCCTGCTACGAGTACAATC-3'
Pig PAH exon 6 STS R	5'-AGATGTGGACTCACACTGCAG-3'
Pig PAH exon 7 STS F	5'-TAGCACTGCCCTGCCTCAG-3'
Pig PAH exon 10 STS F	5'-CAGATTTACTGGTTTACTGTGG-3'
Pig PAH exon 10 STS R	5'-ATTCACCAAAGGATGACAGGAG-3'
Pig PAH exon 13 STS F	5'-GCAGAACCTCATCTGTCAGC-3'
Pig PAH exon 13 STS R	5'-TACTTGTTGATCCTCCATCTTG-3'
Pig PAH intron 5 STS F	5'-GACACTGTGTACCCTGTTAG-3'
Pig PAH intron 5 STS R	5'-TTTGAAAACCATCGAAAGTTCC-3'
pig PAH intron 5 F	5'-AGTGGTGCATCTCTAGGCAAG-3'
pig PAH exon 13 (3'-UTR) R	5'-CAGTAATTAGAATCACAGTCCC-3'
pig PAH intron 3 R	5'-GGCACCTTTAGACACCCAAATC-3'
pig PAH intron 6 F	5'-CTCGTCTCAAATGCTGCAACG-3'
Pig PAH intron 7 F	5'-TGCACAGAAAGCCTGAGTCTG-3'
Pig PAH exon 8 R	5'-GTACATGTCCCAACAGCTCATG-3'
Pig PAH intron 8 F	5'-CTGTGCCAGTGACTGTCTTTC-3'
Pig PAH exon 9 R	5'-TTCTCAATATACTCGTCAGGTGC-3'
pig PAH intron 5 breakpoint F	5'-GAACACAGCTCCCTCTTAGATG-3'
pig PAH intron 6 breakpoint R	5'-CATGGAAATGGCAAAATCCACAG-3'
pig PAH Intron 5 ddPCR STS F	5'-ATGATTCTAAGGTAAACAGCCTC-3'
pig PAH Intron 5 ddPCR STS R	5'-CATCTAAGAGGGAGCTGTGTTC-3'
pig PAH intron 5 F-2	5'-TGTTCAGAACACAGCTCCCTC-3'
pig PAH intron 6 R-2	5'-ACCCAGACTTGTTAGTCACTTTG-3'
pig PAH intron 5 del-breakpoint F	5'-CTTGTCTTGCTTTCAGTTCTTC-3'
pig PAH intron 5 del-breakpoint R	5'-CACAGAACACGCCACTTAT-3'

Supplemental Table S10C. Other PCR primers.

Other PCR Primer	Primer Sequence
pig GAPDH qRT-PCR F	5'-ATCCTGGGCTACACTGAGGAC-3'
pig GAPDH qRT-PCR R	5'-CTTGACGAAGTGGTCGTTGAGG-3'
pig GAPDH control F ddPCR	5'-CCGCGATCTAATGTTCTCTTTC-3'
pig GAPDH control R ddPCR	5'-TTCACTCCGACCTTCACCATC-3'
pig IGF1 exon 1a STS F	5'-TTCAGAGCAGATAGAGCCTGC-3'
pig IGF1 exon 1a STS R	5'-AATCCAGGGAGATGGGAGATG-3'
pig IGF1 exon 1b STS F	5'-TGACTCAAATCCCAGCTGTGC-3'
pig IGF1 exon 1b STS R	5'-AGCATCTAGTTACATTGGGAC-3'
pig PAH-IGF1 intergenic CpG1 F STS F	5'-GTTTACTCAACAGAACGCTGTC-3'
pig PAH-IGF1 intergenic CpG1 F STS R	5'-TTCTCAGTTGGCTCTGTACATG-3'
pig PAH-IGF1 intergenic CpG2 F STS F	5'-GAAGATGAAGCTAGGTTGATCG-3'
pig PAH-IGF1 intergenic CpG2 F STS R	5'-TCAGTAGGATTTCTGAGTTAGTC-3'
pig PAH-IGF1 intergenic 1 (R8-9 enhancer) STS F	5'-GTTGTATGGCGTGAGCTACAG-3'
pig PAH-IGF1 intergenic 1 (R8-9 enhancer) STS R	5'-GGCAGCCTGTTATTTTACGAG-3'
pig PAH-IGF1 intergenic 2 (R13 enhancer) STS F	5'-TACAGATCCAATGGTAGCTCC-3'
pig PAH-IGF1 intergenic 2 (R13 enhancer) STS R	5'-TTCACAAGGCGACAGAGCAG-3'

Supplemental Table S10D. *Bbs*I site oligonucleotide cloning adapters for sgRNAs.

sgRNA Oligo	OligoSequence
sgRNA pig PAH intron 5-1 sense	5’-CACCgGCTATGGCAGAACAAAACTA-3’
sgRNA pig PAH intron 5-1 complementary	5’-AAACTAGTTTTGTTCTGCCATAGCc-3’
sgRNA pig PAH intron 5-2 sense	5’-CACCgGGTAAGTGAATTCCCATACT-3’
sgRNA pig PAH intron 5-2 complementary	5’-AAACAGTATGGGAATTCACTTACCc-3’
sgRNA pig PAH intron 6-1 sense	5’-CACCgGAGTGCGTTTGTGCTGATGC-3’
sgRNA pig PAH intron 6-1 complementary	5’-AAACGCATCAGCACAAACGCACTCc-3’
sgRNA pig PAH intron 6-2 sense	5’-CACCgGTCTACCGCCATCCAAGAAA-3’
sgRNA pig PAH intron 6-2 complementary	5’-AAACTTTCTTGGATGGCGGTAGACc-3’

Supplemental Figures

Supplemental Figure S1. Characterization of the *PAH* gene in *Sus scrofa*.

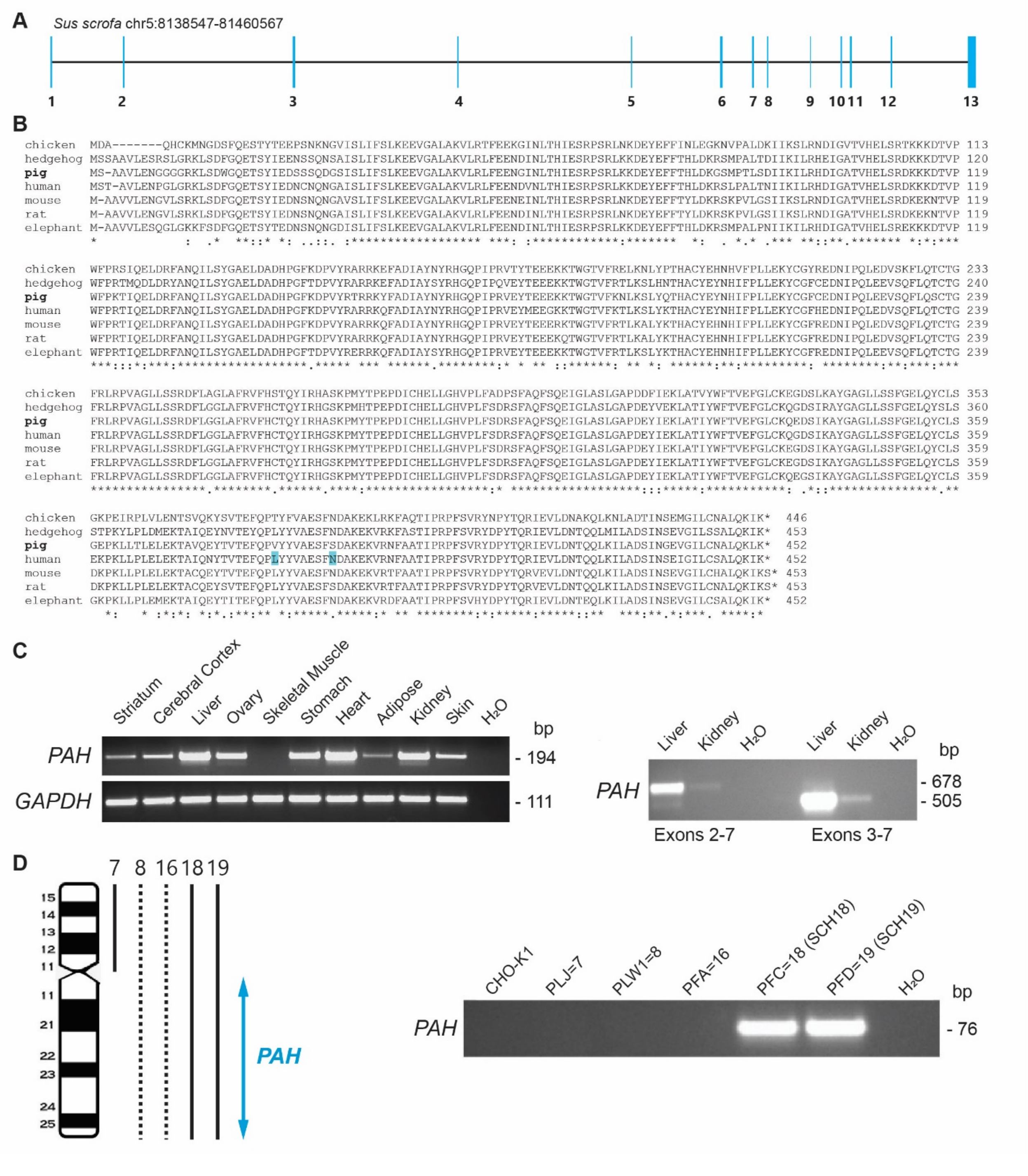

Supplemental Figure S1. Characterization of the *PAH* gene in *Sus scrofa*. (**A**) Gene structure of porcine *PAH* drawn to scale with its 13 exons in cyan numbered below. This gene structure is

conserved in other mammalian species including human. (**B**) The pig phenylalanine-4-hydroxylase (PAH) polypeptide sequence is highly conserved with other vertebrate species. Variants in pig breeds are highlighted in blue shade (V385 or L385 and S393 or N393). (**C**) Expression of *PAH* in Göttingen minipig tissues. (left) RT-PCR expression profile of *PAH* exon 2-3 across multiple tissues shows highest levels in liver, heart and kidney; (right) RT-PCR detection of *PAH* exon 2-7 and exon 3-7 confirms high expression in liver and moderate expression in kidney. (**D**) Genomic PCR in a subpanel of five pig-hamster somatic cell hybrid (SCH) lines indicates the mapping (blue arrow) of *PAH* to the long arm of *Sus scrofa* chromosome 5 (SSC5q). Black vertical bars indicate the SSC5 segment present in each SCH line, with dashed lines indicating the absence of SSC5 in those SCH lines. CHO-K1 is a Chinese hamster ovary control cell line.

Supplemental Figure S2. ***PAH*** **open reading frame and translated protein products in (A) wild-type, (B) exon 6 deletion, (C) and exon 6-7 deletion mutants.**

A. **Wild-Type:**

```
   1 ATGTCAGCGG CGGTCCTGGA GAACGGAGGC GGGGGCCGCA AACTCAGCGA CTGGGGACAG
     M  S  A  A  V  L  E  N  G  G  G  G  R  K  L  S  D  W  G  Q

  61 GAAACAAGCT ATATTGAAGA CAGCTCCAGT CAAGATGGTT CCATATCACT GATCTTCTCA
     E  T  S  Y  I  E  D  S  S  S  Q  D  G  S  I  S  L  I  F  S

 121 CTCAAAGAAG AAGTTGGTGC GCTGGCCAAA GTCTTGCGTT TATTTGAGGA GAATGGTATA
     L  K  E  E  V  G  A  L  A  K  V  L  R  L  F  E  E  N  G  I

 181 AACCTGACTC ACATTGAATC CCGACCTTCG CGTTTAAAGA AAGATGAGTA TGAGTTTTTC
     N  L  T  H  I  E  S  R  P  S  R  L  K  K  D  E  Y  E  F  F

 241 ACCCACCTGG ATAAAGGCAG CATGCCTACT CTGTCAGACA TCATCAAGAT CTTGAGACAT
     T  H  L  D  K  G  S  M  P  T  L  S  D  I  I  K  I  L  R  H

 301 GACATTGGCG CCACCGTGCA CGAGCTCTCC CGGGACAAGA AGAAAGACAC AGTGCCCTGG
     D  I  G  A  T  V  H  E  L  S  R  D  K  K  K  D  T  V  P  W

 361 TTCCCCAAAA CCATTCAAGA GCTGGACAGA TTTGCCAATC AGATTCTCAG CTACGGAGCG
     F  P  K  T  I  Q  E  L  D  R  F  A  N  Q  I  L  S  Y  G  A

 421 GAACTGGATG CAGATCACCC TGGTTTTAAA GATCCTGTGT ACCGAACAAG ACGGAAGTAC
     E  L  D  A  D  H  P  G  F  K  D  P  V  Y  R  T  R  R  K  Y

 481 TTTGCTGACA TTGCCTACAA CTATCGACAT GGGCAGCCCA TCCCCCGAGT GGAGTACACA
     F  A  D  I  A  Y  N  Y  R  H  G  Q  P  I  P  R  V  E  Y  T

 541 GAGGAAGAGA AGAAAACGTG GGGGACGGTG TTCAAGAACC TGAAGTCCTT GTATCAAACC
     E  E  E  K  K  T  W  G  T  V  F  K  N  L  K  S  L  Y  Q  T

 601 CACGCCTGCT ACGAGTACAA TCACATTTTC CCACTTCTGG AAAAGTACTG TGGTTTCTGC
     H  A  C  Y  E  Y  N  H  I  F  P  L  L  E  K  Y  C  G  F  C

 661 GAAGATAATA TTCCCCAGCT GGAAGAAGTT TCTCAGTTTC TGCAGTCTTG CACCGGTTTC
     E  D  N  I  P  Q  L  E  E  V  S  Q  F  L  Q  S  C  T  G  F

 721 CGCCTCCGAC CTGTGGCCGG CCTGCTTTCG TCTCGGGATT TCCTGGGTGG CCTGGCTTTC
     R  L  R  P  V  A  G  L  L  S  S  R  D  F  L  G  G  L  A  F

 781 CGAGTCTTCC ACTGCACTCA GTACATCAGA CACGCGTCCA AGCCCATGTA TACACCTGAA
     R  V  F  H  C  T  Q  Y  I  R  H  A  S  K  P  M  Y  T  P  E

 841 CCTGACATCT GCCATGAGCT GTTGGGACAT GTACCCTTGT TTTCAGATCG CAGCTTTGCC
     P  D  I  C  H  E  L  L  G  H  V  P  L  F  S  D  R  S  F  A

 901 CAGTTTTCCC AGGAAATCGG CCTCGCCTCT GTGGGGGCAC CTGACGAGTA TATTGAGAAA
     Q  F  S  Q  E  I  G  L  A  S  V  G  A  P  D  E  Y  I  E  K

 961 CTTGCAACAA TTTACTGGTT TACTGTGGAG TTTGGGCTCT GCAAGCAAGA AGACTCCATA
     L  A  T  I  Y  W  F  T  V  E  F  G  L  C  K  Q  E  D  S  I

1021 AAGGCATATG GTGCTGGGCT CCTGTCATCC TTTGGTGAAT TACAGTATTG CTTGTCAGGT
     K  A  Y  G  A  G  L  L  S  S  F  G  E  L  Q  Y  C  L  S  G

1081 GAGCCAAAGC TCCTCACTCT GGAACTGGAG AAGACAGCTG TCCAGGAGTA CACAGTCACG
     E  P  K  L  L  T  L  E  L  E  K  T  A  V  Q  E  Y  T  V  T

1141 GAGTTCCAGC CCGTGTACTA CGTGGCCGAG AGTTTCAATG ATGCCAAGGA GAAAGTGAGG
     E  F  Q  P  V  Y  Y  V  A  E  S  F  N  D  A  K  E  K  V  R

1201 AACTTTGCTG CCACAATTCC TCGGCCCTTC TCAGTTCGTT ATGACCCATA CACCCAAAGG
     N  F  A  A  T  I  P  R  P  F  S  V  R  Y  D  P  Y  T  Q  R

1261 ATTGAGGTCT TGGACAACAC CCAGCAGCTT AAGATTCTGG CTGACTCCAT CAACGGTGAA
     I  E  V  L  D  N  T  Q  Q  L  K  I  L  A  D  S  I  N  G  E

1321 GTTGGAATCC TTTGCAATGC CCTCCAGAAA TTAAAGTGA
     V  G  I  L  C  N  A  L  Q  K  L  K  *
```

Supplemental Figure S2 (cont.)

B. Exon 6 Deletion:

```
  1 ATGTCAGCGG CGGTCCTGGA GAACGGAGGC GGGGGCCGCA AACTCAGCGA CTGGGGACAG
     M  S  A  A  V  L  E  N  G  G  G  G  R  K  L  S  D  W  G  Q

 61 GAAACAAGCT ATATTGAAGA CAGCTCCAGT CAAGATGGTT CCATATCACT GATCTTCTCA
     E  T  S  Y  I  E  D  S  S  S  Q  D  G  S  I  S  L  I  F  S

121 CTCAAAGAAG AAGTTGGTGC GCTGGCCAAA GTCTTGCGTT TATTTGAGGA GAATGGTATA
     L  K  E  E  V  G  A  L  A  K  V  L  R  L  F  E  E  N  G  I

181 AACCTGACTC ACATTGAATC CCGACCTTCG CGTTTAAAGA AAGATGAGTA TGAGTTTTTC
     N  L  T  H  I  E  S  R  P  S  R  L  K  K  D  E  Y  E  F  F

241 ACCCACCTGG ATAAAGGCAG CATGCCTACT CTGTCAGACA TCATCAAGAT CTTGAGACAT
     T  H  L  D  K  G  S  M  P  T  L  S  D  I  I  K  I  L  R  H

301 GACATTGGCG CCACCGTGCA CGAGCTCTCC CGGGACAAGA AGAAAGACAC AGTGCCCTGG
     D  I  G  A  T  V  H  E  L  S  R  D  K  K  K  D  T  V  P  W

361 TTCCCCAAAA CCATTCAAGA GCTGGACAGA TTTGCCAATC AGATTCTCAG CTACGGAGCG
     F  P  K  T  I  Q  E  L  D  R  F  A  N  Q  I  L  S  Y  G  A

421 GAACTGGATG CAGATCACCC TGGTTTTAAA GATCCTGTGT ACCGAACAAG ACGGAAGTAC
     E  L  D  A  D  H  P  G  F  K  D  P  V  Y  R  T  R  R  K  Y

481 TTTGCTGACA TTGCCTACAA CTATCGACAC TTGCACCGGT TTCCGCCTCC GACCTGTGGC
     F  A  D  I  A  Y  N  Y  R  H  L  H  R  F  P  P  P  T  C  G

541 CGGCCTGCTT TCGTCTCGGG ATTTCCTGGG TGGCCTGGCT TTCCGAGTCT TCCACTGCAC
     R  P  A  F  V  S  G  F  P  G  W  P  G  F  P  S  L  P  L  H

601 TCAGTACATC AGACACGCGT CCAAGCCCAT GTATACACCT GA
     S  V  H  Q  T  R  V  Q  A  H  V  Y  T  *
```

C. Exon 6-7 Deletion:

```
  1 ATGTCAGCGG CGGTCCTGGA GAACGGAGGC GGGGGCCGCA AACTCAGCGA CTGGGGACAG
     M  S  A  A  V  L  E  N  G  G  G  G  R  K  L  S  D  W  G  Q

 61 GAAACAAGCT ATATTGAAGA CAGCTCCAGT CAAGATGGTT CCATATCACT GATCTTCTCA
     E  T  S  Y  I  E  D  S  S  S  Q  D  G  S  I  S  L  I  F  S

121 CTCAAAGAAG AAGTTGGTGC GCTGGCCAAA GTCTTGCGTT TATTTGAGGA GAATGGTATA
     L  K  E  E  V  G  A  L  A  K  V  L  R  L  F  E  E  N  G  I

181 AACCTGACTC ACATTGAATC CCGACCTTCG CGTTTAAAGA AAGATGAGTA TGAGTTTTTC
     N  L  T  H  I  E  S  R  P  S  R  L  K  K  D  E  Y  E  F  F

241 ACCCACCTGG ATAAAGGCAG CATGCCTACT CTGTCAGACA TCATCAAGAT CTTGAGACAT
     T  H  L  D  K  G  S  M  P  T  L  S  D  I  I  K  I  L  R  H

301 GACATTGGCG CCACCGTGCA CGAGCTCTCC CGGGACAAGA AGAAAGACAC AGTGCCCTGG
     D  I  G  A  T  V  H  E  L  S  R  D  K  K  K  D  T  V  P  W

361 TTCCCCAAAA CCATTCAAGA GCTGGACAGA TTTGCCAATC AGATTCTCAG CTACGGAGCG
     F  P  K  T  I  Q  E  L  D  R  F  A  N  Q  I  L  S  Y  G  A

421 GAACTGGATG CAGATCACCC TGGTTTTAAA GATCCTGTGT ACCGAACAAG ACGGAAGTAC
     E  L  D  A  D  H  P  G  F  K  D  P  V  Y  R  T  R  R  K  Y

481 TTTGCTGACA TTGCCTACAA CTATCGACAT GACATCTGCC ATGAGCTGTT GGGACATGTA
     F  A  D  I  A  Y  N  Y  R  H  D  I  C  H  E  L  L  G  H  V

541 CCCTTGTTTT CAGATCGCAG CTTTGCCCAG TTTTCCCAGG AAATCGGCCT CGCCTCTGTG
     P  L  F  S  D  R  S  F  A  Q  F  S  Q  E  I  G  L  A  S  V

601 GGGGCACCTG ACGAGTATAT TGAGAAACTT GCAACAATTT ACTGGTTTAC TGTGGAGTTT
     G  A  P  D  E  Y  I  E  K  L  A  T  I  Y  W  F  T  V  E  F
```

Supplemental Figure S2 (cont.)

```
 660 GGGCTCTGCA AGCAAGAAGA CTCCATAAAG GCATATGGTG CTGGGCTCCT GTCATCCTTT
     G  L  C  K   Q  E  D   S  I  K   A  Y  G  A   G  L  L   S  S  F

 720 GGTGAATTAC AGTATTGCTT GTCAGGTGAG CCAAAGCTCC TCACTCTGGA ACTGGAGAAG
     G  E  L  Q   Y  C  L   S  G  E   P  K  L  L   T  L  E   L  E  K

 781 ACAGCTGTCC AGGAGTACAC AGTCACGGAG TTCCAGCCCG TGTACTACGT GGCCGAGAGT
     T  A  V  Q   E  Y  T   V  T  E   F  Q  P  V   Y  Y  V   A  E  S

 841 TTCAATGATG CCAAGGAGAA AGTGAGGAAC TTTGCTGCCA CAATTCCTCG GCCCTTCTCA
     F  N  D  A   K  E  K   V  R  N   F  A  A  T   I  P  R   P  F  S

 901 GTTCGTTATG ACCCATACAC CCAAAGGATT GAGGTCTTGG ACAACACCCA GCAGCTTAAG
     V  R  Y  D   P  Y  T   Q  R  I   E  V  L  D   N  T  Q   Q  L  K

 961 ATTCTGGCTG ACTCCATCAA CGGTGAAGTT GGAATCCTTT GCAATGCCCTC CAGAAATTA
     I  L  A  D   S  I  N   G  E  V   G  I  L  C   N  A  L   Q  K  L

1021 AAGTGA
     K  *
```

Supplemental Figure S2. ***PAH*** **open reading frame and translated protein products in (A) wild-type, (B) exon 6 deletion, (C) and exon 6-7 deletion mutants.** Translation initiation and termination codons are underlined. With the exon 6 deletion there would be early termination codon usage due to a frameshift whereas the exon 6-7 deletion is in-frame and translation would lead to a shortened polypeptide sequence utilizing the same terminator as for the wild-type *PAH* transcripts. The open reading frame (ORF) nucleotide position is given on the left and the amino acid single letter code is written below with an asterisk (*) for stop codons. A red arrowhead indicates deletion of exon 6 and resulting out-of-frame translation and early termination (red amino acid sequence), while a green arrowhead indicates the site of exon 6-7 deletion that results in ablation of 111 amino acid residues in the PAH catalytic domain.

Supplemental Figure S3. Characterization of *PAH* gene-edited recombinant deletion and inversion breakpoints induced in SCH lines.

A

```
                      Intron 5                         5-1 6-1                         Intron 6
Del5    CAAGATCTGGGGGCAGGTCCAACACATGTACTTTAACAAGCTGCCCGTAG------GAAGACGGATTCTCCCAGGAAAACCAGAATTTCACTGTTTCACTG
Del7    CAAGATCTGGGGGCAGGTCCAACACATGTACTTTAACAAGCTGCCCGTAG------GAAGACGGATTCTCCCAGGAAAACCAGAATTTCACTGTTTCACTG
Del2    CAAGATCTGGGGGCAGGTCCAACACATGTACTTTAACAAGCTGCCCGTAGATGCAGGAAGACGGATTCTCCCAGGAAAACCAGAATTTCACTGTTTCACTG
Del1    CAAGATCTGGGGGCAGGTCCAACACATGTACTTTAACAAGCTGCCCGTAG-TGCAGGAAGACGGATTCTCCCAGGAAAACCAGAATTTCACTGTTTCACTG
Del9    CAAGATCTGGGGGCAGGTCCAACACATGTACTTTAACAAGCTGCCCGTAG-TGCAGGAAGACGGATTCTCCCAGGAAAACCAGAATTTCACTGTTTCACTG
Del4    CAAGATCTGGGGGCAGGTCCAACACATGTACTTTAACAAGCTGCCCGTAG-TGCAGGAAGACGGATTCTCCCAGGAAAACCAGAATTTCACTGTTTCACTG
Del3    CAAGATCTGGGGGCAGGTCCAACACATGTACTTTAACAAGCTGCCCGTAG-TGCGGGAAGACGGATTCTCCCAGGAAAACCAGAATTTCACTGTTTCACTG
Duroc   CAAGATCTGGGGGCAGGTCCAACACATGTACTTTAACAAGCTGCCCGTAG/TGCAGGAAGACGGATTCTCCCAGGAAAACCAGAATTTCACTGTTTCACTG
```

B

```
                      Intron 5                5-2          6-1              Intron 6
Del17   CTACTTGACATATTTATGTTACAGGCCCTTGAAGGTAAGTGAATT------------AAGACGGATTCTCCCAGGAAAACCAGAATTTCACTGTTTCACTG
Del1    CTACTTGACATATTTATGTTACAGGCCCTTGAAGGTAAGTGAATTCCCA-T-GCAGGAAGACGGATTCTCCCAGGAAAACCAGAATTTCACTGTTTCACTG
Del3    CTACTTGACATATTTATGTTACAGGCCCTTGAAGGTAAGTGAATTCCCA-T-GCAGGAAGACGGATTCTCCCAGGAAAACCAGAATTTCACTGTTTCACTG
Del4    CTACTTGACATATTTATGTTACAGGCCCTTGAAGGTAAGTGAATTCCCA-T-GCAGGAAGACGGATTCTCCCAGGAAAACCAGAATTTCACTGTTTCACTG
Del18   CTACTTGACATATTTATGTTACAGGCCCTTGAAGGTAG-------------TGCAGGAAGACGGATTCTCCCAGGAAAACCAGAATTTCACTGTTTCACTG
Del6    CTACTTGACATATTTATGT--------------------------------------------------------------------TCACTGTTTCACTG
Del7    CTACTTGACATATTTATGT--------------------------------------------------------------------TCACTGTTTCACTG
Del8    CTACTTGACATATTTATGT--------------------------------------------------------------------TCACTGTTTCACTG
Del14   CTACTTGACATATTTATGTTACAGGCCCTTGGAGGT---------------------AAGACGGATTCTCCCAGGAAAACCAGAATTTCACTGTTTCACTG
Del9    CTACTTGACATATTTATGTTACAGGCCCTTGAAGGTAAGTGAATTCCCAT-TGCAGGAAGACGGATTCTCCCAGGAAAACCAGAATTTCACTGTTTCACTG
Del11   CTACTTGACATATTTATGTTACAGGCCCTTGAAGGTAAGTGAATTCCCAT-TGCAGGAAGACGGATTCTCCCAGGAAAACCAGAATTTCACTGTTTCACTG
Del12   CTACTTGACATATTTATGTTACAGGCCCTTGAAGGTAAGTGAATTCCCAT-TGCAGGAAGACGGATTCTCCCAGGAAAACCAGAATTTCACTGTTTCACTG
Del13   CTACTTGACATATTTATGTTACAGGCCCTTGAAGGTAAGTGAATTCCCAT-TGCAGGAAGACGGATTCTCCCAGGAAAACCAGAATTTCACTGTTTCACTG
Del2    CTACTTGACATATTTATGTTACAGGCCCTTGAAGGTAAGTGAATTCCCAT-TGCAGGAAGACGGATTCTCCCAGGAAAACCAGAATTTCACTGTTTCACTG
Del5    CTACTTGACATATTTATGTTACAGGCCCTTGAAGGTAAGTGAATTCCCAT-TGCAGGAAGACGGATTCTCCCAGGAAAACCAGAATTTCACTGTTTCACTG
Duroc   CTACTTGACATATTTATGTTACAGGCCCTTGAAGGTAAGTGAATTCCCAT/TGCAGGAAGACGGATTCTCCCAGGAAAACCAGAATTTCACTGTTTCACTG
```

C

```
                          Intron 5                     5-1 6-2                         Intron 6
Del5    CAAGATCTGGGGGCAGGTCCAACACATGTACTTTAACAAGCTGCCCGTAGGAAAAGGCATTAGCTGTGGATTTTGCCATTTCCATGATCCTGAAAAGTACT
Del4    CAAGATCTGGGGGCAGGTCCAACACATGTACTTTAACAAGCTGCCCGTAG-AAAAGGCATTAGCTGTGGATTTTGCCACTTCCATGATCCTGAAAAGTACT
Del1    CAAGATCTGGGGGCAGGTCCAACACATGTACTTTAACAAGCTGCCCGTAG-AAAAGGCATTAGCTGTGGATTTTGCCATTTCCATGATCCTGAAAAGTACT
Del2    CAAGATCTGGGGGCAGGTCCAACACATGTACTTTAACAAGCTGCCCGTAG-AAAAGGCATTAGCTGTGGATTTTGCCATTTCCATGATCCTGAAAAGTACT
Del3    CAAGATCTGGGGGCAGGTCCAACACATGTACTTTAACAAGCTGCCCGTAG-AAAAGGCATTAGCTGTGGATTTTGCCATTTCCATGATCCTGAAAAGTACT
Duroc   CAAGATCTGGGGGCAGGTCCAACACATGTACTTTAACAAGCTGCCCGTAG/AAAAGGCATTAGCTGTGGATTTTGCCATTTCCATGATCCTGAAAAGTACT
```

D

```
                      Intron 5                   5-2          6-2            Intron 6
Del6    GCTACTTGACATATTTATGTTACAGGCCCTTGAAGGTAAGTGAATTCCC-------AGGCATTAGCTGTGGATTTTGCCATTTCCATGATCCTGAAAAGTAC
Del16   GCTACTTGACATATTTATGTTACAGGCCTTGAAGGTAAGTGGATTT-----------------------------TGCCATTTCCATGATCCTGAAAAGTAC
Del11   GCTACTTGACATATTTATGTTACAGGCCCTTGAAGGTAAGTG------------AAAGGCATTAGCTGTGGATTTTGCCATTTCCATGATCCTGAAAAGTAC
Del3    GCTACTTGACATATTTATGTTACAGGCCCTTGAAGGTAAGTGAATTCCCAT-GAAAAGGCATTAGCTGTGGATTTTGCCATTTCCATGATCCTGAAAAGTAC
Del8    GCTACTTGACATATTTATGTTACAGGCCCTTGAAGGTAAGTGAATTCCCAT-GAAAAGGCATTAGCTGTGGATTTTGCCATTTCCATGATCCTGAAAAGTAC
Del7    GCTACTTGACATATTTATGTTACAGGCCCTTGAAGGTAAGTGAATTCCCATA-AAAAGGCATTAGCTGTGGATTTTGCCATTTCCATGATCCTGAAAAGTAC
Del10   GCTACTTGACATATTTATGTTACAGGCCCTTGAAGGTAAGTGAATTCCCAT-GAAAAGGCATTAGCTGTGGATTTTGCCATTTCCATGATCCTGAAAAGTAC
Del13   GCTACTTGACATATTTATGTTACAGGCCCTTGAAGGTAT--------------AAAAGGCATTAGCTGTGGATTTTGCCATTTCCATGATCCTGAAAAGTAC
Del9    GCTACTTGACATATTTATGTTACAGGCCCTTGAAGGTAAGTGAATTCCCAT--AAAAGGCATTAGCTGTGGATTTTGCCATTTCCATGATCCTGAAAAGTAC
Del5    GCTACTTGACATATTTATGTTACAGGCCCTTGAAGGTAAGTGAATTCCCAT--AAAAGGCATTAGCTGTGGATTTTGCCATTTCCATGATCCTGAAAAGTAC
Del12   GCTACTTGACATATTTATGTTACAGGCCCTTGAAGGTAAGTGAATTCCCAT--AAAAGGCATTAGCTGTGGATTTTGCCATTTCCATGATCCTGAAAAGTAC
Del15   GCTACTTGACATATTTATGTTACAGGCCCTTGAAGGTAAGTGAATTCCCAT--AAAAGGCATTAGCTGTGGATTTTGCCATTTCCATGATCCTGAAAAGTAC
Del2    GCTACTTGACATATTTATGTTACAGGCCCTTGAAGGTAAGTGAATTCCCAT-----------TAGCTGTGGATTTTGCCATTTCCATGATCCTGAAAAGTAC
Del14   GCTACTTGACATATTTATGTTACAGGCCCTTGAAGGTAAGTG----------GAAAAGGCATTAGCTGTGGATTTTGCCATTTCCATGATCCTGAAAAGTAC
Del1    GCTACTTGACATATTTATGTTACAGGCCCTTGAAGGTAAGTGAATTCCCATGGAAAAGGCATTAGCTGTGGATTTTGCCATTTCCATGATCCTGAAAAGTAC
Del4    GCTACTTGACATATTTATGTTACAGGCCCTTGAAGGTAAGTGAATTCCCATGGAAAAGGCATTAGCTGTGGATTTTGCCATTTCCATGATCCTGAAAAGTAC
Duroc   GCTACTTGACATATTTATGTTACAGGCCCTTGAAGGTAAGTGAATTCCCAT//AAAAGGCATTAGCTGTGGATTTTGCCATTTCCATGATCCTGAAAAGTAC
```

Supplemental Figure S3 (cont.)

E

```
                    Intron 5                                5-1       6-1        Intron 6 RevComp
pInv3  ACAGGTTCAAGATCTGGGGGCAGGTCCAACACATGTACTTTAACAAGCTGCCCGTAG-TCAGCACAAACGCACTCACGCGCACCTTGGCCTCAATTTACTT
pInv2  ACAGGTTCAAGATCTGGGGGCAGGTCCAACACATGTACTTTAACAAGCTGCCCGTAG-TCAGCACAAACGCACTCACGCGCACCTTGGCCTCAATTTACTT
pInv5  ACAGGTTCAAGATCTGGGGGCAGGTCCAACACATGTACTTTAACAAGCTGCCCGTAG-TCAGCACAAACGCACTCACGCGCACCTTGGCCTCAATTTACTT
pInv4  ACAGGTTCAAGATCTGGGGGCAGGTCCAACACATGTACTTTAACAAGCTGCCCGTAG-TCAGCACAAACGCACTCACGCGCACCTTGGCCTCAATTTACTT
Duroc  ACAGGTTCAAGATCTGGGGGCAGGTCCAACACATGTACTTTAACAAGCTGCCCGTAG/TCAGCACAAACGCACTCACGCGCACCTTGGCCTCAATTTACTT
```

F

```
                    Intron 5                           5-2            6-1        Intron 6 RevComp
pInv4  ATACCTGCTACTTGACATATTTATGTTACAGGCCCTTGAAGGTAAGTGAATTCCCAT-TCAGCACNAACGCNCTCACGCGCACCTTGGCCTCNAATTTACT
pInv3  ATACCTGCTACTTGACATATTTATGTTACAGGCCCTTGAAGGTAAGTGAATTCCCAT-TCAGCACAAACGCACTCACGCGCACCTTGGCCTCAATTTACTT
pInv1  ATACCTGCTACTTGACATATTTATGTTACAGGCCCTTGAAGGTAAGTGAATTCCCAT-TCAGCACAAACGCACTCACGCGCACCTTGGCCTCAATTTACTT
pInv5  ATACCTGCTACTTGACATATTTATGTTACAGGCCCTTGAAGGTAAGTGAATTCCCAT-TCAGCACAAACGCACTCACGCGCACCTTGGCCTCAATTTACTT
pInv2  ATACCTGCTACTTGACATATTTATGTTACAGGCCCTTGAAGGTAAGTGAATTCCCAT-TCAGCACAAACGCACTCACGCGCACCTTGGCCTCAATTTACTT
Duroc  ATACCTGCTACTTGACATATTTATGTTACAGGCCCTTGAAGGTAAGTGAATTCCCAT/TCAGCACAAACGCACTCACGCGCACCTTGGCCTCAATTTACTT
```

G

```
                        Intron 5                          5-1    6-2            Intron 6 RevComp
pInv1     CAAGATCTGGGGGCAGGTCCAACACATGTACTTTAACAAGCTGCCCGTAG-CTTGGATGGCGGTAGACTAACCGTGGGGGCCTGAGGGGAGGCAGAGCCCA
pInv3     CAAGATCTGGGGGCAGGTCCAACACATGTACTTTAACAAGCTGCCCGTAG-CTTGGATGGCGGTAGACTAACCGTGGGGGCCTGAGGGGAGGCAGAGCCCA
pInv4     CAAGATCTGGGGGCAGGTCCAACACATGTACTTTAACAAGCTGCCCGTAG-CTTGGATGGCGGTAGACTAACCGTGGGGGCCTGAGGGGAGGCAGAGCCCA
pInv2     CAAGATCTGGGGGCAGGTCCAACACATGTACTTTAACAAGCTGCCCGTAG-CTTGGATGGCGGTAGACTAACCGTGGGGGCCTGAGGGGAGGCAGAGCCCA
Duroc     CAAGATCTGGGGGCAGGTCCAACACATGTACTTTAACAAGCTGCCCGTAG/CTTGGATGGCGGTAGACTAACCGTGGGGGCCTGAGGGGAGGCAGAGCCCA
```

H

```
                 Intron 5                      5-2         6-2           Intron 6 RevComp
pInv1  CTACTTGACATATTTATGTTACAGGCCCTTGAAGGTAAGTGAATTCCCAT-CTTGGATGGCGGTAGACTAACCGTGGGGGCCTGAGGGGAGGCAGAGCCCA
pInv2  CTACTTGACATATTTATGTTACAGGCCCTTGAAGGTAAGTGAATTCCCAT-CTTGGATGGCGGTAGACTAACCGTGGGGGCCTGAGGGGAGGCAGAGCCCA
pInv4  CTACTTGACATATTTATGTTACAGGCCCTTGAAGGTAAGTGAATTCCCAT-CTTGGATGGCGGTAGACTAACCGTGGGGGCCTGAGGGGAGGCAGAGCCCA
pInv3  CTACTTGACATATTTATGTTACAGGCCCTTGAAGGTAAGTGAATTCCCAT--TTGGATGGCGGTAGACTAACCGTGGGGGCCTGAGGGGAGGCAGAGCCCA
Duroc  CTACTTGACATATTTATGTTACAGGCCCTTGAAGGTAAGTGAATTCCCAT/CTTGGATGGCGGTAGACTAACCGTGGGGGCCTGAGGGGAGGCAGAGCCCA
```

I

```
               Intron 5 RevComp             5-1           6-1             Intron 6
dInv2  CTAAGATCCAGATCCATTTACCTGGCATCTCCGGCTATGGCAGAA---------------GACGGATTCTCCCAGGAAAACCAGAATTTCACTGTTTCACTG
dInv3  CTAAGATCCAGATCCATTTACCTGGCATCTCCGGCTATGGCAGAACAGAA--TGCAGGAAGACGGATTCTCCCAGGAAAACCAGAATTTCACTGTTTCACTG
dInv5  CTAAGATCCAGATCCATTTACCTGGCATCTCCGGCTATGGCAGAACAAAATATGCAGGAAGACGGATTCTCCCAGGAAAACCAGAATTTCACTGTTTCACTG
dInv4  CTAAGATCCAGATCCATTTACCTGGCATCTCCGGCTATGGCAGAACAAAA--TGCAGGAAGACGGATTCTCCCAGGAAAACCAGAATTTCACTGTTTCACTG
dInv1  CTAAGATCCAGATCCATTTACCTGGCATCTCCGGCTATGGCAGAACAAAATATGCAGGAAGACGGATTCTCCCAGGAAAACCAGAATTTCACTGTTTCACTG
Duroc  CTAAGATCCAGATCCATTTACCTGGCATCTCCGGCTATGGCAGAACAAAA//TGCAGGAAGACGGATTCTCCCAGGAAAACCAGAATTTCACTGTTTCACTG
```

J

```
                        Intron 5 RevComp             5-2 6-1             Intron 6
dInv4  AACTCCTCACTTGGCTTTTCTGCTCTCCTATTTCCTATTCTTTACCCAGT--GCAGGAAGACGGATTCTCCCAGGAAAACCAGAATTTCACTGTTTCACTG
dInv3  AACTCCTCACTTGGCTTTTCTGCTCTCCTATTTCCTATTCTTTACCCAGT-TGCAGGAAGACGGATTCTCCCAGGAAAACCAGAATTTCACTGTTTCACTG
dInv9  AACTCCTCACTTGGCTTTTCTGCTCTCCTATTTCCTATTCTTTACCCAGT-TGCAGGAAGACGGATTCTCCCAGGAAAACCAGAATTTCACTGTTTCACTG
dInv5  AACTCCTCACTTGGCTTTTCTGCTCTCCTATTTCCTATTCTTTACCCAGT-TGCAGGAAGACGGATTCTCCCAGGAAGACCAGAATTTCACTGTTTCACTG
dInv1  AACTCCTCACTTGGCTTTTCTGCTCTCCTATTTCCTATTCTTTACCCAGT-TGCAGGAAGACGGATTCTCCCAGGAAAACCAGAATTTCACTGTTTCACTG
dInv2  AACTCCTCACTTGGCTTTTCTGCTCTCCTATTTCCTATTCTTTACCCAGT-TGCAGGAAGACGGATTCTCCCAGGAAAACCAGAATTTCACTGTTTCACTG
dInv7  AACTCCTCACTTGGCTTTTCTGCTCTCCTATTTCCTATTCTTTACCCAGT-TGCAGGAAGACGGATTCTCCCAGGAAAACCAGAATTTCACTGTTTCACTG
dInv8  AACTCCTCACTTGGCTTTTCTGCTCTCCTATTTCCTATTCTTTACCCAGT-TGCAGGAAGACGGATTCTCCCAGGAAAACCAGAATTTCACTGTTTCACTG
dInv6  AACTCCTCACTTGGCTTTTCTGCTCTCCTATTTCCTATCCTTTACCCAGT-TGCAGGAAGACGGATTCTCCCAGGAAAACCAGAATTTCACTGTTTCACTG
Duroc  AACTCCTCACTTGGCTTTTCTGCTCTCCTATTTCCTATTCTTTACCCAGT/TGCAGGAAGACGGATTCTCCCAGGAAAACCAGAATTTCACTGTTTCACTG
```

K

```
                        Intron 5 RevComp         5-1       6-2             Intron 6
dInv3  TCTAANATCCANATNCATTTACCTGGCATCTCCGGCTATGGCANAACAAAA--AAAGGCATTANCTGTGGATTTTGCCATTTCCATGATCCTGAAAAGTAC
dInv1  TCTAAGATCCAGATCCATTTACCTGGCATCTCCGGCTATGGCAGAACA---GAAAAGGCATTAGCTGTGGATTTTGCCATTTCCATGATCCTGAAAAGTAC
dInv2  TCTAAGATCCAGATCCATTTACCTGGCATCTCCGGCTATGGCAGAACAAAAGAAAAGGCATTAGCTGTGGATTTTGCCATTTCCATGATCCTGAAAAGTAC
Duroc  TCTAAGATCCAGATCCATTTACCTGGCATCTCCGGCTATGGCAGAACAAAA/AAAAGGCATTAGCTGTGGATTTTGCCATTTCCATGATCCTGAAAAGTAC
```

Supplemental Figure S3 (cont.)

L

```
                         Intron 5 RevComp                         5-2   6-2                Intron 6
dInv10  AACTCCTCGCTTGGCTTTTCTGCTCTCCTATTTCCTATTCTTTACCCAGT--GAAAAGGCATTAGCTGTGGATTTTGCCATTTCCATGATCCTGAAAAGTAC
dInv3   AACTCCTCACTTGGCTTTTCTGCTCTCCTATTTCCTATTCTTTACCCAGT-AGAAAAGGCATTAGCTGTGGATTTTGCCATTTCCATGATCCTGAAAAGTAC
dInv9   AACTCCTCACTTGGCTTTTCTGCTCTCCTATTTCCTATTCTTTACCCAGTA---------ATTAGCTGTGGATTTTGCCATTTCCATGATCCTGAAAAGTAC
dInv7   AACTCCTCACTTGGCTTTTCTGCTCTCCTATTTCCTATTCTTTACCCAGT-----AAGGCATTAGCTGTGGATTTTGCCATTTCCATGATCCTGAAAAGTAC
dInv11  AACTCCTCACTTGGCTTTTCTGCTCTCCTATTTCCTATTCTTCACCCAGTAT-AAAAGGCATTAGCTGTGGATTTTGCCATTTCCATGATCCTGAAAAGTAC
dInv8   AACTCCTCACTTGGCTTTTCTGCTCTCCTATTTCCTATTCTTTACCCAGTTT-AAAAGGCATTAGCTGTGGATTTTGCCATTTCCATGATCCTGAAAAGTAC
dInv6   AACTCCTCACTTGGCTTTTCTGCTCTCCTATTTCCTATTCTTTACCCAGT-AGAAAAGGCATTAGCTGTGGATTTTGCCATTTCCATGATCCTGAAAAGTAC
dInv2   AACTCCTCACTTGGCTTTTCTGCTCTCCTATTTCCTATTCTTTACCCAGT-AGAAAAGGCATTAGCTGTGGATTTTGCCATTTCCATGATCCTGAAAAGTAC
dInv1   AACTCCTCACTTGGCTTTTCTGCTCTCCTATTTCCTATTCTTTACCCAGT-AGAAAAGGCATTAGCTGTGGATTTTGCCATTTCCATGATCCTGAAAAGTAC
dInv5   AACTCCTCACTTGGCTTTTCTGCTCTCCTATTTCCTATTCTTTACCCAGT-AGAAAAGGCATTAGCTGTGGATTTTGCCATTTCCATGATCCTGAAAAGTAC
dInv4   AACTCCTCACTTGGCTTTTCTGCTCTCCTATTTCCTATTCTTTACCCAGTA-GGAAAGGCATTAGCTGTGGATTTTGCCATTTCCATGATCCTGAAAAGTAC
Duroc   AACTCCTCACTTGGCTTTTCTGCTCTCCTATTTCCTATTCTTTACCCAGT///AAAAGGCATTAGCTGTGGATTTTGCCATTTCCATGATCCTGAAAAGTAC
```

Supplemental Figure S3. Characterization of *PAH* gene-edited recombinant deletion and inversion breakpoints induced in the SCH cell lines. Multiple sequence alignments of recombinant breakpoints derived from transfected SCH18 and SCH19 lines sequenced after PCR amplification and topo-TA cloning compared to reference Duroc genomic sequence, with expected breakpoints based on canonical DSB at each PAM -3 position. (**A-D**) Intron 5-6 deletion-breakpoints (see **Fig. 1C**, top panel) generated by CRISPR/Cas9 sgRNA pairs: (**A**) sgRNA5-1 + sgRNA6-1, (**B**) sgRNA5-2 + sgRNA6-1, (**C**) sgRNA5-1 + sgRNA6-2, and (**D**) sgRNA5-2 + sgRNA6-2. (**E-H**) Intron 5-6 proximal inversion-breakpoints (see **Fig. 1C**, middle panel) generated by CRISPR/Cas9 sgRNA pairs: (**E**) sgRNA5-1 + sgRNA6-1, (**F**) sgRNA5-2 + sgRNA6-1, (**G**) sgRNA5-1 + sgRNA6-2, and (**H**) sgRNA5-2 + sgRNA6-2. (**I-L**) Intron 5-6 proximal inversion-breakpoints (see **Fig. 1C**, lower panel) generated by CRISPR/ Cas9 sgRNA pairs: (**I**) sgRNA5-1 + sgRNA6-1, (**J**) sgRNA5-2 + sgRNA6-1, (**K**) sgRNA5-1 + sgRNA6-2, and (**L**) sgRNA5-2 + sgRNA6-2. Key: Blue text, intron 5 sequence (or reverse complement); purple text, intron 6 sequence (or reverse complement); red text, ambiguous (either intron 5 or intron 6) or inserted sequence at breakpoint; yellow shade, proximal sgRNA 5-1 or 5-2 sequence; blue shade, distal sgRNA 6-1 or 6-2 sequence; gray shade, PAM sequences for each sgRNA target; Backslash ("/") character, expected breakpoint in reference sequence.

Supplemental Figure S4. Fluorescence *in situ* hybridization (FISH) mapping of the pig *PAH-IGF1* locus.

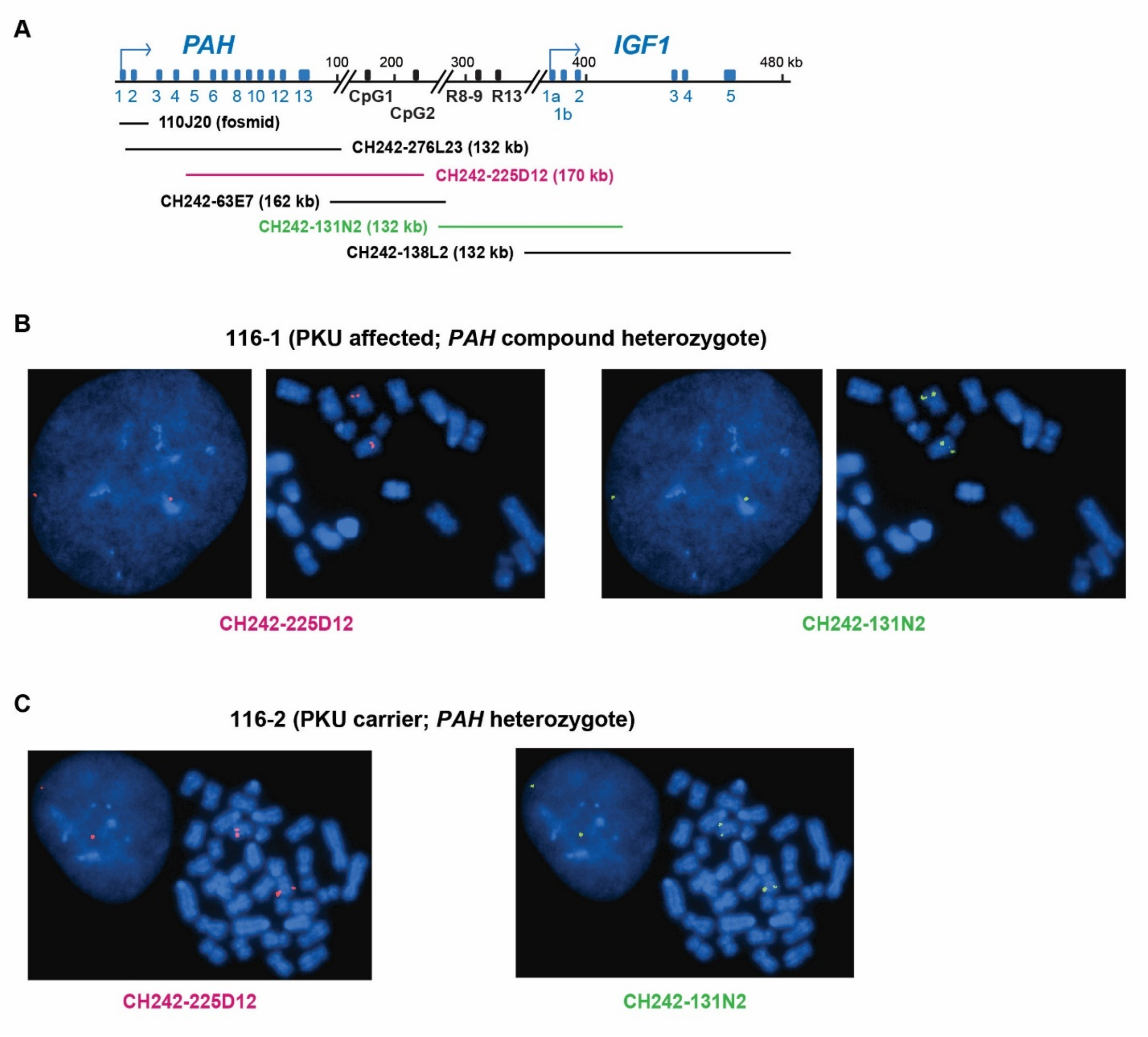

Supplemental Figure S4. Fluorescence *in situ* hybridization (FISH) mapping of the pig *PAH-IGF1* locus. (**A**) BAC contig and gene map of the *PAH-IGF1* interval. Sequence tagged sites (STS) in the intergenic region used for PCR amplicons are indicated by black rectangles: CpG1 and CpG2 are CpG-rich sequence elements, while R8-9 and R13 are within a subset of *IGF1*-enhancer elements (*1*). The two BACs used for FISH are color coded. (**B-C**) Representative interphase (left) and metaphase (right) FISH images for CH242-225D12 and CH242-131N2 using fibroblasts from

(**B**) 116-1 (PKU affected) and (**C**) 116-2 (PKU heterozygote), demonstrating that both alleles of each BAC clone are intact in cell lines from each of the founder PKU pigs. 1000x total magnification.

Supplemental Figure S5. Representative 7-month old pig brain MRI images from control and PKU affected animal 116-1.

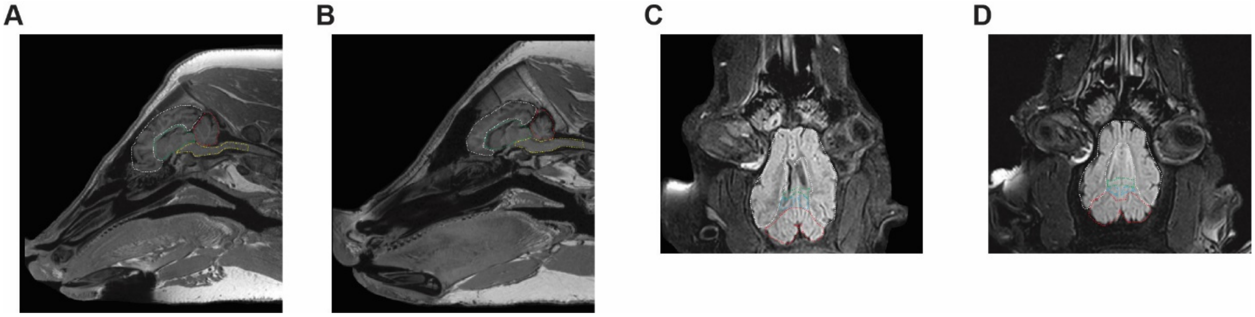

Supplemental Figure S5. Representative 7-month old pig brain MRI images from control and PKU affected animal 116-1. (**A,B**) T1 weighted sagittal view of (**A**) control and (**B**) affected PKU pigs. (**C,D**) T2 weighted axial view of (**C**) control and (**D**) PKU affected pig. Key: dashed lines show segmentation for cortex (white), diencephalon (green), hippocampus (blue), cerebellum (red) and brainstem (yellow).

Supplemental Figure S6. Mapping of *PAH* deletion extents by genomic DNA droplet-digital PCR (ddPCR) in the pig PKU founder animals.

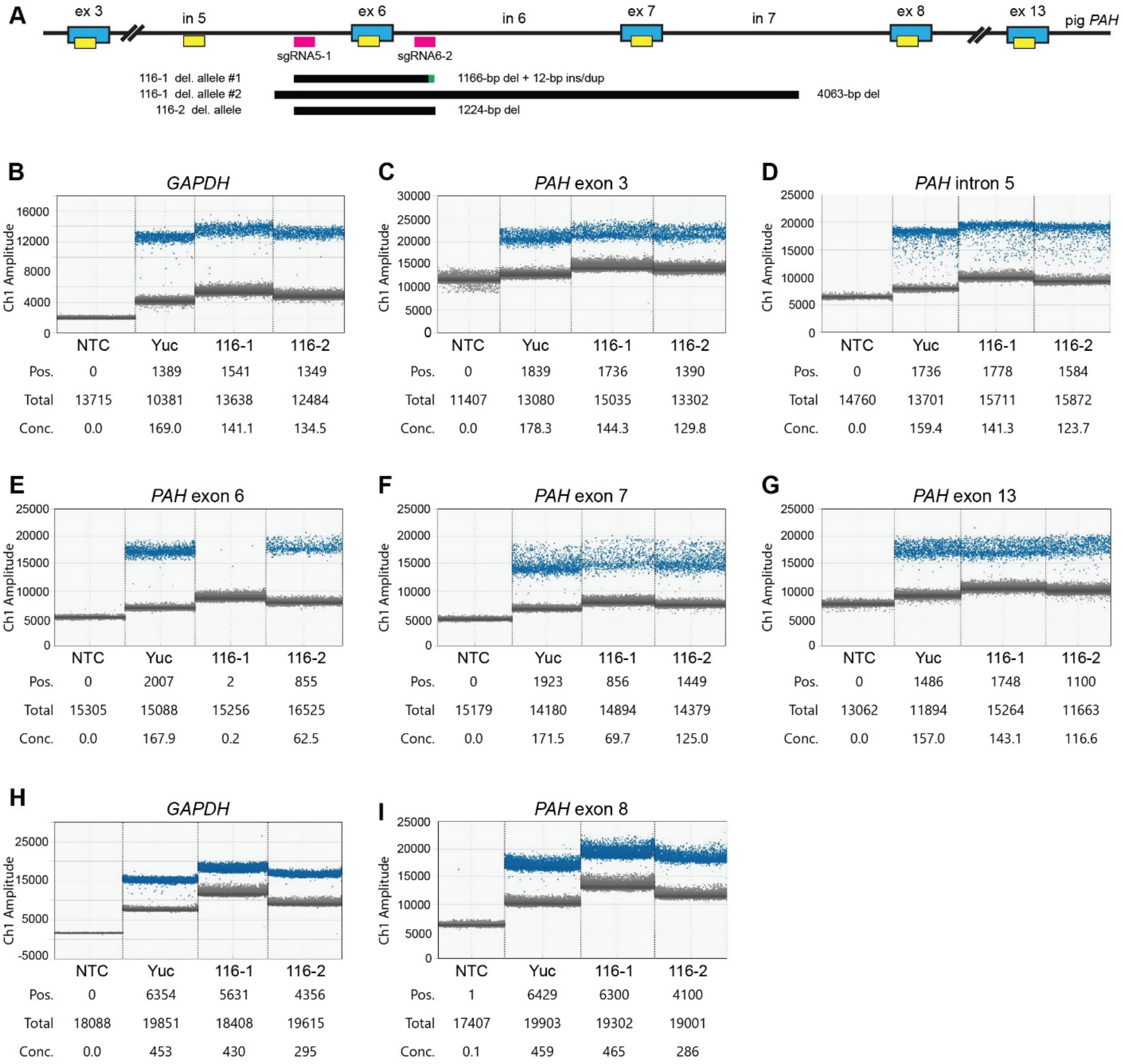

Supplemental Figure S6. Mapping of *PAH* deletion extents by genomic DNA droplet-digital PCR (ddPCR) in the pig PKU founder animals. (**A**) Schematic of the *PAH* locus showing location of ddPCR amplicons with respect to exons (ex) and introns (in). The genome-editing target sites and extent of deletions determined by molecular approaches are also illustrated. Key: Blue box, *PAH* exons with intervening introns; Pink box, CRISPR sgRNA sites; Yellow box,

ddPCR amplicons; black bars, region deleted; green bar, insertion/duplication. (**B-I**) Representative 1-D amplitude plots for *GAPDH* as a control, and for six *PAH* genomic amplicons in 116-1, 116-2, Yucatan (Yuc) control, and no template control (NTC). Experiments were performed using 5 ng (**B-G**) or 25 ng (**H**,**I**) of input genomic DNA. Blue dots represent positive droplets whereas black dots represent negative droplets. Below each 1-D amplitude plot measuring absorbance of Evagreen as a proxy for genomic amplification the numbers of positive (Pos.) and accepted (Total) droplets and the concentration (Conc.) in copies/μl of each PCR amplicon per sample are shown.

Supplemental Figure S7. Sanger sequencing chromatograms spanning deletion breakpoints and scarred alleles in the founding PKU pigs.

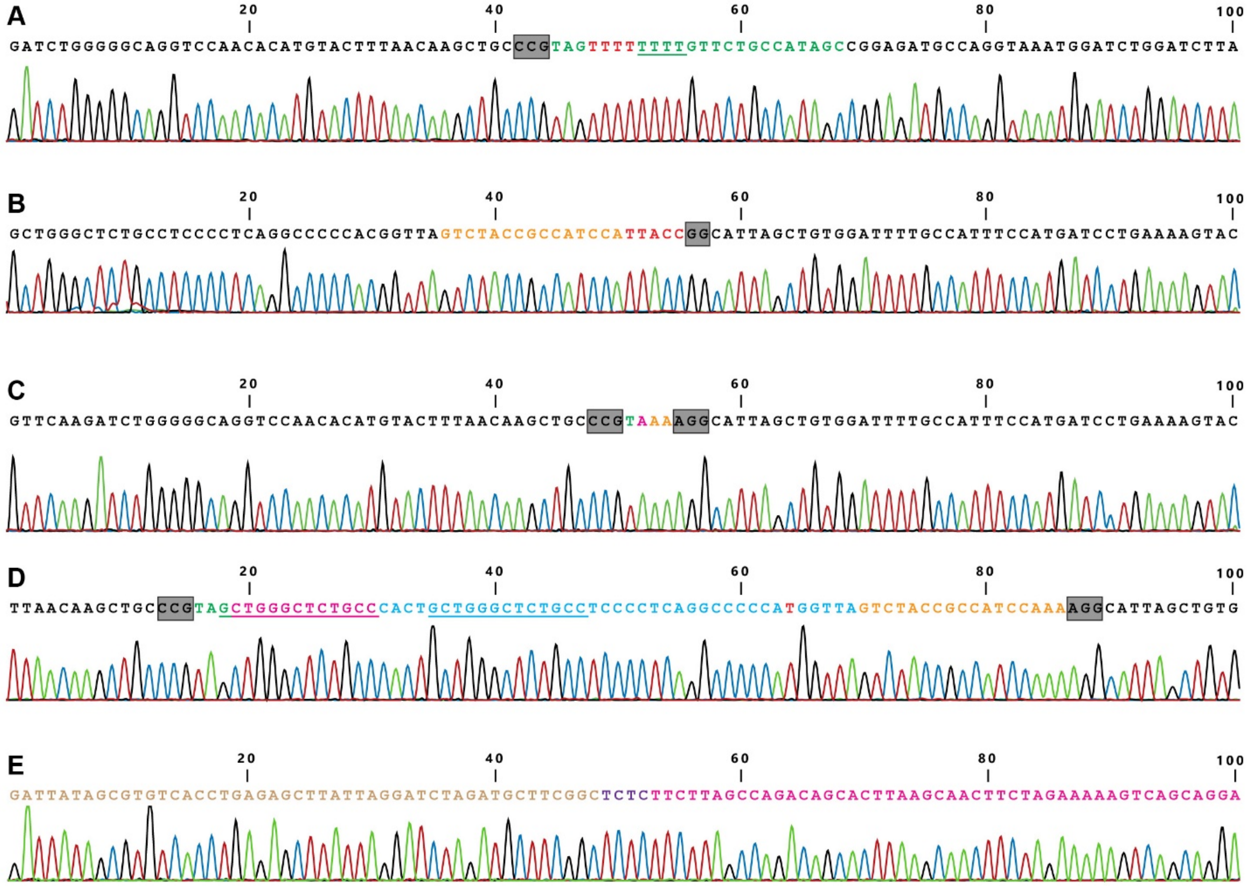

Supplemental Figure S7. Sanger sequencing chromatograms spanning deletion breakpoints and scarred alleles in the founding PKU pigs. (**A**) 116-2 sgRNA5-1 scarred allele within *PAH* intron 5. (**B**) 116-2 sgRNA6-2 scarred allele within *PAH* intron 6. (**C**) 116-2 deletion allele (exon 6). (**D**) 116-1 deletion allele-1 (exon 6). (**E**) 116-1 deletion allele-2 (exon 6-7).

Supplemental Figure S8. DNA sequence analyses for the top predicted sgRNA5-1 off-target sites.

A. 5-1 Off-target #1 Chr4 GCTATATCAGAACAAAACTAGAG

Yucatan (Control)

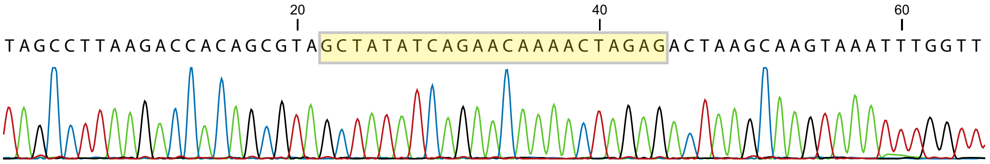

116-1 (PKU affected)

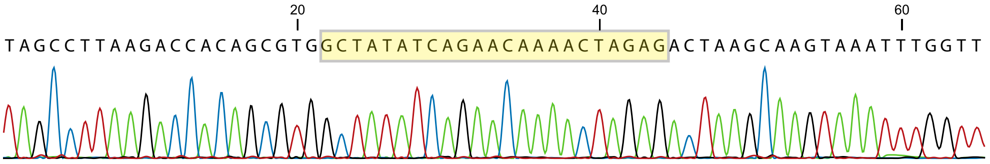

116-2 (PKU carrier)

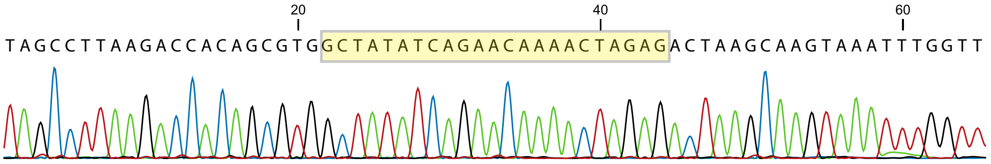

B. 5-1 Off-target #2 Chr16 AATATGCCAGAACAAAACTATGG

Yucatan (Control)

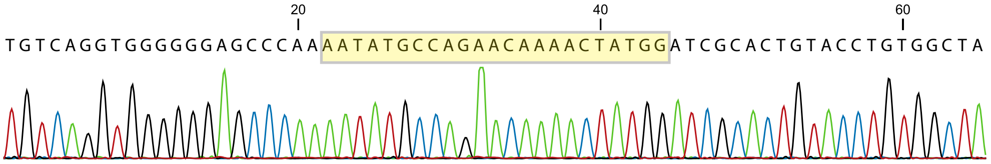

116-1 (PKU affected)

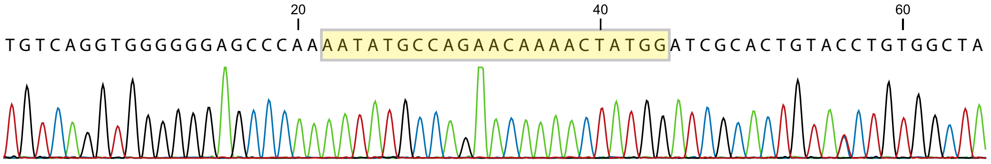

116-2 (PKU carrier)

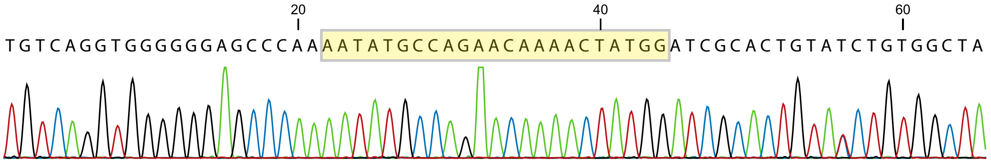

C. 5-1 Off-target #3 Chr13 TCCTGGGCAGAACAAAACTAAAG

Yucatan (Control)

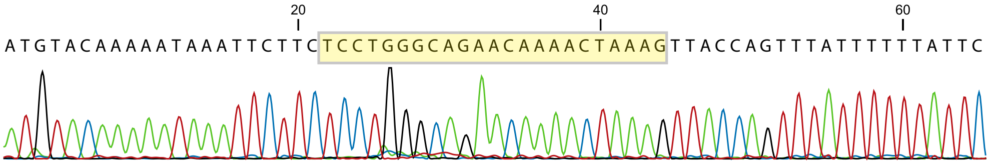

116-1 (PKU affected)

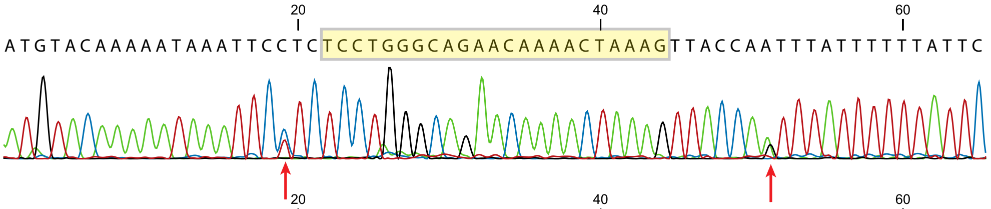

116-2 (PKU carrier)

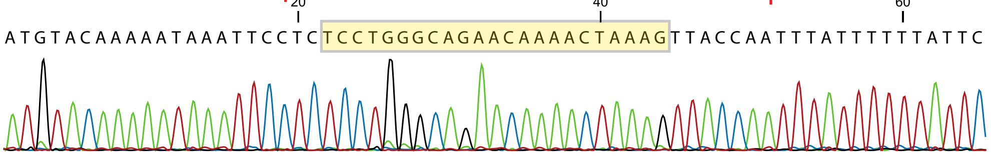

Supplemental Figure S8 (cont.)

D. 5-1 Off-target #4 Chr14 AAAGTGGCAGAACAAAACTAGAG

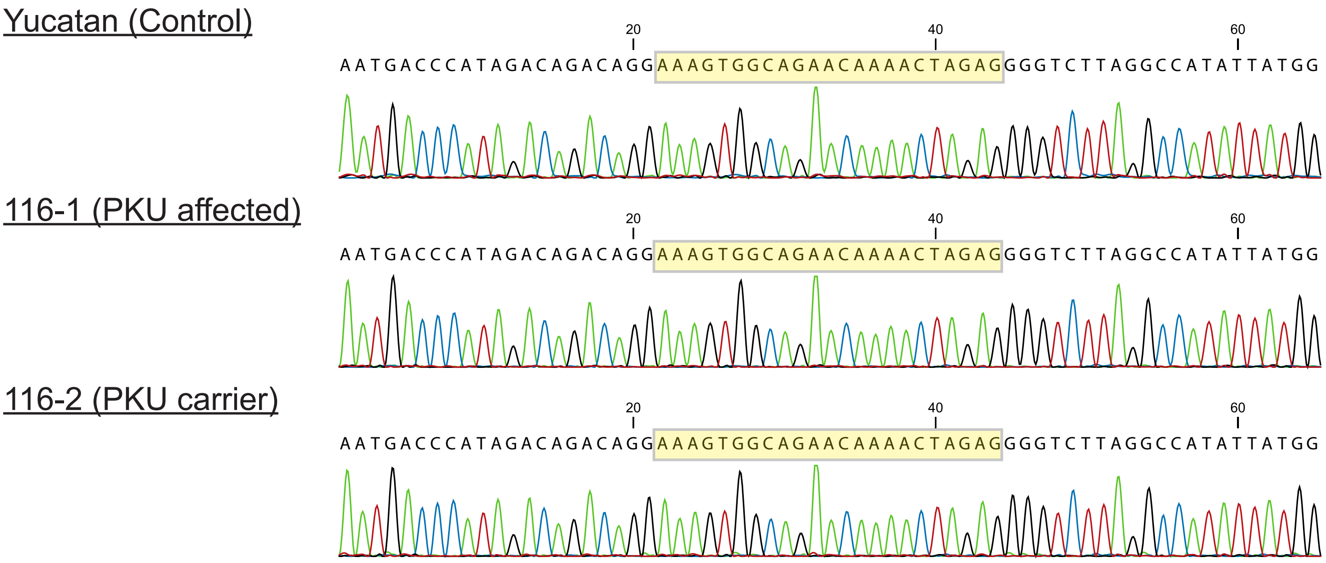

E. 5-1 Off-target #5 Chr6 GTTTTGCAAGAACAAAACTAAAG

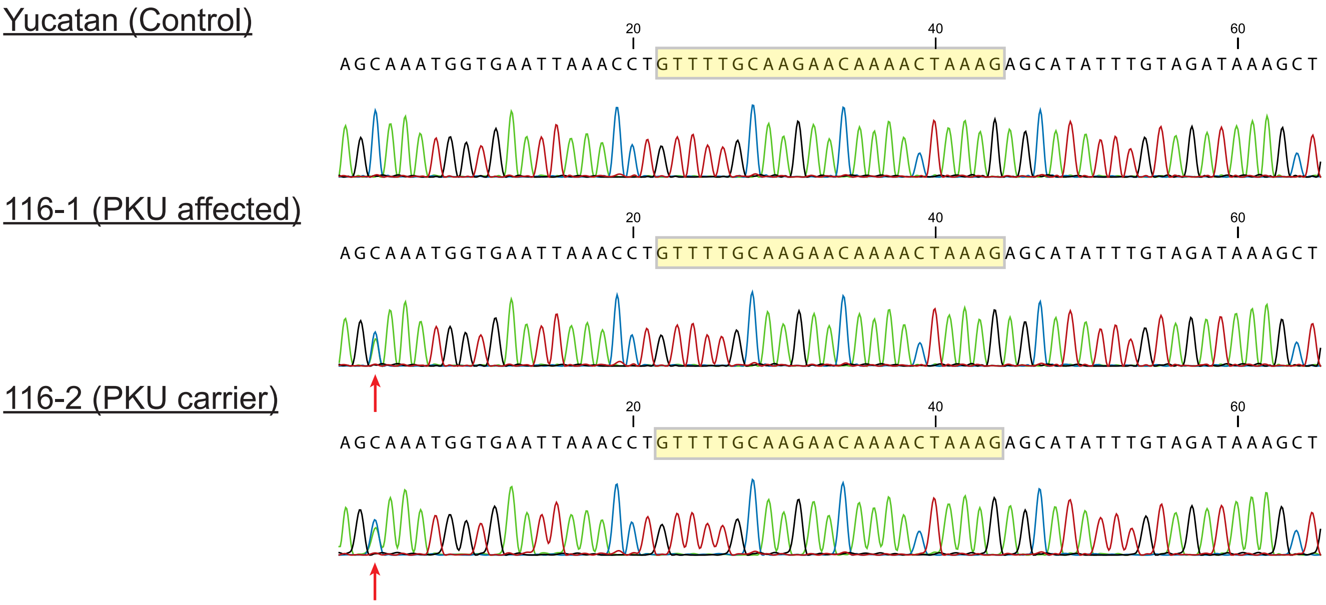

F. 5-1 Off-target #6 Chr13 CGGATGCCAGAACAAAACTACAG

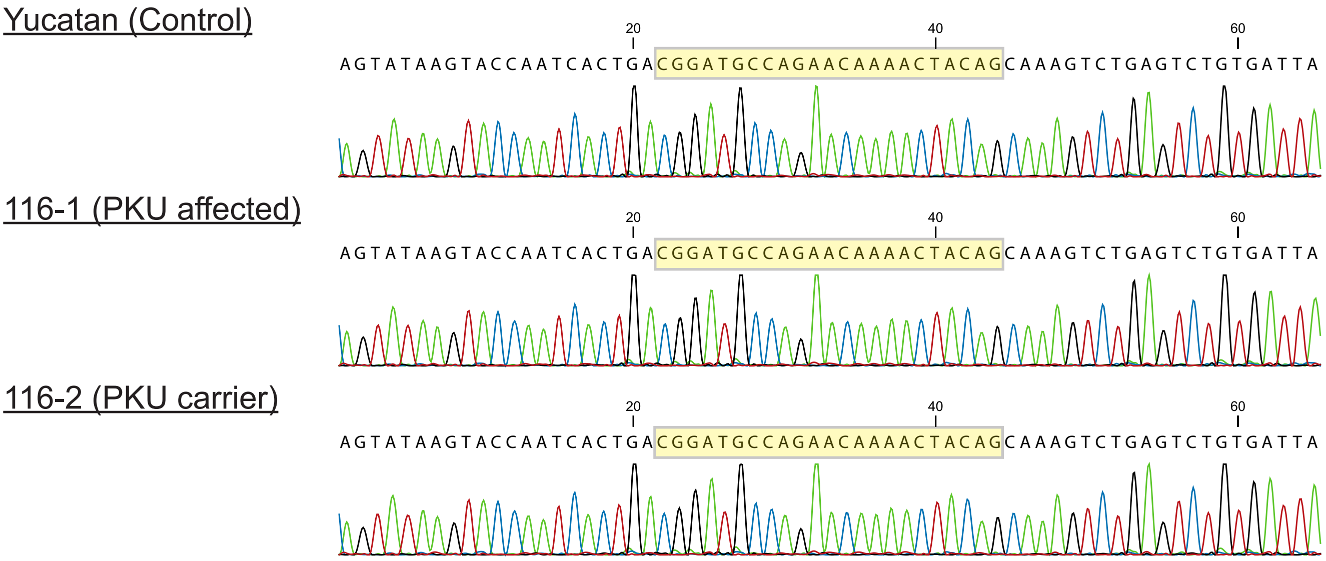

Supplemental Figure S8 (cont.)

G. 5-1 Off-target #7 GL892891.1 GATCTAGAAGAACAAAACTAAGG

Yucatan (Control)

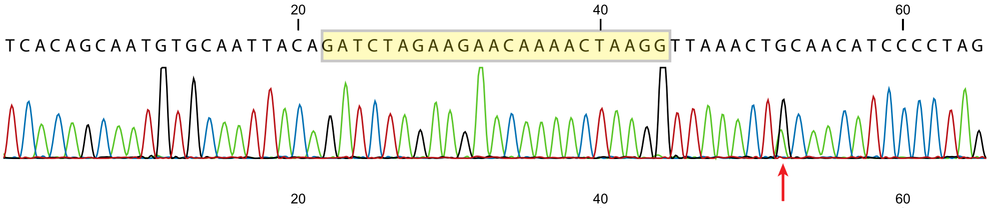

116-1 (PKU affected)

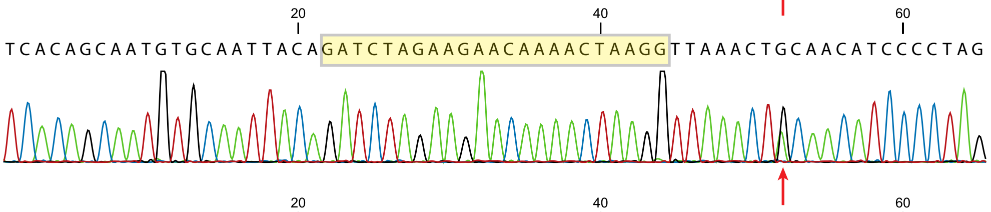

116-2 (PKU carrier)

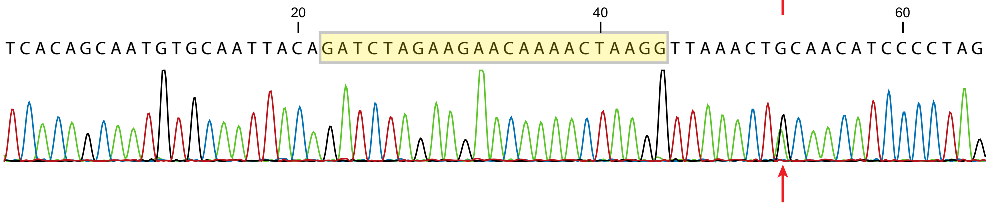

H. 5-1 Off-target #8 Chr8 TCAATGCCAGAAGAAAACTAAAG

Yucatan (Control)

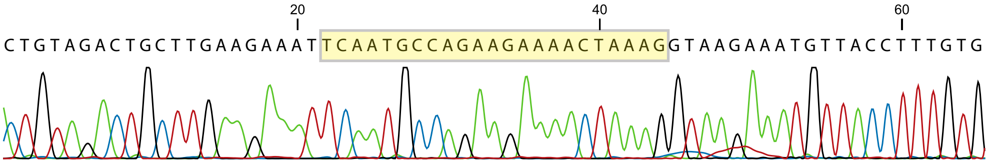

116-1 (PKU affected)

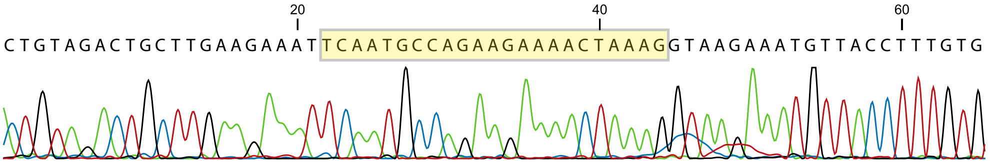

116-2 (PKU carrier)

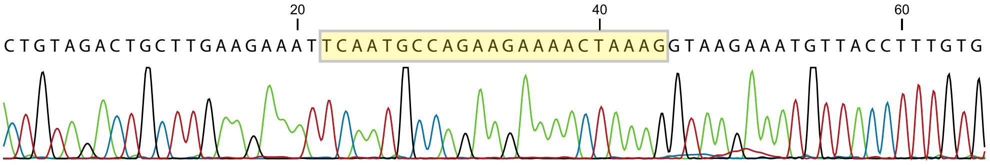

Supplemental Figure S8. DNA sequence analyses for the top predicted off-target sites for sgRNA5-1. A-H: For each panel, at top is shown the off-target rank, chromosome location, and DNA sequence of the 23-nt sequence matching the 20-nt guide and 3-nt PAM. Underneath are the DNA sequence traces produced by genomic PCR and direct sequencing, for Yucatan control (top), 116-1 compound heterozygous pig (middle) and 116-2 heterozygous carrier pig (bottom) with the off-target site highlighted in yellow. SNPs distinguishing alleles are noticeable by dual peaks and are marked by red arrows. The following SNPs were identified: Two SNPs (C/T and G/A) bordering sgRNA5-1 off-target #3 (**Fig. S7C**) were heterozygous in 116-1; a heterozygous SNP (C/A) upstream of sgRNA5-1 off-target #5 (**Fig. S7E**) shared by both affected and carrier PKU pigs while being a unambiguous C in Yucatan; and a heterozygous polymorphism (G/A) in 116-1, 116-2 and Yucatan that was present 8-nt distal of sgRNA5-1 off-target #7 (**Fig. S7G**). Notably there were no indel mutations at any of the eight potential off-targets which would be expected from NHEJ repair of CRISPR/Cas9 nucleolytic cleavage. Off-target sites were predicted using the original MIT CRISPR sgRNA design program based on *S. scrofa* genome build 10.2, however, two predicted top ranking off-target sites were derived from mis-assembly of genome sequence and were excluded from the analysis. One of these resulted from a duplicated on-target *PAH* locus sequence assembly error, and the second at a sequence contig assembled on chromosome 13 and incorrectly on chromosome X.

Supplemental Figure S9. DNA sequence analyses for the top predicted sgRNA6-2 off-target sites.

A. 6-2 Off-target #1 Chr1 AGCTCCCTCCATCCAAGAAAAGG

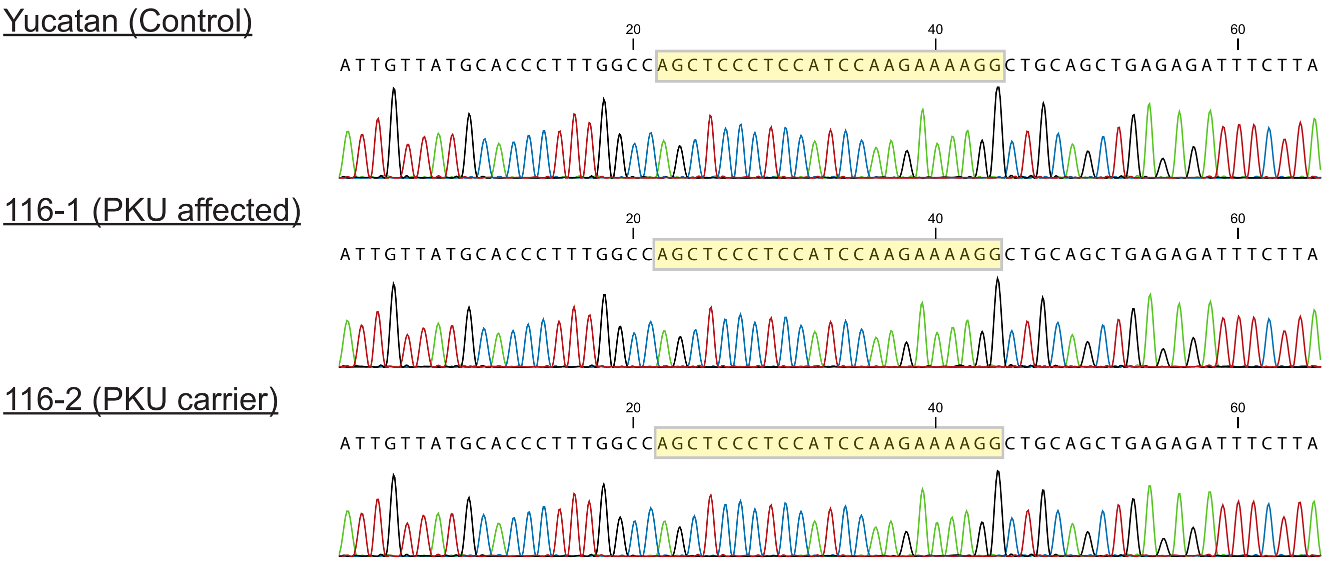

B. 6-2 Off-target #2 Chr11 GGACACCACCATCCAAGAAACGG

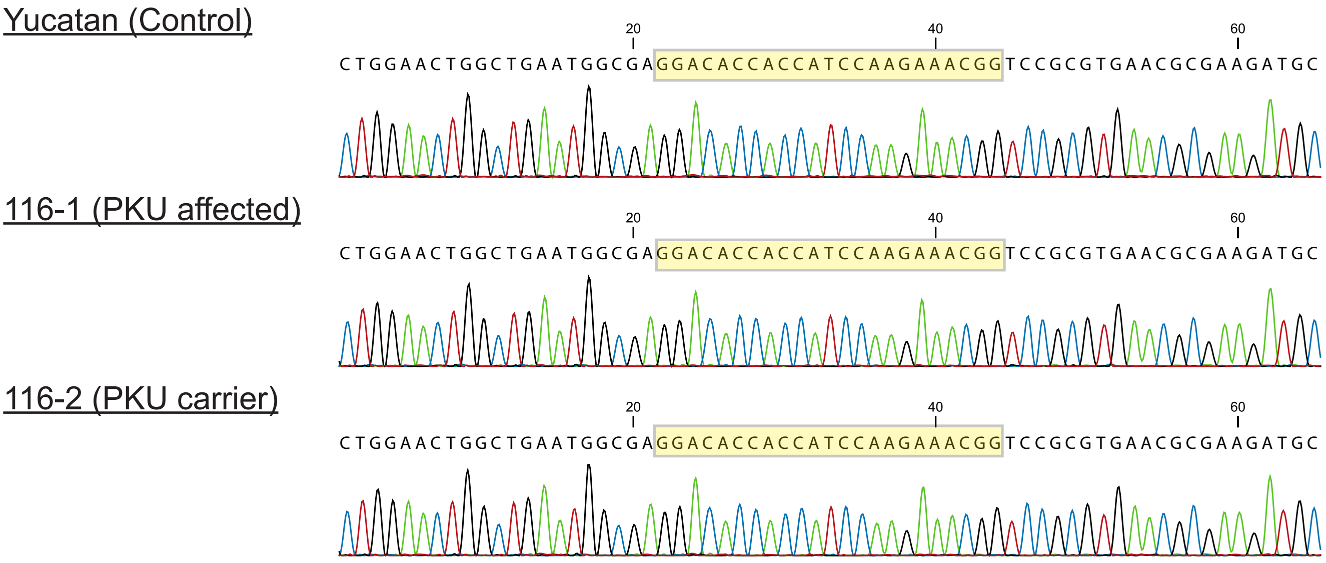

C. 6-2 Off-target #3 Chr9 GTATCCCCCAATCCAAGAAAAGG

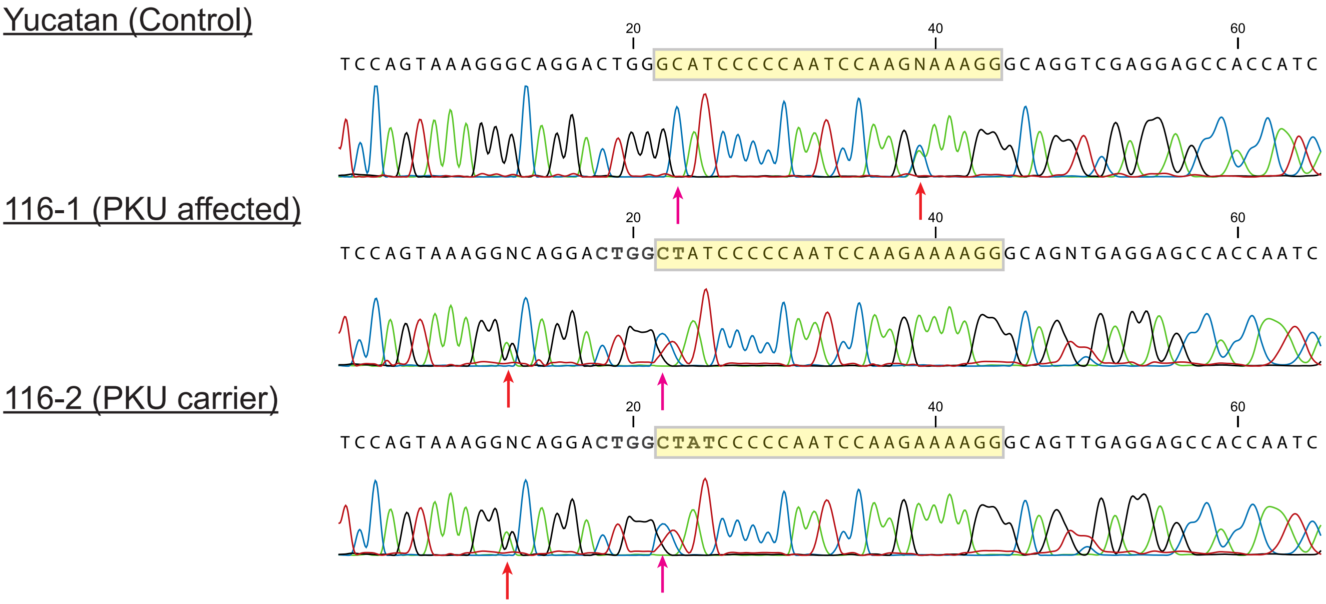

Supplemental Figure S9 (cont.)

D. 6-2 Off-target #4 Chr7 GTCTGCTGTCATCCAAGAAAAAG

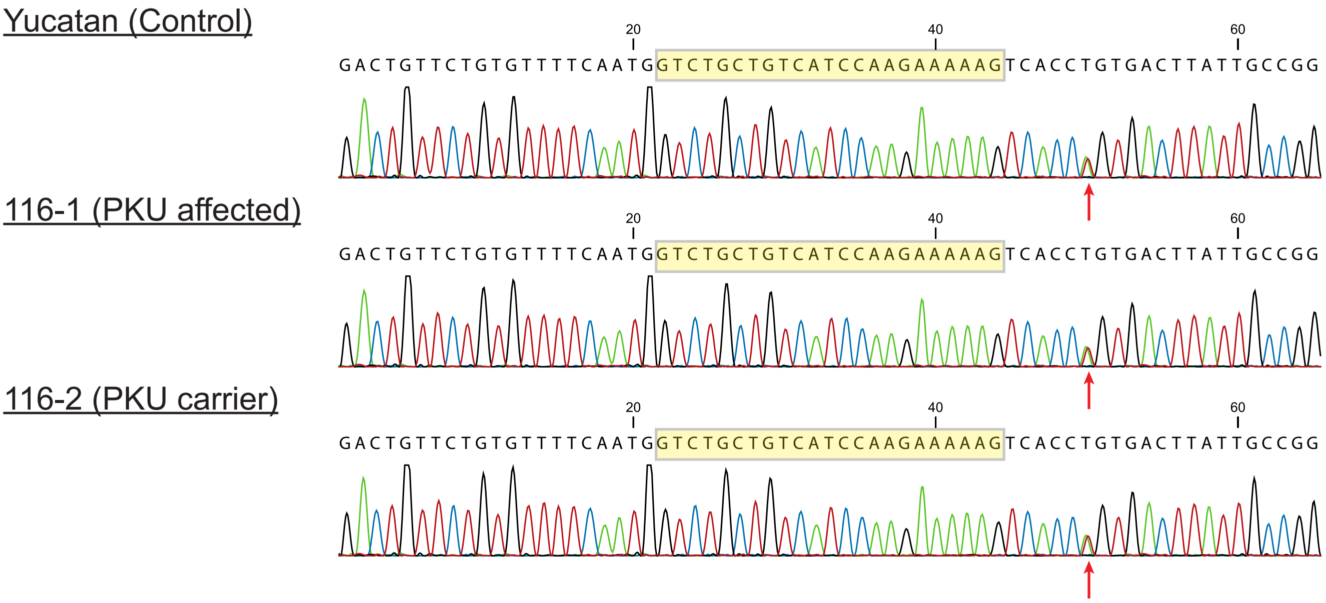

E. 6-2 Off-target #5 Chr9 TTCATCTGCCATCCAAGAAACAG

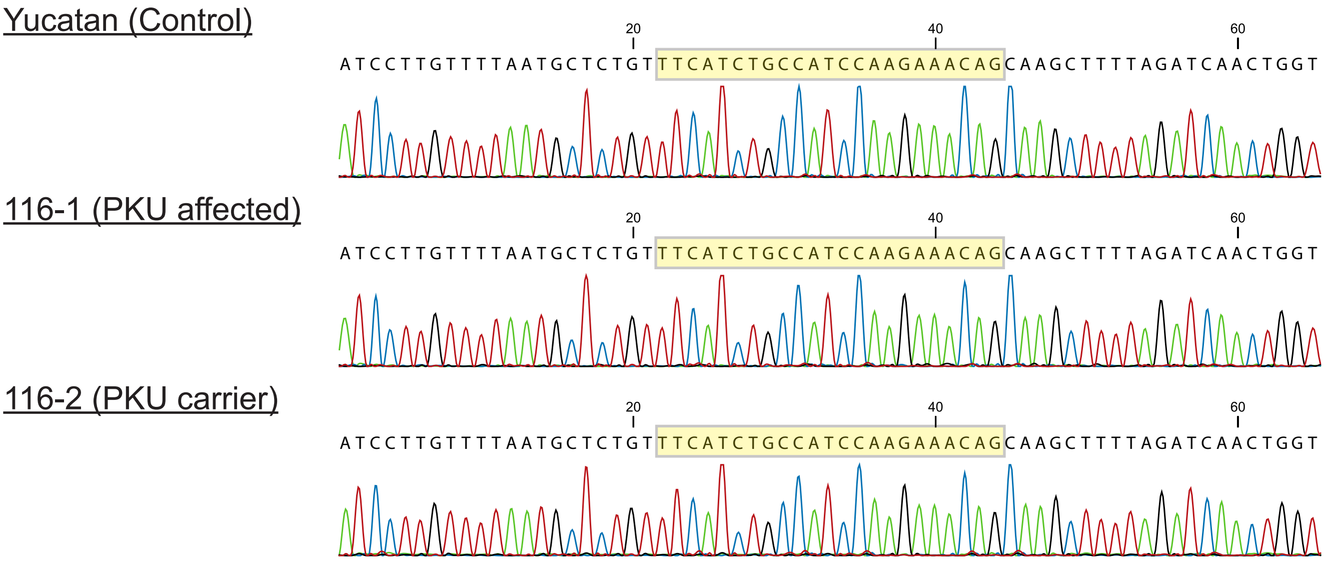

F. 6-2 Off-target #6 Chr7 GGCCACGCCCATCCAAGAAATAG

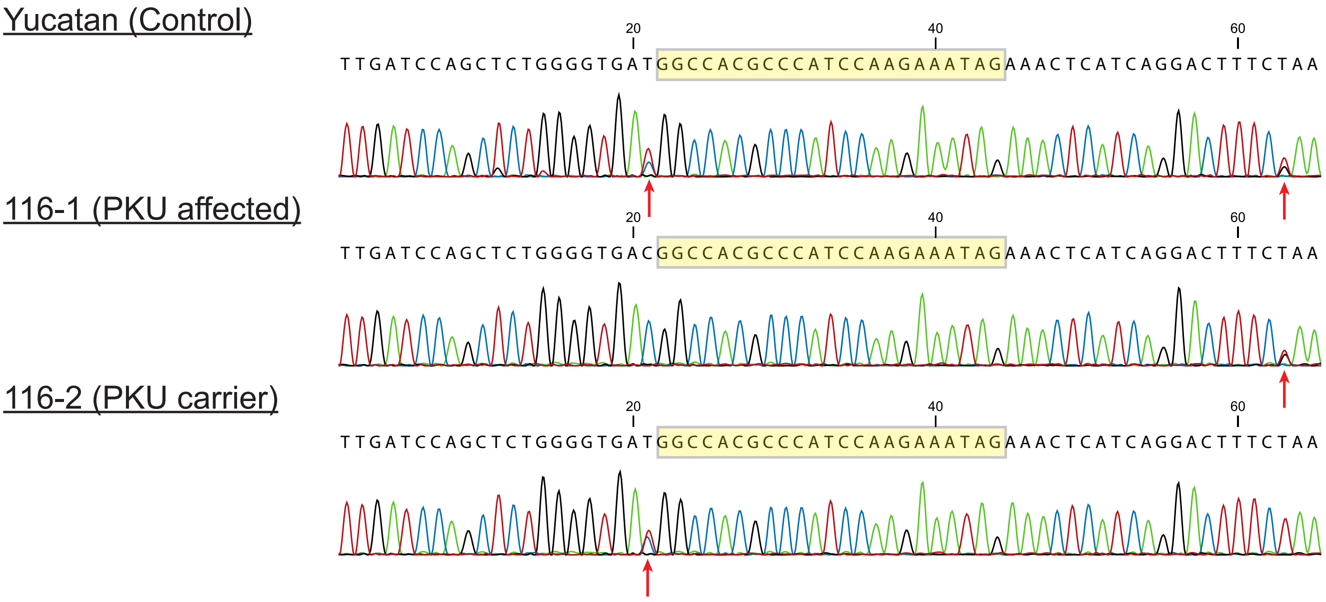

Supplemental Figure S9 (cont.)

G. 6-2 Off-target #7 Chr4 GTCTCCCGCAATCCAAGAGAAAG

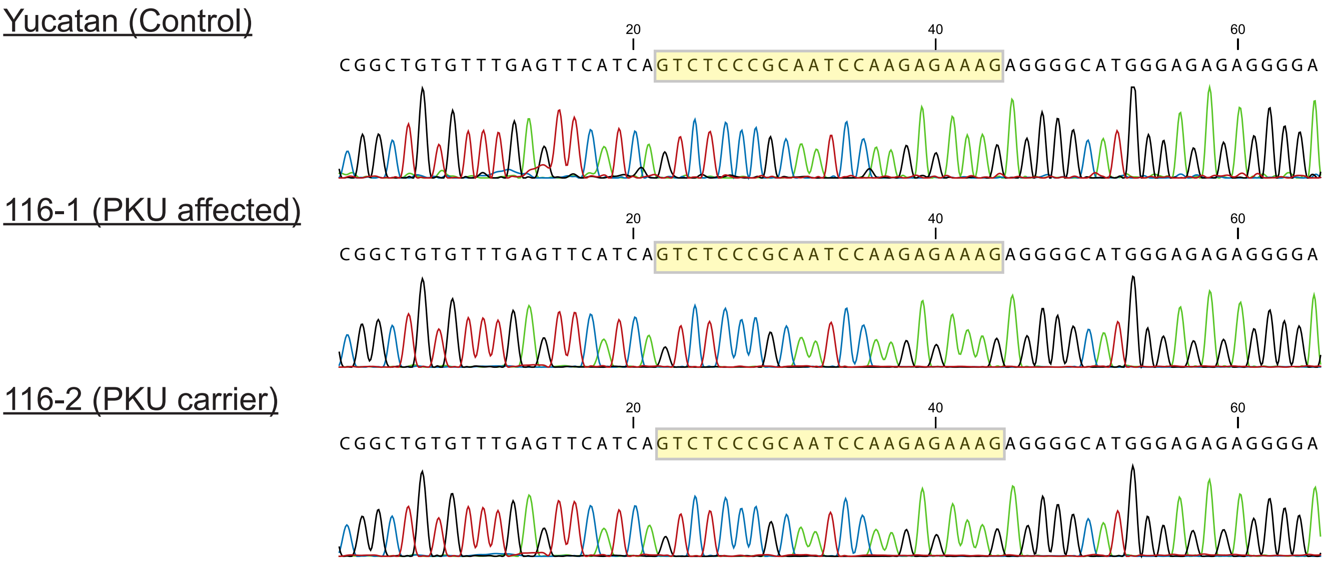

H. 6-2 Off-target #8 Chr2 GGCTTCCTCCAACCAAGAAAGAG

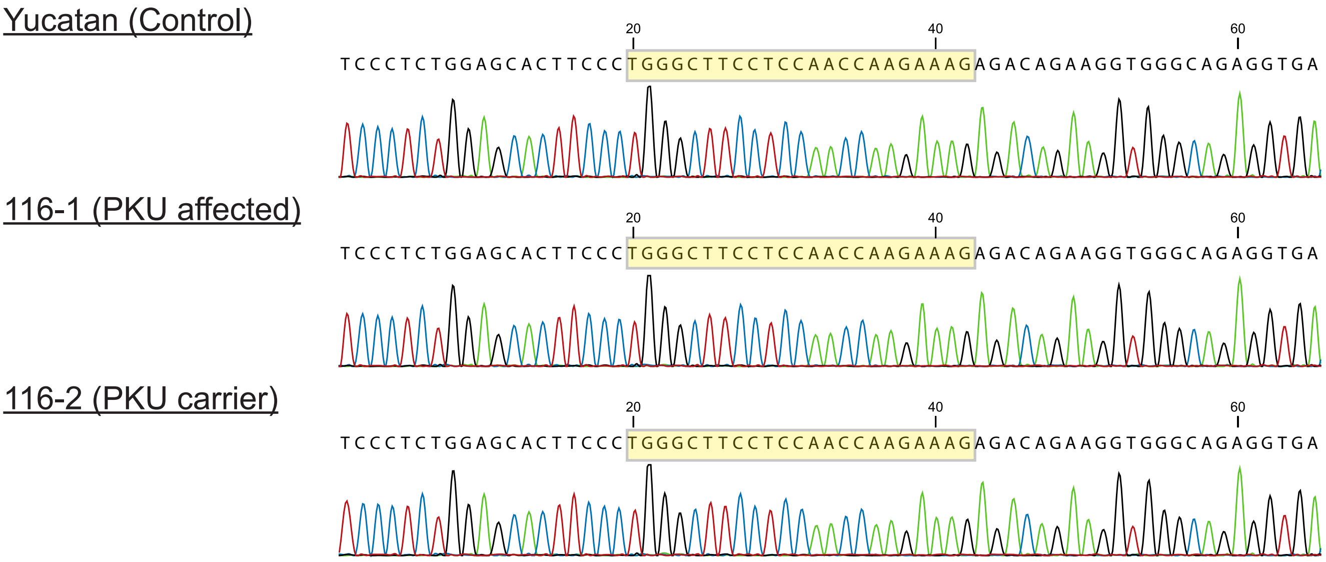

I. 6-2 Off-target #9 Chr14 GCCTCCAGGCATCCAAGAAATGG

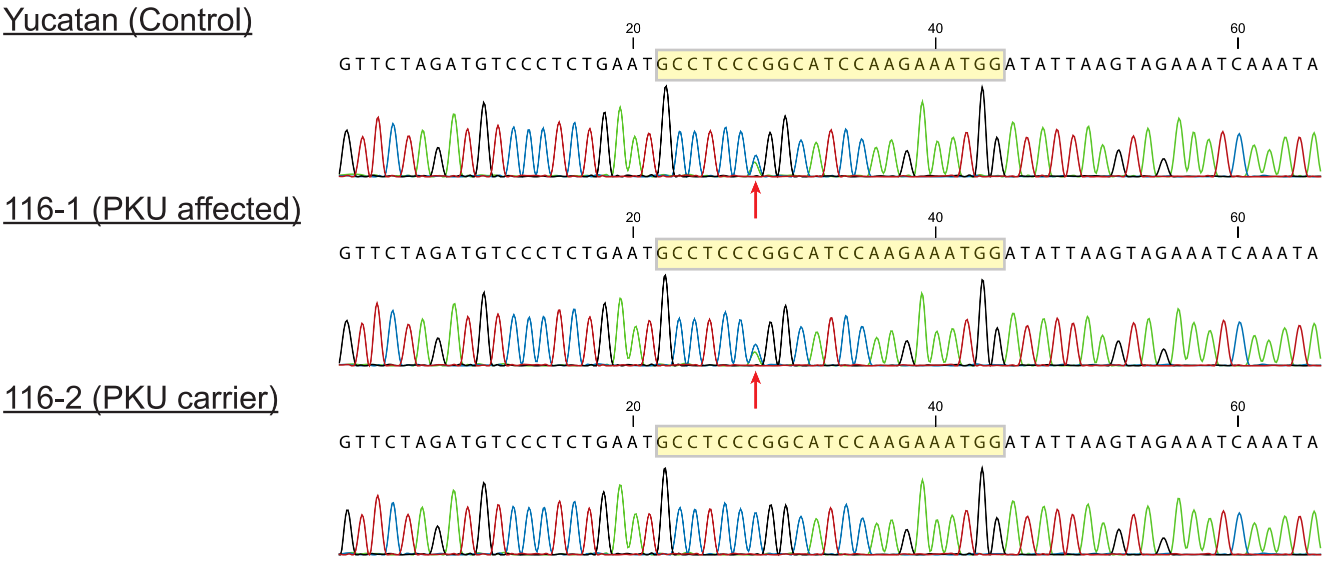

Supplemental Figure S9 (cont.)

J. 6-2 Off-target #10 Chr1   GTTTAAATCCATCCAAGAAATAG

Yucatan (Control)

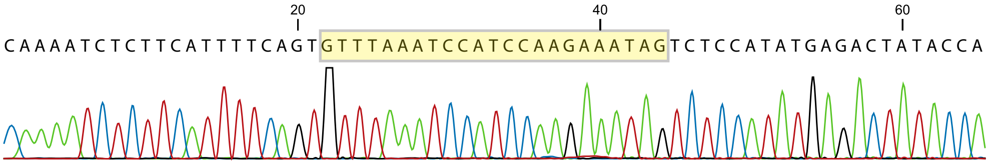

116-1 (PKU affected)

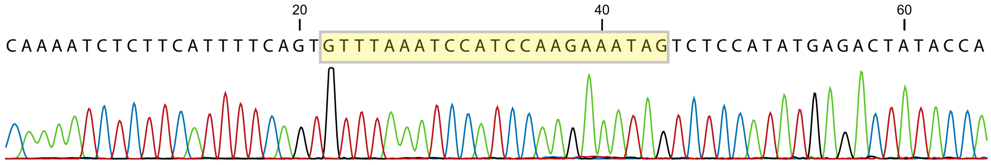

116-2 (PKU carrier)

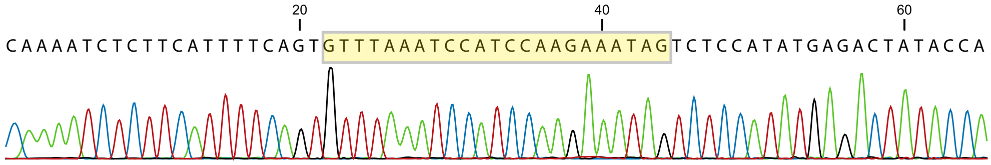

Supplemental Figure S9. DNA sequence analyses for the top predicted off-target sites for sgRNA6-2. A-J: For each panel, the off-targets, SNPs and DNA sequence traces from genomic PCR and direct DNA sequencing for Yucatan control (top), 116-1 compound heterozygous pig (middle) and 116-2 heterozygous pig (bottom) are presented as for **Fig. S7** with the addition of magenta arrows indicating homozygous polymorphisms at proximal sgRNA6-2 off-target #3. Polymorphisms were detected in the following potential sgRNA6-2 off-targets sites: For 6-2 off-target #3 (**Fig. S8C**) there are a total of four polymorphisms, including a heterozygous SNP (G/A) upstream of the off-target in 116-1 and 116-2 which was an unambiguous G in Yucatan; then there are adjacent homozygous polymorphisms at the off-target positions 1 (G or C) and 2 (C or T) with GT for the reference genome (Duroc), GC for Yucatan, and CT at positions 1-2 for the two PKU founder pigs; and a heterozygous SNP (C/A) in Yucatan at the off-target position 18 that is an unambiguous A in both PKU founder pigs. Additionally there was a heterozygous SNP (A/T) present distal to 6-2 off-target #4 present in Yucatan and the two founding PKU pigs (**Fig. S8D**); a heterozygous SNP (C/T) in the Yucatan and 116-2 1-nt upstream of sgRNA6-2 off-target #6 which was an unambiguous C in 116-1, as well as a heterozygous SNP (T/G) in Yucatan and 116-1 distal of sgRNA6-2 off-target #6 which was an unambiguous T in 116-2 (**Fig. S8F**); and a heterozygous SNP (C/A) in Yucatan and 116-1 at sgRNA6-2 off-target #9 position 7 which was an unambiguous C in 116-2 (**Fig. 8I**). Given that the polymorphisms detected mostly occur outside the off-target sites and/or also in the Yucatan control, they are likely natural variations and not induced by CRISPR/Cas9. Furthermore, there were no indel mutations at the canonical sgRNA position 17 Cas9 preferred nuclease site that might be predicted to occur during off-target genome-editing; the only SNP at this position occurred in the Yucatan control.

Supplemental Figure S10. Alignment of paired-end reads from 116-1 overlapping RS1108162262 in the homopolymer 10A/11A SNP.

A.

```
>GWNJ-0965:486:GW1909262516th:4:1102:26007:43378
GGTTTTCAAAAGGAACTTTCGAACTAATGACTCTCAGATTATAGCGTGTCACCTGAGAGCTTATTAGGATCTAGATG
CTTCGGCTCTCTTCTTAGCCAGACAGCACTTAAGCAACTTCTAGAAAAAGTCAGCAGGACATCCCAAATGATCTTTC
CCTGCCCGCAAAGTTCATTCCAATAAATAAAAAAAAAAAGGCCTGTTCTTCATAACCTGGCACATCAAGTGTTCAAT
AAATGCAGGTTTTCTTCTCTGCCCAATGGCTGCCCCTCC
```

```
>GWNJ-0965:486:GW1909262516th:4:2102:3417:56106
AAGGAACTTTCGATGGTTTTCAAAAGGAACTTTCGAACTAATGACTCTCAGATTATAGCGTGTCACCTGAGAGCTTA
TTAGGATCTAGATGCTTCGGCTCTCTTCTTAGCCAGACAGCACTTAAGCAACTTCTAGAAAAAGTCAGCAGGACATC
CCAAATGATCTTTCCCTGCCCGCAAAGTTCATTCCAATAAATAAAAAAAAAAAGGCCTGTTCTTCATAACCTGGCAC
ATCAAGTGTTCAATAAATGCAGGTTTTCTTCTCT
```

B.

```
>GWNJ-0965:486:GW1909262516th:4:1115:24109:8447
AATCCGCACCTAGATGACTCCGCTGTCTCTCGGCCTGGATCGGGCTCGGCCGGAGTAGAGGAGGACTGGGCAGGTGC
TTCTTACCTTTGCACTTAGATCTCTTCTTAGCCAGACAGCACTTAAGCAACTTCTAGAAAAAGTCAGCAGGACATCC
CAAATGATCTTTCCCTGCCCGCAAAGTTCATTCCAATAAATAAAAAAAAAAGGCCTGTTCTTCATAACCTGGCACAT
CAAGTGTT
```

```
>GWNJ-0965:486:GW1909262516th:4:1209:21359:12332
GCCGGAGTAGAGGAGGACTGGGCAGGTGCTTCTTACCTTTGCACTTAGATCTCTTCTTAGCCAGACAGCACTTAAGC
AACTTCTAGAAAAAGTCAGCAGGACATCCCAAATGATCTTTCCCTGCCCGCAAAGTTCATTCCAATAAATAAAAAAA
AAAGGCCTGTTCTTCATAAC
```

```
>GWNJ-0965:486:GW1909262516th:4:2121:5355:69344
CCAAGAGACTGTAGAAGTCAGCGCTAAGGAGCGTCCAGTGGGAACAGGAGCAAGGGATGGCCAGGCCAGCCCCCCAC
CTCCTGAATCCGCACCTAGATGACTCCGCTGTCTCTCGGCCTGGATCGGGCTCGGCCGGAGTAGAGGAGGACTGGGC
AGGTGCTTCTTACCTTTGCACTTAGATCTCTTCTTAGCCAGACAGCACTTAAGCAACTTCTAGAAAAAGTCAGCAGG
ACATCCCAAATGATCTTTCCCTGCCCGCAAAGTTCATTCCAATAAATAAAAAAAAAAG
```

Supplemental Figure S10. Alignment of paired-end reads from 116-1 overlapping RS1108162262 in the homopolymer 10A/11A SNP. (**A**) Two independent paired-end reads cover the microhomology mediated intron5-intron7 breakpoint in 116-1 allele 2 (exon 6-7 deletion) and RS1108162262 11A reference alternate variant sequence. (**B**) Three unique paired-end reads that overlap the 10A RS1108162262 variant and contiguous sequence surrounding the intron 7 TCTC microhomology region without a breakpoint. Key: Joined paired-end fastq read sequence as text with intron 5 in blue and intron 7 in purple, overlapping portions of paired-end

sequences underlined, TCTC microhomology highlighted in magenta, and 10A/11A polymorphism highlighted in yellow and red, respectively.

Supplemental References

1. D. J. Chia, B. Varco-Merth, P. Rotwein, Dispersed chromosomal Stat5b-binding elements mediate growth hormone-activated insulin-like growth factor-I gene transcription. *J Biol Chem* **285**, 17636-17647 (2010).
